# Supplementary material for: Autoreactive T cells target peripheral nerves in Guillain–Barré syndrome
Source: Nature. 2024 Jan 17;626(7997):160–8. doi: 10.1038/s41586-023-06916-6 (PMC10830418; doi:10.1038/s41586-023-06916-6)
Supplement: Supplementary file 1 — This file contains Supplementary Tables 1–5. [file 41586_2023_6916_MOESM1_ESM.pdf]

nature portfolio
https://doi.org/10.1038/s41586-023-06916-6

Supplementary information

Autoreactive T cells target peripheral nerves in Guillain–Barré syndrome

In the format provided by the authors and unedited

Supplementary Table 1

Comparative analyses of relevant gene expression displayed in Figure 2. Detailed *P* values of comparative gene expression analyses displayed in Figure 2a, b, and d.

Gene	Aantigen reactive vs Non reactive	PNS-myelin reactive vs Flu Reactive	Significance
FOXM1	*P* ≤ 2e-16		yes
PLK1	*P* ≤ 2e-16		yes
BUB1	*P* ≤ 2e-16		yes
CCNB1	*P* ≤ 2e-16		yes
TOP2A	*P* ≤ 2e-16		yes
MKI67	*P* ≤ 2e-16		yes
NME1	*P* ≤ 2e-16		yes
ITGAE	*P* ≤ 2e-16		yes
ITGA1	*P* = 3.2e-10		yes
TNFRSF4	*P* ≤ 2e-16		yes
TNF	*P* ≤ 2e-16		yes
HLA-DRA	*P* ≤ 2e-16		yes
ICOS	*P* ≤ 2e-16		yes
IL2RA	*P* ≤ 2e-16		yes
KIR2DL3		*P* = 0.45	no
KIR2DL1		*P* = 0.45	no
FCGR3A		*P* = 0.29	no
FGFBP2		*P* = 4e-11	yes
KLRF1		*P* = 0.48	no
CX3CR1		*P* = 3.9e-5	yes
ADGRG1		*P* = 0.039	yes
ZEB2		*P* = 0.00015	yes
ZNF683		*P* = 0.17	no
EOMES		*P* ≤ 2e-16	yes
GNLY		*P* = 0.64	no
IFIT3		*P* = 0.0013	yes
HOPX		*P* = 0.00019	yes
KLRG1		*P* = 0.009	yes
KLRD1		*P* = 0.002	yes
NKG7		*P* = 2.8e-12	yes
GZMK		*P* = 0.65	no
GZMB		*P* = 0.24	no
GZMA		*P* = 0.0016	yes
PFN1		*P* = 0.43	no
IL10		*P* = 0.54	no
IL9		*P* = 0.5	no
SPI1		*P* = 0.33	no
IL1RL1		*P* = 0.41	no
IL13		*P* = 0.56	no
IL5		*P* = 0.5	no
IL4		*P* = 0.36	no
PLAC8		*P* = 0.035	yes
IGFBP7		*P* = 0.24	no
CCR4		*P* = 0.017	yes
ILF2		*P* = 0.26	no
GATA3		*P* = 0.1	no
TGFB1		*P* = 0.48	no
RORC		*P* = 0.33	no
IL1B		N.A.	N.A.
IL17A		N.A.	N.A.
IL22		*P* = 0.51	no
IL23A		*P* = 0.093	no
IL6ST		*P* = 0.002	yes
NETO2		*P* = 0.0014	yes
IFNG		*P* = 3.2e-8	yes
XCL1		*P* = 0.19	no
LTA		*P* = 0.096	no
IRF7		*P* = 0.00054	yes
STAT3		*P* = 0.031	yes
STAT1		*P* ≤ 2e-16	yes
CCL4		*P* ≤ 2e-16	yes
CCL3		*P* = 0.00021	yes
CCL5		*P* ≤ 2e-16	yes
CXCR3		*P* = 3.7e-14	yes
TBX21		*P* = 0.74	no

N.A. = not available

[grey box] significantly upregulated in PNS-myelin reactive vs. Flu reactive

Supplementary Table 2

PNS-myelin reactive T cell clones isolated from GBS patients. Detailed information of clonotype, CDR3β length, HLA restriction, epitope targeted, TCRβ publicity and GLIPH2 cluster contribution of all single T cell clones (n = 987, $CD4^+$ T cells; n = 55, $CD8^+$ T cells) obtained from the GBS patients included in the study.

PT	Clone number	Clone ID	Clonotype ID	Source	Specificity	CDR3β (aa)	TRBV	CDR3β length (nt)	in CSF	No Ag (cpm)	PNS-myelin antigen (cpm)	Epitope (aa)	Restriction	Public Clonotype	GLIPH CLUSTER (ID)
PT1-AC	1	D04AC	CD4_1	Blood	P0	CASSPTFVTGNTGELFF	TRBV5-4	45		171	2744	131-145	HLA-DR	no	
PT1-REC	2	C05REC	CD4_2	Blood	P0	CARRIVGDTQYF	TRBV7-7	39		109	1810	191-205	HLA-DR	no	
PT1-REC	3	H04REC	CD4_3	Blood	P0	CASSLARLFTGELFF	TRBV5-1	39		199	5098	191-205	HLA-DR	no	
PT1-REC	4	C12REC	CD4_4	Blood	P0	CASSLGGAGEQYF	TRBV7-8	33		357	1996	N.D.	N.D.	no	
PT1-REC	5	D06REC	CD4_5	Blood	P0	CASSLGMGRNQPQHF	TRBV5-8	39		143	1586	191-205	HLA-DR	no	
PT1-REC	6	A06REC	CD4_5	Blood	P0	CASSLGMGRNQPQHF	TRBV5-8	39		161	1529	191-205	HLA-DR	no	
PT1-REC	7	A07REC	CD4_5	Blood	P0	CASSLGMGRNQPQHF	TRBV5-8	39		270	1748	191-205	HLA-DR	no	
PT1-REC	8	A08REC	CD4_5	Blood	P0	CASSLGMGRNQPQHF	TRBV5-8	39		129	5070	191-205	HLA-DR	no	
PT1-REC	9	A12REC	CD4_5	Blood	P0	CASSLGMGRNQPQHF	TRBV5-8	39		157	3778	191-205	HLA-DR	no	
PT1-REC	10	B01REC	CD4_5	Blood	P0	CASSLGMGRNQPQHF	TRBV5-8	39		218	1804	191-205	HLA-DR	no	
PT1-REC	11	C03REC	CD4_5	Blood	P0	CASSLGMGRNQPQHF	TRBV5-8	39		140	6446	191-205	HLA-DR	no	
PT1-REC	12	C04REC	CD4_5	Blood	P0	CASSLGMGRNQPQHF	TRBV5-8	39		133	5871	191-205	HLA-DR	no	
PT1-REC	13	C07REC	CD4_5	Blood	P0	CASSLGMGRNQPQHF	TRBV5-8	39		81	1836	191-205	HLA-DR	no	
PT1-REC	14	C11REC	CD4_5	Blood	P0	CASSLGMGRNQPQHF	TRBV5-8	39		102	6486	191-205	HLA-DR	no	
PT1-REC	15	D02REC	CD4_5	Blood	P0	CASSLGMGRNQPQHF	TRBV5-8	39		166	1701	191-205	HLA-DR	no	
PT1-REC	16	D07REC	CD4_5	Blood	P0	CASSLGMGRNQPQHF	TRBV5-8	39		96	1927	191-205	HLA-DR	no	
PT1-REC	17	D08REC	CD4_5	Blood	P0	CASSLGMGRNQPQHF	TRBV5-8	39		180	4980	191-205	HLA-DR	no	
PT1-REC	18	D11REC	CD4_5	Blood	P0	CASSLGMGRNQPQHF	TRBV5-8	39		151	3473	191-205	HLA-DR	no	
PT1-REC	19	E02REC	CD4_5	Blood	P0	CASSLGMGRNQPQHF	TRBV5-8	39		275	3269	191-205	HLA-DR	no	
PT1-REC	20	E03REC	CD4_5	Blood	P0	CASSLGMGRNQPQHF	TRBV5-8	39		146	4246	191-205	HLA-DR	no	
PT1-REC	21	E06REC	CD4_5	Blood	P0	CASSLGMGRNQPQHF	TRBV5-8	39		110	4122	191-205	HLA-DR	no	
PT1-REC	22	E10REC	CD4_5	Blood	P0	CASSLGMGRNQPQHF	TRBV5-8	39		122	1198	191-205	HLA-DR	no	
PT1-REC	23	E12REC	CD4_5	Blood	P0	CASSLGMGRNQPQHF	TRBV5-8	39		134	3721	191-205	HLA-DR	no	
PT1-REC	24	F05REC	CD4_5	Blood	P0	CASSLGMGRNQPQHF	TRBV5-8	39		114	2038	191-205	HLA-DR	no	
PT1-REC	25	F09REC	CD4_5	Blood	P0	CASSLGMGRNQPQHF	TRBV5-8	39		152	4964	191-205	HLA-DR	no	
PT1-REC	26	G02REC	CD4_5	Blood	P0	CASSLGMGRNQPQHF	TRBV5-8	39		166	6967	191-205	HLA-DR	no	
PT1-REC	27	G04REC	CD4_5	Blood	P0	CASSLGMGRNQPQHF	TRBV5-8	39		184	6909	191-205	HLA-DR	no	
PT1-REC	28	G05REC	CD4_5	Blood	P0	CASSLGMGRNQPQHF	TRBV5-8	39		168	5739	191-205	HLA-DR	no	
PT1-REC	29	G12REC	CD4_5	Blood	P0	CASSLGMGRNQPQHF	TRBV5-8	39		99	5631	191-205	HLA-DR	no	
PT1-REC	30	H03REC	CD4_6	Blood	P0	CASSQGARDTQYF	TRBV11-2	33		161	1651	N.D.	HLA-DR	no	
PT1-REC	31	F01REC	CD4_6	Blood	P0	CASSQGARDTQYF	TRBV11-2	33		223	1029	26-40	HLA-DR	no	
PT1-REC	32	F02REC	CD4_6	Blood	P0	CASSQGARDTQYF	TRBV11-2	33		267	1029	26-40	HLA-DR	no	
PT1-REC	33	C02REC	CD4_7	Blood	P0	CASSSGTASTDTQYF	TRBV7-7	39		62	1155	141-155	HLA-DR	no	
PT1-REC	34	H07REC	N.D.	Blood	P0	N.D.	N.D.	N.D.		76	5117	141-155	N.D.	N.D.	
PT1-AC	35	D07AC	N.D.	Blood	P0	N.D.	N.D.	N.D.		471	1085	N.D.	N.D.	N.D.	
PT1-AC	36	F06AC	N.D.	Blood	P0	N.D.	N.D.	N.D.		359	1125	N.D.	N.D.	N.D.	
PT1-AC	37	G02AC	N.D.	Blood	P0	N.D.	N.D.	N.D.		468	1373	N.D.	N.D.	N.D.	
PT1-REC	38	G10REC	N.D.	Blood	P0	N.D.	N.D.	N.D.		183	3535	191-205	HLA-DR	N.D.	
PT1-REC	39	E11REC	N.D.	Blood	P0	N.D.	N.D.	N.D.		139	4671	N.D.	HLA-DR	N.D.	
PT2-AC	40	A04AC	CD4_8	Blood	P0	CASREGANSPLHF	TRBV19	33		2229	35270	21-35	N.D.	no	
PT2-REC	41	F07REC3	CD4_9	Blood	P0-CMV	CASSERGGANTGELFF	TRBV6-1	48		178	1907	N.D.	HLA-DR/DQ	no	
PT2-REC	42	A09REC3	CD4_9	Blood	P0	CASSERGGANTGELFF	TRBV6-2	48		135	1234	N.D.	N.D.	no	
PT2-REC	43	B12REC3	CD4_10	Blood	P0	CASSPISAQGAFTEAFF	TRBV27	51		123	1461	N.D.	N.D.	no	
PT2-REC	44	D03REC3	CD4_11	Blood	P0	CASTSGQKNTEAFF	TRBV6-2	42		127	1230	N.D.	HLA-DR	no	
PT2-REC	45	E12REC3	CD4_12	Blood	P0-CMV	CAWSKEGAEAFF	TRBV30	36		141	1488	N.D.	N.D.	no	
PT2-REC	46	E02REC3	CD4_13	Blood	P0-CMV	CAWSMQYEQYF	TRBV30	33		152	1743	N.D.	HLA-DR	no	CLUSTER_9
PT2-REC	47	A06REC3	N.D.	Blood	P0-CMV	N.D.	N.D.	N.D.		196	1547	N.D.	HLA-DR	N.D.	
PT2-REC	48	B05REC3	N.D.	Blood	P0-CMV	N.D.	N.D.	N.D.		130	1739	N.D.	HLA-DR	N.D.	
PT2-REC	49	E11REC3	N.D.	Blood	P0-CMV	N.D.	N.D.	N.D.		102	1887	N.D.	N.D.	N.D.	
PT2-REC	50	H06REC1	CD4_14	Blood	P0-CMV	CASSPAGTRINEQFF	TRBV19	45		118	1195	N.D.	HLA-DR	no	
PT2-REC	51	C06REC1	CD4_15	Blood	P0	CASSQEEAGGMDEQFF	TRBV4-1	48		148	1587	N.D.	N.D.	no	
PT2-REC	52	G05REC1	CD4_16	Blood	P0	CASSYPRTGGRDEQYF	TRBV6-2	48		159	1734	N.D.	N.D.	no	
PT2-REC	53	C12REC1	CD4_17	Blood	P0	CASSYSTYRGPRE#EQYF	TRBV6-6	54		205	2077	61-85	HLA-DR	no	
PT2-REC	54	H12REC1	CD4_18	Blood	P0	CASSYVRGEYGYTF	TRBV6-5	42		153	1576	N.D.	HLA-DR	no	
PT2-REC	55	D02REC1	CD4_19	Blood	P0	CATWGKGQLNTEAFF	TRBV27	45		457	1899	N.D.	N.D.	no	
PT2-REC	56	C11REC1	CD4_20	Blood	P0	CAWGGSGELFF	TRBV30	33		207	1947	N.D.	N.D.	no	
PT2-REC	57	C08REC1	CD4_21	Blood	P0-CMV	CAWGQGGNQPQHF	TRBV30	39		147	2699	N.D.	N.D.	no	CLUSTER_8
PT2-REC	58	B09REC1	N.D.	Blood	P0	N.D.	N.D.	N.D.		367	1655	N.D.	N.D.	N.D.	
PT2-REC	59	D04REC1	N.D.	Blood	P0-CMV	N.D.	N.D.	N.D.		868	2139	N.D.	HLA-DR	N.D.	
PT2-REC	60	A03REC1	N.D.	Blood	P0-CMV	N.D.	N.D.	N.D.		146	2331	N.D.	HLA-DR	N.D.	
PT2-REC	61	D01REC1	N.D.	Blood	P0-CMV	N.D.	N.D.	N.D.		798	3367	N.D.	N.D.	N.D.	
PT2-REC	62	G09REC1	N.D.	Blood	P0-CMV	N.D.	N.D.	N.D.		298	1387	N.D.	HLA-DR	N.D.	
PT3-REC	63	B08REC	CD4_22	Blood	P0	CASSPPARGQGYTF	TRBV18	36		406	9057	186-200	N.D.	no	
PT3-REC	64	D08REC	CD4_22	Blood	P0	CASSPPARGQGYTF	TRBV18	36		292	5100	186-200	HLA-DR	no	
PT3-REC	65	C08REC	CD4_22	Blood	P0	CASSPPARGQGYTF	TRBV18	36		256	6697	N.D.	N.D.	no	
PT3-REC	66	C10REC	CD4_22	Blood	P0	CASSPPARGQGYTF	TRBV18	36		290	5431	N.D.	N.D.	no	
PT3-REC	67	G08REC	CD4_22	Blood	P0	CASSPPARGQGYTF	TRBV18	36		302	2149	N.D.	N.D.	no	
PT4-REC	68	C01REC	CD4_23	Blood	P0	CASSVQPTNEKLFF	TRBV9	42		139	5506	N.D.	N.D.	no	
PT4-REC	69	B09REC	N.D.	Blood	P0	N.D.	N.D.	N.D.		71	1846	166-180	HLA-DR	N.D.	
PT7-REC	70	CL35REC	CD4_24	Blood	P0	CASNPRGGQYEQYF	TRBV7-8	36	in PT11	2471	6185	N.D.	N.D.	no	
PT7-REC	71	CL37REC	CD4_25	Blood	P0	CASSCYPGLQETQYF	TRBV7-2	39		1422	8484	46-60	N.D.	no	
PT7-REC	72	CL09REC	CD4_26	Blood	P0	CASSIGGPARGYTF	TRBV19	36		166	2403	N.D.	N.D.	no	
PT7-REC	73	CL12REC	CD4_27	Blood	P0	CASSLARGEQYF	TRBV5-1	30		1160	19869	21-35	HLA-DR	no	
PT7-REC	74	CL06REC	CD4_28	Blood	P0	CASSLRGDSGVRSTDTQYF	TRBV7-2	48	in PT11	436	7440	46-65	HLA-DP	yes	
PT7-REC	75	CL01REC	CD4_28	Blood	P0	CASSLRGDSGVRSTDTQYF	TRBV7-2	48	in PT11	176	3239	N.D.	N.D.	no	
PT7-REC	76	CL15REC	CD4_28	Blood	P0	CASSLRGDSGVRSTDTQYF	TRBV7-2	48	in PT11	823	3980	N.D.	HLA-DP	no	
PT7-REC	77	CL18REC	CD4_28	Blood	P0	CASSLRGDSGVRSTDTQYF	TRBV7-2	48	in PT11	372	5924	N.D.	HLA-DP	no	
PT7-REC	78	CL20REC	CD4_28	Blood	P0	CASSLRGDSGVRSTDTQYF	TRBV7-2	48	in PT11	158	8485	N.D.	HLA-DP	no	
PT7-REC	79	CL24REC	CD4_28	Blood	P0	CASSLRGDSGVRSTDTQYF	TRBV7-2	48	in PT11	635	2677	N.D.	HLA-DP	no	
PT7-REC	80	CL32REC	CD4_28	Blood	P0	CASSLRGDSGVRSTDTQYF	TRBV7-2	48	in PT11	127	4419	N.D.	HLA-DP	no	
PT7-REC	81	CL34REC	CD4_28	Blood	P0	CASSLRGDSGVRSTDTQYF	TRBV7-2	48	in PT11	370	1974	N.D.	HLA-DP	no	
PT7-REC	82	CL37REC	CD4_28	Blood	P0	CASSLRGDSGVRSTDTQYF	TRBV7-2	48	in PT11	192	3752	N.D.	HLA-DP	no	
PT7-REC	83	CL52REC	CD4_28	Blood	P0	CASSLRGDSGVRSTDTQYF	TRBV7-2	48	in PT11	1741	5289	N.D.	HLA-DP	no	
PT7-REC	84	CL60REC	CD4_28	Blood	P0	CASSLRGDSGVRSTDTQYF	TRBV7-2	48	in PT11	333	4856	N.D.	HLA-DP	no	
PT7-REC	85	CL61REC	CD4_28	Blood	P0	CASSLRGDSGVRSTDTQYF	TRBV7-2	48	in PT11	3957	10043	N.D.	HLA-DP	no	
PT7-REC	86	CL65REC	CD4_28	Blood	P0	CASSLRGDSGVRSTDTQYF	TRBV7-2	48	in PT11	155	1707	N.D.	N.D.	no	
PT7-REC	87	CL67REC	CD4_28	Blood	P0	CASSLRGDSGVRSTDTQYF	TRBV7-2	48	in PT11	891	3775	N.D.	HLA-DP	no	
PT7-REC	88	CL69REC	CD4_28	Blood	P0	CASSLRGDSGVRSTDTQYF	TRBV7-2	48	in PT11	630	2386	N.D.	HLA-DP	no	
PT7-REC	89	CL07REC	CD4_28	Blood	P0	CASSLRGDSGVRSTDTQYF	TRBV7-2	48	in PT11	377	3386	N.D.	HLA-DP	no	
PT7-REC	90	CL70REC	CD4_28	Blood	P0	CASSLRGDSGVRSTDTQYF	TRBV7-2	48	in PT11	864	3453	N.D.	HLA-DP	no	
PT7-REC	91	CL73REC	CD4_28	Blood	P0	CASSLRGDSGVRSTDTQYF	TRBV7-2	48	in PT11	122	4854	N.D.	HLA-DP	no	
PT7-REC	92	CL83REC	CD4_28	Blood	P0	CASSLRGDSGVRSTDTQYF	TRBV7-2	48	in PT11	651	4257	N.D.	HLA-DP	no	
PT7-REC	93	CL85REC	CD4_28	Blood	P0	CASSLRGDSGVRSTDTQYF	TRBV7-2	48	in PT11	162	5917	N.D.	HLA-DP	no	
PT7-REC	94	CL93REC	CD4_28	Blood	P0	CASSLRGDSGVRSTDTQYF	TRBV7-2	48	in PT11	2875	7103	N.D.	HLA-DP	no	
PT7-REC	95	CL96REC	CD4_28	Blood	P0	CASSLRGDSGVRSTDTQYF	TRBV7-2	48	in PT11	869	7747	N.D.	HLA-DP	no	
PT7-REC	96	CL97REC	CD4_28	Blood	P0	CASSLRGDSGVRSTDTQYF	TRBV7-2	48	in PT11	247	5027	N.D.	HLA-DP	no	
PT7-REC	97	CL71REC	CD4_28	Blood	P0	CASSLRGDSGVRSTDTQYF	TRBV7-2	48	in PT11	9	6207	46-65	HLA-DP	no	
PT7-REC	98	CL26REC	CD4_29	Blood	P0	CASSPALFGLAKNIQYF	TRBV7-9	45		1153	5042	N.D.	HLA-DP	no	
PT7-REC	99	CL25REC	CD4_30	Blood	P0	CASSPRAGHNPESGDMFF	TRBV7-2	48		162	2889	11-25	HLA-DR	no	
PT7-REC	100	CL28REC	CD4_31	Blood	P0	CASSPRAGHNPESGELFF	TRBV7-2	48		1258	5821	11-25	HLA-DR	no	
PT7-REC	101	CL46REC	CD4_32	Blood	P0	CASSSYPGLQETQYF	TRBV7-2	39		139	3398	46-60	HLA-DQ	no	
PT7-REC	102	CL13REC	CD4_32	Blood	P0	CASSSYPGLQETQYF	TRBV7-2	39		256	5933	46-60	N.D.	no	
PT7-REC	103	CL27REC	CD4_33	Blood	P0	CATSREGGGHLYNEQFF	TRBV15	45		2395	20756	76-90	N.D.	yes	
PT7-REC	104	CL23REC	CD4_34	Blood	P0	CSARVANSYNEQFF	TRBV20	36	in PT11	1677	10051	151-165	N.D.	yes	
PT7-REC	105	CL38REC	CD4_34	Blood	P0	CSARVANSYNEQFF	TRBV20	36	in PT11	2662	10645	N.D.	N.D.	no	
PT7-REC	106	CL39REC	CD4_34	Blood	P0	CSARVANSYNEQFF	TRBV20	36	in PT11	596	2198	N.D.	N.D.	no	
PT7-REC	107	CL55REC	CD4_34	Blood	P0	CSARVANSYNEQFF	TRBV20	36	in PT11	408	6536	N.D.	N.D.	no	
PT7-REC	108	CL47REC	CD4_34	Blood	P0	CSARVANSYNEQFF	TRBV20	36	in PT11	929	8998	N.D.	N.D.	no	
PT7-REC	109	CL48REC	CD4_35	Blood	P0	CSATRTSTETQYF	TRBV29	33		543	3661	21-35	HLA-DR	no	
PT7-REC	110	CL89REC	CD4_36	Blood	P0	CSVLGGYGELFF	TRBV29	36		2854	27653	21-35	HLA-DR	yes	
PT7-REC	111	CL31REC	N.D.	Blood	P0	N.D.	N.D.	N.D.		923	17558	21-35	HLA-DR	N.D.	
PT7-REC	112	CL100REC	N.D.	Blood	P0	N.D.	N.D.	N.D.		1980	13337	N.D.	N.D.	N.D.	
PT7-REC	113	CL102REC	N.D.	Blood	P0	N.D.	N.D.	N.D.		1234	5513	N.D.	N.D.	N.D.	
PT7-REC	114	CL16REC	N.D.	Blood	P0	N.D.	N.D.	N.D.		2125	6215	N.D.	N.D.	N.D.	
PT7-REC	115	CL22REC	N.D.	Blood	P0	N.D.	N.D.	N.D.		2180	15684	N.D.	N.D.	N.D.	
PT7-REC	116	CL25REC	N.D.	Blood	P0	N.D.	N.D.	N.D.		3211	14111	N.D.	N.D.	N.D.	
PT7-REC	117	CL30REC	N.D.	Blood	P0	N.D.	N.D.	N.D.		2451	15195	N.D.	N.D.	N.D.	
PT7-REC	118	CL35REC	N.D.	Blood	P0	N.D.	N.D.	N.D.		1803	16348	N.D.	N.D.	N.D.	
PT7-REC	119	CL36REC	N.D.	Blood	P0	N.D.	N.D.	N.D.		1596	18468	N.D.	N.D.	N.D.	
PT7-REC	120	CL50REC	N.D.	Blood	P0	N.D.	N.D.	N.D.		4575	19691	N.D.	N.D.	N.D.	
PT7-REC	121	CL81REC	N.D.	Blood	P0	N.D.	N.D.	N.D.		3045	24762	N.D.	N.D.	N.D.	
PT7-REC	122	CL99REC	N.D.	Blood	P0	N.D.	N.D.	N.D.		3262	19025	N.D.	N.D.	N.D.	
PT8-AC	123	A09AC	CD4_37	Blood	P0	CADHNQLGPGSSDLHF	TRBV7-8	42		872	5939	N.D.	HLA-DQ	no	
PT8-AC	124	B04AC	CD4_38	Blood	P0	CASNQPGQGRYEQYF	TRBV7-8	39		1849	10562	N.D.	HLA-DQ	no	
PT8-AC	125	G10AC	CD4_39	Blood	P0	CASNQPGQGSYEQYF	TRBV7-8	39		1026	7293	N.D.	HLA-DQ	yes	
PT8-AC	126	A02AC	CD4_39	Blood	P0	CASNQPGQGSYEQYF	TRBV7-8	39		1590	6751	N.D.	HLA-DQ	no	
PT8-AC	127	A03AC	CD4_39	Blood	P0	CASNQPGQGSYEQYF	TRBV7-8	39		1934	5316	N.D.	N.D.	no	
PT8-AC	128	C04AC	CD4_39	Blood	P0	CASNQPGQGSYEQYF	TRBV7-8	39		628	4902	N.D.	N.D.	no	

PT8-AC	129	C06AC	CD4_39	Blood	P0	CASNQPGQGSYEQYF	TRBV7-8	39		1766	5335	N.D.	N.D.	no	
PT8-AC	130	D01AC	CD4_39	Blood	P0	CASNQPGQGSYEQYF	TRBV7-8	39		987	8504	N.D.	N.D.	no	
PT8-AC	131	D02AC	CD4_39	Blood	P0	CASNQPGQGSYEQYF	TRBV7-8	39		635	10301	N.D.	N.D.	no	
PT8-AC	132	D05AC	CD4_39	Blood	P0	CASNQPGQGSYEQYF	TRBV7-8	39		981	4934	N.D.	N.D.	no	
PT8-AC	133	D08AC	CD4_39	Blood	P0	CASNQPGQGSYEQYF	TRBV7-8	39		1395	5196	N.D.	N.D.	no	
PT8-AC	134	E02AC	CD4_39	Blood	P0	CASNQPGQGSYEQYF	TRBV7-8	39		1983	10321	N.D.	N.D.	no	
PT8-AC	135	F03AC	CD4_39	Blood	P0	CASNQPGQGSYEQYF	TRBV7-8	39		1248	7071	N.D.	N.D.	no	
PT8-AC	136	G09AC	CD4_39	Blood	P0	CASNQPGQGSYEQYF	TRBV7-8	39		1704	7321	N.D.	N.D.	no	
PT8-AC	137	H03AC	CD4_39	Blood	P0	CASNQPGQGSYEQYF	TRBV7-8	39		938	4960	N.D.	N.D.	no	
PT8-AC	138	H04AC	CD4_39	Blood	P0	CASNQPGQGSYEQYF	TRBV7-8	39		932	6837	N.D.	N.D.	no	
PT8-AC	139	F09AC	CD4_39	Blood	P0	CASNQPGQGSYEQYF	TRBV7-8	39		785	6535	N.D.	N.D.	no	
PT8-AC	140	B03AC	CD4_39	Blood	P0	CASNQPGQGSYEQYF	TRBV7-8	39		620	5165	36-50	N.D.	no	
PT8-AC	141	F11AC	CD4_39	Blood	P0	CASNQPGQGSYEQYF	TRBV7-8	39		826	5433	36-50	N.D.	no	
PT8-AC	142	G01AC	CD4_39	Blood	P0	CASNQPGQGSYEQYF	TRBV7-8	39		492	6963	36-50	N.D.	no	
PT8-REC	143	D08REC	CD4_39	Blood	P0	CASNQPGQGSYEQYF	TRBV7-8	39		1297	8617	N.D.	HLA-DQ	no	
PT8-REC	144	C01REC	CD4_39	Blood	P0	CASNQPGQGSYEQYF	TRBV7-8	39		1497	7714	N.D.	HLA-DQ	no	
PT8-REC	145	C04REC	CD4_39	Blood	P0	CASNQPGQGSYEQYF	TRBV7-8	39		2486	8798	N.D.	N.D.	no	
PT8-REC	146	D05REC	CD4_39	Blood	P0	CASNQPGQGSYEQYF	TRBV7-8	39		2359	9770	N.D.	N.D.	no	
PT8-REC	147	B02REC	CD4_40	Blood	P0	CASNQPGQGSKEQYF	TRBV7-8	39		1715	6319	N.D.	HLADR/DQ	no	
PT8-REC	148	B06REC	CD4_40	Blood	P0	CASNQPGQGSKEQYF	TRBV7-8	39		510	2708	N.D.	N.D.	no	
PT8-AC	149	C10AC	N.D.	Blood	P0	N.D.	N.D.	N.D.		224	4963	N.D.	N.D.	N.D.	
PT8-AC	150	H09REC	N.D.	Blood	P0	N.D.	N.D.	N.D.		1336	5481	N.D.	N.D.	N.D.	
PT8-AC	151	G06AC	N.D.	Blood	P0	N.D.	N.D.	N.D.		474	6694	N.D.	N.D.	N.D.	
PT8-AC	152	E09AC	N.D.	Blood	P0	N.D.	N.D.	N.D.		922	7249	N.D.	N.D.	N.D.	
PT8-AC	153	E10AC	N.D.	Blood	P0	N.D.	N.D.	N.D.		306	1700	N.D.	N.D.	N.D.	
PT8-AC	154	E04AC	N.D.	Blood	P0	N.D.	N.D.	N.D.		1482	6224	N.D.	N.D.	N.D.	
PT8-AC	155	B02AC	N.D.	Blood	P0	N.D.	N.D.	N.D.		1521	4168	N.D.	N.D.	N.D.	
PT8-AC	156	A11AC	N.D.	Blood	P0	N.D.	N.D.	N.D.		85	2825	N.D.	N.D.	N.D.	
PT8-AC	157	A08AC	N.D.	Blood	P0	N.D.	N.D.	N.D.		293	1637	N.D.	N.D.	N.D.	
PT8-AC	158	A06AC	N.D.	Blood	P0	N.D.	N.D.	N.D.		1902	7643	N.D.	N.D.	N.D.	
PT8-REC	159	D02REC	N.D.	Blood	P0	N.D.	N.D.	N.D.		669	4959	N.D.	N.D.	N.D.	
PT8-REC	160	A06REC	N.D.	Blood	P0	N.D.	N.D.	N.D.		636	5348	N.D.	N.D.	N.D.	
PT8-REC	161	H06REC	N.D.	Blood	P0	N.D.	N.D.	N.D.		1747	6302	N.D.	N.D.	N.D.	
PT8-REC	162	A05REC	N.D.	Blood	P0	N.D.	N.D.	N.D.		190	2212	N.D.	N.D.	N.D.	
PT8-REC	163	A10REC	N.D.	Blood	P0	N.D.	N.D.	N.D.		1171	8175	N.D.	N.D.	N.D.	
PT8-REC	164	B03REC	N.D.	Blood	P0	N.D.	N.D.	N.D.		952	2501	N.D.	N.D.	N.D.	
PT8-REC	165	C03REC	N.D.	Blood	P0	N.D.	N.D.	N.D.		271	1213	N.D.	N.D.	N.D.	
PT8-REC	166	C10REC	N.D.	Blood	P0	N.D.	N.D.	N.D.		83	1850	N.D.	N.D.	N.D.	
PT8-REC	167	D04REC	N.D.	Blood	P0	N.D.	N.D.	N.D.		258	1267	N.D.	N.D.	N.D.	
PT8-REC	168	E02REC	N.D.	Blood	P0	N.D.	N.D.	N.D.		524	3565	N.D.	N.D.	N.D.	
PT8-REC	169	C05REC	N.D.	Blood	P0	N.D.	N.D.	N.D.		1404	6166	N.D.	N.D.	N.D.	
PT10-AC	170	F11AC	CD4_41	Blood	P0	CAHKPVGGTEAFF	TRBV6-2	33		135	3843	191-205	HLA-DR	no	
PT10-AC	171	E12AC	CD4_42	Blood	P0	CANQTGGYEQYF	TRBV6-5	30	in PT11	94	13000	131-150	N.D.	yes	CLUSTER_7
PT10-AC	172	B08AC	CD4_42	Blood	P0	CANQTGGYEQYF	TRBV6-5	30	in PT11	81	3344	N.D.	N.D.	yes	CLUSTER_7
PT10-AC	173	C03AC	CD4_42	Blood	P0	CANQTGGYEQYF	TRBV6-5	30	in PT11	81	2826	N.D.	N.D.	yes	CLUSTER_7
PT10-AC	174	C10AC	CD4_42	Blood	P0	CANQTGGYEQYF	TRBV6-5	30	in PT11	120	2342	N.D.	N.D.	yes	CLUSTER_7
PT10-AC	175	E10AC	CD4_42	Blood	P0	CANQTGGYEQYF	TRBV6-5	30	in PT11	99	2053	N.D.	N.D.	yes	CLUSTER_7
PT10-AC	176	E11AC	CD4_42	Blood	P0	CANQTGGYEQYF	TRBV6-5	30	in PT11	111	3788	N.D.	N.D.	yes	CLUSTER_7
PT10-AC	177	F02AC	CD4_42	Blood	P0	CANQTGGYEQYF	TRBV6-5	30	in PT11	95	7298	N.D.	N.D.	yes	CLUSTER_7
PT10-AC	178	F10AC	CD4_42	Blood	P0	CANQTGGYEQYF	TRBV6-5	30	in PT11	126	15044	N.D.	N.D.	yes	CLUSTER_7
PT10-AC	179	G03AC	CD4_42	Blood	P0	CANQTGGYEQYF	TRBV6-5	30	in PT11	132	7033	N.D.	N.D.	yes	CLUSTER_7
PT10-AC	180	H03AC	CD4_42	Blood	P0	CANQTGGYEQYF	TRBV6-5	30	in PT11	97	6915	N.D.	N.D.	yes	CLUSTER_7
PT10-AC	181	H06AC	CD4_42	Blood	P0	CANQTGGYEQYF	TRBV6-5	30	in PT11	142	2624	N.D.	N.D.	yes	CLUSTER_7
PT10-AC	182	F07AC	CD4_43	Blood	P0	CASTEQWTYSGNTIYF	TRBV19	42		162	2756	146-160	HLA-DR	no	
PT10-AC	183	H01AC	CD4_43	Blood	P0	CASTEQWTYSGNTIYF	TRBV19	42		115	2308	146-160	N.D.	no	
PT10-AC	184	A07AC	N.D.	Blood	P0	N.D.	N.D.	N.D.		155	5966	N.D.	N.D.	N.D.	
PT10-AC	185	B03AC	N.D.	Blood	P0	N.D.	N.D.	N.D.		103	3312	N.D.	N.D.	N.D.	
PT10-AC	186	B10AC	N.D.	Blood	P0	N.D.	N.D.	N.D.		99	2550	N.D.	N.D.	N.D.	
PT10-AC	187	B11AC	N.D.	Blood	P0	N.D.	N.D.	N.D.		120	1762	N.D.	N.D.	N.D.	
PT10-AC	188	C02AC	N.D.	Blood	P0	N.D.	N.D.	N.D.		233	2105	N.D.	N.D.	N.D.	
PT10-AC	189	C05AC	N.D.	Blood	P0	N.D.	N.D.	N.D.		172	2001	N.D.	N.D.	N.D.	
PT10-AC	190	C06AC	N.D.	Blood	P0	N.D.	N.D.	N.D.		106	1742	N.D.	N.D.	N.D.	
PT10-AC	191	C07AC	N.D.	Blood	P0	N.D.	N.D.	N.D.		89	4352	N.D.	N.D.	N.D.	
PT10-AC	192	C08AC	N.D.	Blood	P0	N.D.	N.D.	N.D.		138	3292	N.D.	N.D.	N.D.	
PT10-AC	193	C12AC	N.D.	Blood	P0	N.D.	N.D.	N.D.		185	2388	N.D.	N.D.	N.D.	
PT10-AC	194	D01AC	N.D.	Blood	P0	N.D.	N.D.	N.D.		136	3378	N.D.	N.D.	N.D.	
PT10-AC	195	D03AC	N.D.	Blood	P0	N.D.	N.D.	N.D.		145	4357	N.D.	N.D.	N.D.	
PT10-AC	196	D08AC	N.D.	Blood	P0	N.D.	N.D.	N.D.		75	5594	N.D.	N.D.	N.D.	
PT10-AC	197	D09AC	N.D.	Blood	P0	N.D.	N.D.	N.D.		94	5906	N.D.	N.D.	N.D.	
PT10-AC	198	D10AC	N.D.	Blood	P0	N.D.	N.D.	N.D.		121	1906	N.D.	N.D.	N.D.	
PT10-AC	199	D11AC	N.D.	Blood	P0	N.D.	N.D.	N.D.		186	2488	N.D.	N.D.	N.D.	
PT10-AC	200	E02AC	N.D.	Blood	P0	N.D.	N.D.	N.D.		109	8832	N.D.	N.D.	N.D.	
PT10-AC	201	E04AC	N.D.	Blood	P0	N.D.	N.D.	N.D.		185	1953	N.D.	N.D.	N.D.	
PT10-AC	202	E06AC	N.D.	Blood	P0	N.D.	N.D.	N.D.		108	2527	N.D.	N.D.	N.D.	
PT10-AC	203	E07AC	N.D.	Blood	P0	N.D.	N.D.	N.D.		57	3827	N.D.	N.D.	N.D.	
PT10-AC	204	E08AC	N.D.	Blood	P0	N.D.	N.D.	N.D.		112	5591	N.D.	N.D.	N.D.	
PT10-AC	205	E09AC	N.D.	Blood	P0	N.D.	N.D.	N.D.		80	7430	N.D.	N.D.	N.D.	
PT10-AC	206	F03AC	N.D.	Blood	P0	N.D.	N.D.	N.D.		138	1661	N.D.	N.D.	N.D.	
PT10-AC	207	F08AC	N.D.	Blood	P0	N.D.	N.D.	N.D.		151	2274	N.D.	N.D.	N.D.	
PT10-AC	208	G01AC	N.D.	Blood	P0	N.D.	N.D.	N.D.		210	6454	N.D.	N.D.	N.D.	
PT10-AC	209	H09AC	N.D.	Blood	P0	N.D.	N.D.	N.D.		112	5528	N.D.	N.D.	N.D.	
PT12-AC	210	A01AC	CD4_44	Blood	P0	CASSQDLGGGNTIYF	TRBV4-3	45		142	4881	N.D.	HLA-DQ	no	
PT12-AC	211	C11AC	CD4_44	Blood	P0	CASSQDLGGGNTIYF	TRBV4-3	45		118	1164	N.D.	HLA-DQ	no	
PT12-AC	212	D03AC	CD4_44	Blood	P0	CASSQDLGGGNTIYF	TRBV4-3	45		136	1267	N.D.	HLA-DQ	no	
PT12-AC	213	H08AC	CD4_44	Blood	P0	CASSQDLGGGNTIYF	TRBV4-3	45		102	1009	N.D.	HLA-DQ	no	
PT12-AC	214	H09AC	CD4_44	Blood	P0	CASSQDLGGGNTIYF	TRBV4-3	45		46	1243	N.D.	HLA-DQ	no	
PT12-REC	215	C04REC	CD4_45	Blood	P0	CASTPGLTYEQYF	TRBV2	39		106	1014	191-205 / 231-244	HLA-DR	no	
PT12-REC	216	A08REC	CD4_45	Blood	P0	CASTPGLTYEQYF	TRBV2	39		277	1616	191-205 / 231-245	HLA-DR	no	
PT12-AC	217	E06AC	N.D.	Blood	P0	N.D.	N.D.	N.D.		230	2113	N.D.	HLA-DQ	N.D.	
PT12-AC	218	A04AC	N.D.	Blood	P0	N.D.	N.D.	N.D.		68	4672	N.D.	HLA-DQ	N.D.	
PT12-AC	219	F05AC	N.D.	Blood	P0	N.D.	N.D.	N.D.		170	1102	N.D.	HLA-DR	N.D.	
PT12-AC	220	G09AC	N.D.	Blood	P0	N.D.	N.D.	N.D.		124	3143	N.D.	HLA-DP	N.D.	
PT13-AC	221	A01AC	CD4_46	Blood	P0	CSARDLVGSGYTF	TRBV20	39		291	9058	191-205	HLA-DR	no	
PT13-AC	222	B02AC	N.D.	Blood	P0	N.D.	N.D.	N.D.		256	6460	191-205	HLA-DR	N.D.	
PT14-AC	223	B05AC	CD4_47	Blood	P0	CASSLAGGVPNEKLFF	TRBV7-6	48		566	4068	N.D.	N.D.	no	
PT14-AC	224	C05AC	CD4_47	Blood	P0	CASSLAGGVPNEKLFF	TRBV7-6	48		739	3950	N.D.	N.D.	no	
PT14-AC	225	F04AC	CD4_47	Blood	P0	CASSLAGGVPNEKLFF	TRBV7-6	48		1799	7056	N.D.	N.D.	no	
PT14-AC	226	G06AC	CD4_47	Blood	P0	CASSLAGGVPNEKLFF	TRBV7-6	48		954	4608	N.D.	N.D.	no	
PT14-AC	227	H04AC	CD4_47	Blood	P0	CASSLAGGVPNEKLFF	TRBV7-6	48		778	2867	N.D.	N.D.	no	
PT14-AC	228	H06AC	CD4_47	Blood	P0	CASSLAGGVPNEKLFF	TRBV7-6	48		1461	4816	N.D.	N.D.	no	
PT14-AC	229	B01AC	CD4_48	Blood	P0	CASSPTGLWETQYF	TRBV6-1	42		129	4367	21-35	HLA-DR	no	
PT14-AC	230	B11AC	CD4_48	Blood	P0	CASSPTGLWETQYF	TRBV6-1	42		106	1446	N.D.	N.D.	no	
PT14-AC	231	D12AC	CD4_48	Blood	P0	CASSPTGLWETQYF	TRBV6-1	42		116	1077	N.D.	N.D.	no	
PT14-AC	232	E10AC	CD4_48	Blood	P0	CASSPTGLWETQYF	TRBV6-1	42		118	1693	N.D.	N.D.	no	
PT14-AC	233	G03AC	CD4_48	Blood	P0	CASSPTGLWETQYF	TRBV6-1	42		82	1053	N.D.	N.D.	no	
PT14-AC	234	E02AC	CD4_48	Blood	P0	CASSPTGLWETQYF	TRBV6-1	42		163	5148	N.D.	N.D.	no	
PT14-AC	235	H01AC	CD4_48	Blood	P0	CASSPTGLWETQYF	TRBV6-1	42		80	1554	N.D.	N.D.	no	
PT14-REC	236	A04REC	CD4_48	Blood	P0	CASSPTGLWETQYF	TRBV6-1	42		433	2434	21-35	N.D.	no	
PT14-REC	237	A06REC	CD4_48	Blood	P0	CASSPTGLWETQYF	TRBV6-1	42		384	7145	21-35	HLA-DR	no	
PT14-REC	238	E01REC	CD4_48	Blood	P0	CASSPTGLWETQYF	TRBV6-1	42		513	4901	N.D.	N.D.	no	
PT14-REC	239	E03REC	CD4_48	Blood	P0	CASSPTGLWETQYF	TRBV6-1	42		372	2116	N.D.	N.D.	no	
PT14-REC	240	F04REC	CD4_48	Blood	P0	CASSPTGLWETQYF	TRBV6-1	42		377	2855	N.D.	N.D.	no	
PT14-REC	241	F06REC	CD4_48	Blood	P0	CASSPTGLWETQYF	TRBV6-1	42		400	3563	N.D.	N.D.	no	
PT14-REC	242	G01REC	CD4_48	Blood	P0	CASSPTGLWETQYF	TRBV6-1	42		488	1701	N.D.	N.D.	no	
PT14-REC	243	H03REC	CD4_48	Blood	P0	CASSPTGLWETQYF	TRBV6-1	42		363	1689	N.D.	N.D.	no	
PT14-AC	244	A03AC	CD4_49	Blood	P0	CASTRTSGFEQFF	TRBV7-9	39		182	2502	N.D.	N.D.	no	
PT14-AC	245	A04AC	CD4_50	Blood	P0	CSATPAGGNTGELFF	TRBV20	45		182	4130	N.D.	HLA-DR	no	
PT14-AC	246	A05AC	CD4_50	Blood	P0	CSATPAGGNTGELFF	TRBV20	45		443	2944	N.D.	N.D.	no	
PT14-AC	247	A08AC	CD4_50	Blood	P0	CSATPAGGNTGELFF	TRBV20	45		347	2524	N.D.	N.D.	no	
PT14-AC	248	B03AC	CD4_50	Blood	P0	CSATPAGGNTGELFF	TRBV20	45		433	12288	N.D.	HLA-DR	no	
PT14-AC	249	B08AC	CD4_50	Blood	P0	CSATPAGGNTGELFF	TRBV20	45		337	2096	N.D.	N.D.	no	
PT14-AC	250	C06AC	CD4_50	Blood	P0	CSATPAGGNTGELFF	TRBV20	45		145	3366	N.D.	N.D.	no	
PT14-AC	251	D03AC	CD4_50	Blood	P0	CSATPAGGNTGELFF	TRBV20	45		793	6889	N.D.	N.D.	no	
PT14-AC	252	D08AC	CD4_50	Blood	P0	CSATPAGGNTGELFF	TRBV20	45		2765	15607	N.D.	N.D.	no	
PT14-AC	253	E03AC	CD4_50	Blood	P0	CSATPAGGNTGELFF	TRBV20	45		297	3088	N.D.	N.D.	no	
PT14-AC	254	E04AC	CD4_50	Blood	P0	CSATPAGGNTGELFF	TRBV20	45		171	2656	N.D.	HLA-DR	no	
PT14-AC	255	F05AC	CD4_50	Blood	P0	CSATPAGGNTGELFF	TRBV20	45		157	2169	N.D.	N.D.	no	
PT14-AC	256	F07AC	CD4_50	Blood	P0	CSATPAGGNTGELFF	TRBV20	45		146	2774	N.D.	N.D.	no	
PT14-AC	257	G03AC	CD4_50	Blood	P0	CSATPAGGNTGELFF	TRBV20	45		196	3132	N.D.	HLA-DR	no	
PT14-AC	258	G08AC	CD4_50	Blood	P0	CSATPAGGNTGELFF	TRBV20	45		210	3150	N.D.	N.D.	no	

PT14-AC	259	H03AC	CD4_50	Blood	P0	CSATPAGGNTGELFF	TRBV20	45		284	4621	N.D.	N.D.	no	
PT14-AC	260	H08AC	CD4_50	Blood	P0	CSATPAGGNTGELFF	TRBV20	45		436	9232	N.D.	N.D.	no	
PT14-AC	261	C04AC	CD4_50	Blood	P0	CSATPAGGNTGELFF	TRBV20	45		603	8885	N.D.	N.D.	no	
PT14-AC	262	F03AC	CD4_50	Blood	P0	CSATPAGGNTGELFF	TRBV20	45		157	4695	N.D.	N.D.	no	
PT14-AC	263	F08AC	CD4_50	Blood	P0	CSATPAGGNTGELFF	TRBV20	45		205	14490	N.D.	N.D.	no	
PT14-AC	264	H07AC	CD4_50	Blood	P0	CSATPAGGNTGELFF	TRBV20	45		190	2845	N.D.	N.D.	no	
PT14-AC	265	E08AC	CD4_50	Blood	P0	CSATPAGGNTGELFF	TRBV20	45		196	3610	N.D.	N.D.	no	
PT14-AC	266	E07AC	CD4_51	Blood	P0	CASSQGGSRAQETQYF	TRBV19	48		123	6786	N.D.	N.D.	no	
PT14-REC	267	D01REC	CD4_51	Blood	P0	CASSQGGSRAQETQYF	TRBV19	48		454	5993	76-90	HLA-DR	no	
PT14-REC	268	D05REC	CD4_51	Blood	P0	CASSQGGSRAQETQYF	TRBV19	48		501	3315	N.D.	N.D.	no	
PT14-REC	269	A01REC	CD4_51	Blood	P0	CASSQGGSRAQETQYF	TRBV19	48		464	12705	N.D.	N.D.	no	
PT14-REC	270	A02REC	CD4_51	Blood	P0	CASSQGGSRAQETQYF	TRBV19	48		364	11680	N.D.	N.D.	no	
PT14-REC	271	A03REC	CD4_51	Blood	P0	CASSQGGSRAQETQYF	TRBV19	48		632	7906	N.D.	N.D.	no	
PT14-REC	272	A05REC	CD4_51	Blood	P0	CASSQGGSRAQETQYF	TRBV19	48		374	3868	N.D.	N.D.	no	
PT14-REC	273	B01REC	CD4_51	Blood	P0	CASSQGGSRAQETQYF	TRBV19	48		410	6765	N.D.	N.D.	no	
PT14-REC	274	B02REC	CD4_51	Blood	P0	CASSQGGSRAQETQYF	TRBV19	48		452	4978	N.D.	N.D.	no	
PT14-REC	275	B03REC	CD4_51	Blood	P0	CASSQGGSRAQETQYF	TRBV19	48		322	8316	N.D.	N.D.	no	
PT14-REC	276	B04REC	CD4_51	Blood	P0	CASSQGGSRAQETQYF	TRBV19	48		510	2297	N.D.	N.D.	no	
PT14-REC	277	B05REC	CD4_51	Blood	P0	CASSQGGSRAQETQYF	TRBV19	48		345	8974	N.D.	N.D.	no	
PT14-REC	278	B06REC	CD4_51	Blood	P0	CASSQGGSRAQETQYF	TRBV19	48		488	6468	N.D.	N.D.	no	
PT14-REC	279	C01REC	CD4_51	Blood	P0	CASSQGGSRAQETQYF	TRBV19	48		496	7263	N.D.	N.D.	no	
PT14-REC	280	C02REC	CD4_51	Blood	P0	CASSQGGSRAQETQYF	TRBV19	48		452	7437	N.D.	N.D.	no	
PT14-REC	281	C04REC	CD4_51	Blood	P0	CASSQGGSRAQETQYF	TRBV19	48		392	11380	N.D.	N.D.	no	
PT14-REC	282	C05REC	CD4_51	Blood	P0	CASSQGGSRAQETQYF	TRBV19	48		374	11478	N.D.	N.D.	no	
PT14-REC	283	C06REC	CD4_51	Blood	P0	CASSQGGSRAQETQYF	TRBV19	48		549	5458	N.D.	N.D.	no	
PT14-REC	284	E01REC	CD4_51	Blood	P0	CASSQGGSRAQETQYF	TRBV19	48		513	4901	N.D.	N.D.	no	
PT14-REC	285	D02REC	CD4_51	Blood	P0	CASSQGGSRAQETQYF	TRBV19	48		363	7353	N.D.	N.D.	no	
PT14-REC	286	D03REC	CD4_51	Blood	P0	CASSQGGSRAQETQYF	TRBV19	48		472	2598	N.D.	N.D.	no	
PT14-REC	287	D05REC	CD4_51	Blood	P0	CASSQGGSRAQETQYF	TRBV19	48		494	7039	N.D.	N.D.	no	
PT14-REC	288	D06REC	CD4_51	Blood	P0	CASSQGGSRAQETQYF	TRBV19	48		323	8833	N.D.	N.D.	no	
PT14-REC	289	F01REC	CD4_51	Blood	P0	CASSQGGSRAQETQYF	TRBV19	48		408	8667	N.D.	N.D.	no	
PT14-REC	290	E04REC	CD4_51	Blood	P0	CASSQGGSRAQETQYF	TRBV19	48		443	2850	N.D.	N.D.	no	
PT14-REC	291	E05REC	CD4_51	Blood	P0	CASSQGGSRAQETQYF	TRBV19	48		311	5195	N.D.	N.D.	no	
PT14-REC	292	E06REC	CD4_51	Blood	P0	CASSQGGSRAQETQYF	TRBV19	48		368	9609	N.D.	N.D.	no	
PT14-REC	293	F03REC	CD4_51	Blood	P0	CASSQGGSRAQETQYF	TRBV19	48		456	10979	N.D.	N.D.	no	
PT14-REC	294	F05REC	CD4_51	Blood	P0	CASSQGGSRAQETQYF	TRBV19	48		307	8961	N.D.	N.D.	no	
PT14-REC	295	G02REC	CD4_51	Blood	P0	CASSQGGSRAQETQYF	TRBV19	48		276	11072	N.D.	N.D.	no	
PT14-REC	296	G03REC	CD4_51	Blood	P0	CASSQGGSRAQETQYF	TRBV19	48		288	12269	N.D.	N.D.	no	
PT14-REC	297	G04REC	CD4_51	Blood	P0	CASSQGGSRAQETQYF	TRBV19	48		474	4185	N.D.	N.D.	no	
PT14-REC	298	G05REC	CD4_51	Blood	P0	CASSQGGSRAQETQYF	TRBV19	48		588	11586	N.D.	N.D.	no	
PT14-REC	299	G06REC	CD4_51	Blood	P0	CASSQGGSRAQETQYF	TRBV19	48		494	1843	N.D.	N.D.	no	
PT14-REC	300	H01REC	CD4_51	Blood	P0	CASSQGGSRAQETQYF	TRBV19	48		573	7403	N.D.	N.D.	no	
PT14-REC	301	H02REC	CD4_51	Blood	P0	CASSQGGSRAQETQYF	TRBV19	48		472	5866	N.D.	N.D.	no	
PT14-REC	302	H04REC	CD4_51	Blood	P0	CASSQGGSRAQETQYF	TRBV19	48		608	5438	N.D.	N.D.	no	
PT14-REC	303	E12REC	CD4_52	Blood	P0	CASSLGGPNEQYF	TRBV7-8	39		254	2139	N.D.	N.D.	no	
PT14-REC	304	A10REC	CD4_53	Blood	P0	CASSPGGAEGTQYF	TRBV5-1	42		118	2137	N.D.	N.D.	no	
PT14-REC	305	A11REC	CD4_53	Blood	P0	CASSPGGAEGTQYF	TRBV5-1	42		125	4636	N.D.	N.D.	no	
PT14-REC	306	E10REC	CD4_53	Blood	P0	CASSPGGAEGTQYF	TRBV5-1	42		128	1763	N.D.	HLA-DR	no	
PT14-REC	307	E11REC	CD4_53	Blood	P0	CASSPGGAEGTQYF	TRBV5-1	42		75	2173	N.D.	N.D.	no	
PT14-REC	308	G10REC	CD4_53	Blood	P0	CASSPGGAEGTQYF	TRBV5-1	42		116	5063	N.D.	N.D.	no	
PT14-REC	309	G08REC	CD4_54	Blood	P0	CASSQDFGATVNTEAFF	TRBV4-2	51		109	1001	136-150	HLA-DP	no	
PT14-AC	310	C08AC	N.D.	Blood	P0	N.D.	N.D.	N.D.		423	10614	N.D.	N.D.	N.D.	
PT14-AC	311	D06AC	N.D.	Blood	P0	N.D.	N.D.	N.D.		151	7053	N.D.	N.D.	N.D.	
PT14-REC	312	H06REC	N.D.	Blood	P0	N.D.	N.D.	N.D.		462	3130	N.D.	N.D.	N.D.	
PT1-AC	313	E08AC	CD4_55	Blood	P2	CATGELGDFYNEQFF	TRBV15	39		812	25578	1-15	HLA-DR	no	
PT1-AC	314	D10AC	CD4_55	Blood	P2	CATGELGDFYNEQFF	TRBV15	39		678	14611	1-15	HLA-DR	no	
PT1-AC	315	F08AC	CD4_56	Blood	P2	CASDRAANYGYTF	TRBV30	33		499	1360	111-125	HLA-DR	no	
PT1-REC	316	B11REC	CD4_56	Blood	P2	CASDRAANYGYTF	TRBV30	33		110	1800	111-125	HLA-DR	no	
PT1-AC	317	D02AC	CD4_57	Blood	P2	CASMSSLWETQYF	TRBV2	33		182	3322	26-40	HLA-DR	no	
PT1-REC	318	F12REC	CD4_57	Blood	P2	CASMSSLWETQYF	TRBV2	33		135	1129	26-45	HLA-DR	no	
PT1-AC	319	A08AC	CD4_58	Blood	P2	CASSLGQGAGEQYF	TRBV5-1	36		186	3835	111-125	HLA-DR	no	
PT1-REC	320	H07REC	CD4_59	Blood	P2	CASSPVRATFNEQFF	TRBV7-3	39		179	11391	26-40	HLA-DR	yes	
PT1-AC	321	E09AC	CD4_59	Blood	P2	CASSPVRATFNEQFF	TRBV7-3	39		152	13781	26-40	HLA-DR	no	
PT1-AC	322	F01AC	CD4_59	Blood	P2	CASSPVRATFNEQFF	TRBV7-3	39		154	5178	26-40	HLA-DR	no	
PT1-AC	323	H08AC	CD4_60	Blood	P2	CASSQVGHGYTF	TRBV3	30		187	2393	N.D.	HLA-DR	no	
PT1-AC	324	A02AC	CD4_61	Blood	P2	CATSDLSMQPQHF	TRBV24	33		111	5200	56-70	HLA-DR	no	
PT1-AC	325	A03AC	CD4_61	Blood	P2	CATSDLSMQPQHF	TRBV24	33		197	1262	56-70	HLA-DR	no	
PT1-AC	326	H05AC	CD4_61	Blood	P2	CATSDLSMQPQHF	TRBV24	33		137	1559	56-70	HLA-DR	no	
PT1-REC	327	A10REC	CD4_62	Blood	P2	CAITTGSANYGYTF	TRBV10-3	36		126	2362	26-40	HLA-DR	no	
PT1-REC	328	D12REC	CD4_62	Blood	P2	CAITTGSANYGYTF	TRBV10-3	36		118	6658	26-40	HLA-DR	no	
PT1-REC	329	A05REC	CD4_62	Blood	P2	CAITTGSANYGYTF	TRBV10-3	36		310	9371	26-45	N.D.	no	
PT1-REC	330	A07REC	CD4_62	Blood	P2	CAITTGSANYGYTF	TRBV10-3	36		178	7547	26-45	HLA-DR	no	
PT1-REC	331	C08REC	CD4_62	Blood	P2	CAITTGSANYGYTF	TRBV10-3	36		121	3593	26-45	HLA-DR	no	
PT1-REC	332	D02REC	CD4_62	Blood	P2	CAITTGSANYGYTF	TRBV10-3	36		231	6459	26-45	HLA-DR	no	
PT1-REC	333	D05REC	CD4_62	Blood	P2	CAITTGSANYGYTF	TRBV10-3	36		261	7597	26-45	N.D.	no	
PT1-REC	334	D09REC	CD4_62	Blood	P2	CAITTGSANYGYTF	TRBV10-3	36		92	2430	26-45	HLA-DR	no	
PT1-REC	335	D10REC	CD4_62	Blood	P2	CAITTGSANYGYTF	TRBV10-3	36		367	6576	26-45	HLA-DR	no	
PT1-REC	336	E04REC	CD4_62	Blood	P2	CAITTGSANYGYTF	TRBV10-3	36		265	9307	26-45	N.D.	no	
PT1-REC	337	E07REC	CD4_62	Blood	P2	CAITTGSANYGYTF	TRBV10-3	36		148	4034	26-45	HLA-DR	no	
PT1-REC	338	E09REC	CD4_62	Blood	P2	CAITTGSANYGYTF	TRBV10-3	36		187	6345	26-45	HLA-DR	no	
PT1-REC	339	E12REC	CD4_62	Blood	P2	CAITTGSANYGYTF	TRBV10-3	36		126	4280	26-45	HLA-DR	no	
PT1-REC	340	F08REC	CD4_62	Blood	P2	CAITTGSANYGYTF	TRBV10-3	36		176	1069	26-45	HLA-DR	no	
PT1-REC	341	H03REC	CD4_62	Blood	P2	CAITTGSANYGYTF	TRBV10-3	36		206	8305	26-45	N.D.	no	
PT1-REC	342	A03REC	CD4_62	Blood	P2	CAITTGSANYGYTF	TRBV10-3	36		160	3190	26-45	HLA-DR	no	
PT1-REC	343	A06REC	CD4_63	Blood	P2	CASIRARPYEQYF	TRBV6-2	33		137	5203	81-95	HLA-DR	no	
PT1-REC	344	C10REC	CD4_64	Blood	P2	CASSLRMDTEAFF	TRBV5-1	33		177	2781	26-40	HLA-DR	no	
PT1-REC	345	D07REC	CD4_65	Blood	P2	CTSTLQGNTEAFF	TRBV5-1	33		112	2462	1-15	HLA-DR	no	
PT1-AC	346	A07AC	N.D.	Blood	P2	N.D.	N.D.	N.D.		186	9294	N.D.	HLA-DR	N.D.	
PT1-AC	347	C03AC	N.D.	Blood	P2	N.D.	N.D.	N.D.		201	6187	N.D.	HLA-DR	N.D.	
PT1-AC	348	H03AC	N.D.	Blood	P2	N.D.	N.D.	N.D.		488	4317	N.D.	N.D.	N.D.	
PT1-REC	349	C07REC	N.D.	Blood	P2	N.D.	N.D.	N.D.		89	2773	111-125	HLA-DR	N.D.	
PT1-REC	350	B12REC	N.D.	Blood	P2	N.D.	N.D.	N.D.		247	12761	26-40	HLA-DR	N.D.	
PT1-REC	351	C05REC	N.D.	Blood	P2	N.D.	N.D.	N.D.		133	11091	26-40	HLA-DR	N.D.	
PT1-REC	352	D03REC	N.D.	Blood	P2	N.D.	N.D.	N.D.		207	6970	26-40	HLA-DR	N.D.	
PT1-REC	353	G04REC	N.D.	Blood	P2	N.D.	N.D.	N.D.		207	8459	26-40	HLA-DR	N.D.	
PT1-REC	354	G08REC	N.D.	Blood	P2	N.D.	N.D.	N.D.		167	6422	26-40	HLA-DR	N.D.	
PT1-REC	355	G12REC	N.D.	Blood	P2	N.D.	N.D.	N.D.		103	5086	26-40	HLA-DR	N.D.	
PT1-REC	356	A02REC	N.D.	Blood	P2	N.D.	N.D.	N.D.		132	8050	N.D.	HLA-DR	N.D.	
PT1-REC	357	D11REC	N.D.	Blood	P2	N.D.	N.D.	N.D.		167	2808	N.D.	HLA-DR	N.D.	
PT1-REC	358	H05REC	N.D.	Blood	P2	N.D.	N.D.	N.D.		145	1321	N.D.	N.D.	N.D.	
PT1-REC	359	C01REC	N.D.	Blood	P2	N.D.	N.D.	N.D.		144	3893	36-50	HLA-DR	N.D.	
PT2-AC	360	B07AC	CD4_66	Blood	P2	CASSQVVGGTDTQYF	TRBV3	39		5259	7279	N.D.	N.D.	no	
PT2-AC	361	E06AC	CD4_66	Blood	P2	CASSQVVGGTDTQYF	TRBV3	39		3472	3894	N.D.	N.D.	no	
PT2-AC	362	H06AC	CD4_66	Blood	P2	CASSQVVGGTDTQYF	TRBV3	39		500	3212	N.D.	N.D.	no	
PT2-AC	363	C6AC	CD4_66	Blood	P2	CASSQVVGGTDTQYF	TRBV3	39		391	1751	1-15	HLA-DR	no	
PT2-AC	364	D05AC	CD4_66	Blood	P2	CASSQVVGGTDTQYF	TRBV3	39		448	2499	1-15	HLA-DR	no	
PT2-AC	365	G05AC	CD4_66	Blood	P2	CASSQVVGGTDTQYF	TRBV3	39		582	2509	1-15	HLA-DR	no	
PT2-AC	366	A06AC	CD4_66	Blood	P2	CASSQVVGGTDTQYF	TRBV3	39		640	1943	1-15	HLA-DR	no	
PT2-REC	367	E06REC	CD4_67	Blood	P2	CASSHPDRPDDTF	TRBV6-2	33		428	3351	76-90	HLA-DP	no	
PT2-REC	368	B04REC	CD4_68	Blood	P2	CASSIGHSTETQYF	TRBV19	36		459	5493	N.D.	N.D.	no	
PT2-REC	369	C04REC	CD4_68	Blood	P2	CASSIGHSTETQYF	TRBV19	36		322	5726	N.D.	N.D.	no	
PT2-REC	370	E09REC	CD4_68	Blood	P2	CASSIGHSTETQYF	TRBV19	36		303	3208	N.D.	N.D.	no	
PT2-REC	371	G06REC	CD4_68	Blood	P2	CASSIGHSTETQYF	TRBV19	36		422	5853	N.D.	N.D.	no	
PT2-REC	372	G11REC	CD4_68	Blood	P2	CASSIGHSTETQYF	TRBV19	36		429	4306	N.D.	N.D.	no	
PT2-REC	373	H05REC	CD4_68	Blood	P2	CASSIGHSTETQYF	TRBV19	36		418	4878	N.D.	N.D.	no	
PT2-REC	374	H06REC	CD4_68	Blood	P2	CASSIGHSTETQYF	TRBV19	36		410	7557	N.D.	N.D.	no	
PT2-REC	375	E01REC	CD4_68	Blood	P2	CASSIGHSTETQYF	TRBV19	36		486	4845	N.D.	N.D.	no	
PT2-REC	376	B06REC	CD4_68	Blood	P2	CASSIGHSTETQYF	TRBV19	36		310	6331	1-15	HLA-DR	no	
PT2-REC	377	D11REC	CD4_68	Blood	P2	CASSIGHSTETQYF	TRBV19	36		998	5315	1-15	HLA-DP	no	
PT2-REC	378	D12REC	CD4_68	Blood	P2	CASSIGHSTETQYF	TRBV19	36		452	4532	1-15	HLA-DP	no	
PT2-REC	379	C03REC	CD4_68	Blood	P2	CASSIGHSTETQYF	TRBV19	36		267	2564	1-15	HLA-DP	no	
PT2-REC	380	C07REC	CD4_69	Blood	P2	CASSLSANTGELFF	TRBV7-2	30		391	3342	N.D.	HLA-DR	no	
PT2-REC	381	G09REC	CD4_69	Blood	P2	CASSLSANTGELFF	TRBV7-2	30		371	1925	N.D.	HLA-DR	no	
PT2-REC	382	D09REC	CD4_69	Blood	P2	CASSLSANTGELFF	TRBV7-2	30		431	28718	N.D.	N.D.	no	
PT2-REC	383	F03REC	CD4_69	Blood	P2	CASSLSANTGELFF	TRBV7-2	30		818	16549	N.D.	N.D.	no	
PT2-REC	384	B02REC	CD4_69	Blood	P2	CASSLSANTGELFF	TRBV7-2	30		505	1356	1-15	HLA-DR	no	
PT2-REC	385	D01REC	CD4_69	Blood	P2	CASSLSANTGELFF	TRBV7-2	30		652	2471	1-15	HLA-DR	no	
PT2-REC	386	A12REC	CD4_69	Blood	P2	CASSLSANTGELFF	TRBV7-2	30		600	8856	1-15	HLA-DR	no	
PT2-REC	387	F12REC	CD4_70	Blood	P2	CASSLTAVSYEQYF	TRBV7-8	36		562	30496	1-15	HLA-DR	no	
PT2-REC	388	B01REC	CD4_70	Blood	P2	CASSLTAVSYEQYF	TRBV7-8	36		456	11981	1-15	HLA-DR	no	

PT2-REC	389	C08REC	CD4_71	Blood	P2	CASSPISAYNSPLHF	TRBV18	39		363	2785	N.D.	N.D.	no	
PT2-REC	390	H07REC	CD4_71	Blood	P2	CASSPISAYNSPLHF	TRBV18	39		379	13962	1-15	HLA-DR	no	
PT2-REC	391	H01REC	CD4_71	Blood	P2-CMV	CASSPISAYNSPLHF	TRBV18	39		650	19556	1-15	HLA-DR	no	
PT2-REC	392	A03REC	CD4_72	Blood	P2	CASSPQGNTIYF	TRBV5-1	30		871	15328	N.D.	N.D.	no	
PT2-REC	393	A01REC	CD4_72	Blood	P2	CASSPQGNTIYF	TRBV5-1	30		699	5040	N.D.	N.D.	no	
PT2-REC	394	A02REC	CD4_72	Blood	P2	CASSPQGNTIYF	TRBV5-1	30		567	7737	N.D.	N.D.	no	
PT2-REC	395	B03REC	CD4_72	Blood	P2	CASSPQGNTIYF	TRBV5-1	30		538	18905	N.D.	N.D.	no	
PT2-REC	396	B07REC	CD4_72	Blood	P2	CASSPQGNTIYF	TRBV5-1	30		533	9969	N.D.	N.D.	no	
PT2-REC	397	B11REC	CD4_72	Blood	P2	CASSPQGNTIYF	TRBV5-1	30		474	29565	N.D.	N.D.	no	
PT2-REC	398	C05REC	CD4_72	Blood	P2	CASSPQGNTIYF	TRBV5-1	30		463	14961	N.D.	N.D.	no	
PT2-REC	399	C09REC	CD4_72	Blood	P2	CASSPQGNTIYF	TRBV5-1	30		444	13040	N.D.	N.D.	no	
PT2-REC	400	C11REC	CD4_72	Blood	P2	CASSPQGNTIYF	TRBV5-1	30		341	5657	N.D.	N.D.	no	
PT2-REC	401	C02REC	CD4_72	Blood	P2	CASSPQGNTIYF	TRBV5-1	30		567	12816	N.D.	N.D.	no	
PT2-REC	402	D04REC	CD4_72	Blood	P2	CASSPQGNTIYF	TRBV5-1	30		359	10526	N.D.	N.D.	no	
PT2-REC	403	D05REC	CD4_72	Blood	P2	CASSPQGNTIYF	TRBV5-1	30		334	12666	N.D.	N.D.	no	
PT2-REC	404	D01REC	CD4_72	Blood	P2	CASSPQGNTIYF	TRBV5-1	30		652	2471	N.D.	N.D.	no	
PT2-REC	405	E05REC	CD4_72	Blood	P2	CASSPQGNTIYF	TRBV5-1	30		456	25295	N.D.	N.D.	no	
PT2-REC	406	E08REC	CD4_72	Blood	P2	CASSPQGNTIYF	TRBV5-1	30		303	19099	N.D.	N.D.	no	
PT2-REC	407	E11REC	CD4_72	Blood	P2	CASSPQGNTIYF	TRBV5-1	30		464	7012	N.D.	N.D.	no	
PT2-REC	408	E12REC	CD4_72	Blood	P2	CASSPQGNTIYF	TRBV5-1	30		485	10079	N.D.	N.D.	no	
PT2-REC	409	F11REC	CD4_72	Blood	P2	CASSPQGNTIYF	TRBV5-1	30		355	17372	N.D.	N.D.	no	
PT2-REC	410	F01REC	CD4_72	Blood	P2	CASSPQGNTIYF	TRBV5-1	30		826	30941	N.D.	N.D.	no	
PT2-REC	411	G02REC	CD4_72	Blood	P2	CASSPQGNTIYF	TRBV5-1	30		392	11407	N.D.	N.D.	no	
PT2-REC	412	G07REC	CD4_72	Blood	P2	CASSPQGNTIYF	TRBV5-1	30		466	16253	N.D.	N.D.	no	
PT2-REC	413	G10REC	CD4_72	Blood	P2	CASSPQGNTIYF	TRBV5-1	30		437	14066	N.D.	N.D.	no	
PT2-REC	414	H02REC	CD4_72	Blood	P2	CASSPQGNTIYF	TRBV5-1	30		541	15641	N.D.	N.D.	no	
PT2-REC	415	H04REC	CD4_72	Blood	P2	CASSPQGNTIYF	TRBV5-1	30		543	21745	N.D.	N.D.	no	
PT2-REC	416	H10REC	CD4_72	Blood	P2	CASSPQGNTIYF	TRBV5-1	30		656	24563	N.D.	N.D.	no	
PT2-REC	417	H11REC	CD4_72	Blood	P2	CASSPQGNTIYF	TRBV5-1	30		469	17240	N.D.	N.D.	no	
PT2-REC	418	H01REC	CD4_72	Blood	P2	CASSPQGNTIYF	TRBV5-1	30		488	24934	N.D.	N.D.	no	
PT2-REC	419	A10REC	CD4_72	Blood	P2	CASSPQGNTIYF	TRBV5-1	30		353	10744	1-15	HLA-DR	no	
PT2-REC	420	B08REC	CD4_72	Blood	P2	CASSPQGNTIYF	TRBV5-1	30		521	13561	1-15	HLA-DR	no	
PT2-REC	421	F02REC	CD4_72	Blood	P2	CASSPQGNTIYF	TRBV5-1	30		381	19349	1-15	HLA-DR	no	
PT2-REC	422	F04REC	CD4_72	Blood	P2	CASSPQGNTIYF	TRBV5-1	30		277	8103	1-15	HLA-DR	no	
PT2-REC	423	F09REC	CD4_72	Blood	P2	CASSPQGNTIYF	TRBV5-1	30		475	11271	1-15	HLA-DR	no	
PT2-REC	424	A11REC	CD4_72	Blood	P2	CASSPQGNTIYF	TRBV5-1	30		314	17697	1-15	HLA-DR	no	
PT2-REC	425	C06REC	CD4_73	Blood	P2	CASSQVGAYGYTF	TRBV3	33		587	32960	1-15	HLA-DR	no	
PT2-REC	426	D06REC	CD4_73	Blood	P2	CASSQVGAYGYTF	TRBV3	33		534	12931	1-15	HLA-DR	no	
PT2-REC	427	H09REC	CD4_73	Blood	P2	CASSQVGAYGYTF	TRBV3	33		566	6922	1-15	HLA-DR	no	
PT2-REC	428	A04REC	CD4_73	Blood	P2	CASSQVGAYGYTF	TRBV3	33		2229	35270	1-15	HLA-DR	no	
PT2-REC	429	A07REC	CD4_74	Blood	P2	CASSVKRGGQGNTEAFF	TRBV2	45		500	18145	N.D.	N.D.	no	
PT2-REC	430	A02REC	N.D.	Blood	P2	N.D.	N.D.	N.D.		567	7737	N.D.	N.D.	N.D.	
PT2-REC	431	A01REC	N.D.	Blood	P2	N.D.	N.D.	N.D.		699	5040	N.D.	N.D.	N.D.	
PT2-REC	432	A07REC	N.D.	Blood	P2	N.D.	N.D.	N.D.		500	18145	N.D.	N.D.	N.D.	
PT2-REC	433	B09REC	N.D.	Blood	P2	N.D.	N.D.	N.D.		423	4343	N.D.	N.D.	N.D.	
PT2-REC	434	B12REC	N.D.	Blood	P2	N.D.	N.D.	N.D.		427	13686	N.D.	N.D.	N.D.	
PT2-REC	435	E07REC	N.D.	Blood	P2	N.D.	N.D.	N.D.		519	6198	N.D.	N.D.	N.D.	
PT2-REC	436	F06REC	N.D.	Blood	P2	N.D.	N.D.	N.D.		334	21825	N.D.	N.D.	N.D.	
PT2-REC	437	G01REC	N.D.	Blood	P2	N.D.	N.D.	N.D.		452	6728	N.D.	N.D.	N.D.	
PT2-REC	438	A05REC	N.D.	Blood	P2	N.D.	N.D.	N.D.		636	20606	N.D.	N.D.	N.D.	
PT2-REC	439	D01REC3	CD4_75	Blood	P2-CMV	CAGRDSELSEQYF	TRBV30	39		384	3707	N.D.	N.D.	no	
PT2-REC	440	C05REC3	CD4_76	Blood	P2-CMV	CASSFGRAGSSYEQYF	TCRBV28	48		140	2447	N.D.	HLA-DR	no	
PT2-REC	441	D08REC3	CD4_77	Blood	P2	CAWRDGYNTEAFF	TRBV30	39		735	1263	N.D.	N.D.	no	
PT2-REC	442	D06REC3	CD4_77	Blood	P2-CMV	CAWRDGYNTEAFF	TRBV30	39		158	2082	N.D.	N.D.	no	
PT2-REC	443	D11REC1	CD4_77	Blood	P2	CAWRDGYNTEAFF	TRBV30	39		229	1467	N.D.	N.D.	no	
PT2-REC	444	A11REC3	CD4_78	Blood	P2-CMV	CAWSSGGVYEQYF	TRBV30	39		108	2777	N.D.	N.D.	no	
PT2-REC	445	A08REC3	CD4_79	Blood	P2-CMV	CAWSVPSTGNTEAFF	TRBV30	45		105	1949	N.D.	N.D.	no	
PT2-REC	446	A03REC3	N.D.	Blood	P2-CMV	N.D.	N.D.	N.D.		161	1197	N.D.	N.D.	N.D.	
PT2-REC	447	D02REC3	N.D.	Blood	P2-CMV	N.D.	N.D.	N.D.		100	3622	N.D.	N.D.	N.D.	
PT2-REC	448	D03REC3	N.D.	Blood	P2-CMV	N.D.	N.D.	N.D.		158	1350	N.D.	N.D.	N.D.	
PT2-REC	449	D04REC3	N.D.	Blood	P2-CMV	N.D.	N.D.	N.D.		156	1712	N.D.	N.D.	N.D.	
PT2-REC	450	D03REC1	N.D.	Blood	P2-CMV	N.D.	N.D.	N.D.		173	1500	N.D.	N.D.	N.D.	
PT2-REC	451	E04REC1	CD4_80	Blood	P2-CMV	CASSSLRTESSYNSPLHF	TRBV5-6	54		173	1347	N.D.	HLA-DR	no	
PT2-REC	452	F07REC1	CD4_19	Blood	P2	CATWGKGQLNTEAFF	TRBV27	45		126	1942	N.D.	N.D.	no	
PT2-REC	453	B06REC1	CD4_19	Blood	P2	CATWGKGQLNTEAFF	TRBV27	45		152	1964	N.D.	N.D.	no	
PT2-REC	454	E12REC1	N.D.	Blood	P2	N.D.	N.D.	N.D.		162	10335	N.D.	N.D.	N.D.	
PT3-REC	455	A03REC	CD4_81	Blood	P2	CASSLALGGFTEAFF	TRBV11-1	39		392	1047	N.D.	N.D.	no	
PT3-REC	456	A04REC	CD4_81	Blood	P2	CASSLALGGFTEAFF	TRBV11-1	39		321	1994	N.D.	N.D.	no	
PT3-REC	457	B01REC	CD4_81	Blood	P2	CASSLALGGFTEAFF	TRBV11-1	39		299	5937	N.D.	N.D.	no	
PT3-REC	458	B02REC	CD4_81	Blood	P2	CASSLALGGFTEAFF	TRBV11-1	39		336	2940	N.D.	N.D.	no	
PT3-REC	459	B05REC	CD4_81	Blood	P2	CASSLALGGFTEAFF	TRBV11-1	39		317	14468	N.D.	N.D.	no	
PT3-REC	460	B06REC	CD4_81	Blood	P2	CASSLALGGFTEAFF	TRBV11-1	39		281	8235	N.D.	N.D.	no	
PT3-REC	461	C01REC	CD4_81	Blood	P2	CASSLALGGFTEAFF	TRBV11-1	39		424	1617	N.D.	N.D.	no	
PT3-REC	462	C03REC	CD4_81	Blood	P2	CASSLALGGFTEAFF	TRBV11-1	39		449	3368	N.D.	N.D.	no	
PT3-REC	463	C05REC	CD4_81	Blood	P2	CASSLALGGFTEAFF	TRBV11-1	39		237	1484	N.D.	N.D.	no	
PT3-REC	464	C06REC	CD4_81	Blood	P2	CASSLALGGFTEAFF	TRBV11-1	39		384	8195	N.D.	N.D.	no	
PT3-REC	465	D01REC	CD4_81	Blood	P2	CASSLALGGFTEAFF	TRBV11-1	39		326	1148	N.D.	N.D.	no	
PT3-REC	466	D02REC	CD4_81	Blood	P2	CASSLALGGFTEAFF	TRBV11-1	39		346	8265	N.D.	N.D.	no	
PT3-REC	467	D04REC	CD4_81	Blood	P2	CASSLALGGFTEAFF	TRBV11-1	39		307	1022	N.D.	N.D.	no	
PT3-REC	468	D06REC	CD4_81	Blood	P2	CASSLALGGFTEAFF	TRBV11-1	39		283	6610	N.D.	N.D.	no	
PT3-REC	469	E04REC	CD4_81	Blood	P2	CASSLALGGFTEAFF	TRBV11-1	39		264	1034	N.D.	N.D.	no	
PT3-REC	470	F02REC	CD4_81	Blood	P2	CASSLALGGFTEAFF	TRBV11-1	39		338	7663	N.D.	N.D.	no	
PT3-REC	471	F03REC	CD4_81	Blood	P2	CASSLALGGFTEAFF	TRBV11-1	39		389	1787	N.D.	N.D.	no	
PT3-REC	472	F04REC	CD4_81	Blood	P2	CASSLALGGFTEAFF	TRBV11-1	39		363	2819	N.D.	N.D.	no	
PT3-REC	473	F05REC	CD4_81	Blood	P2	CASSLALGGFTEAFF	TRBV11-1	39		283	4727	N.D.	N.D.	no	
PT3-REC	474	F06REC	CD4_81	Blood	P2	CASSLALGGFTEAFF	TRBV11-1	39		275	2208	N.D.	N.D.	no	
PT3-REC	475	G01REC	CD4_81	Blood	P2	CASSLALGGFTEAFF	TRBV11-1	39		613	4897	N.D.	N.D.	no	
PT3-REC	476	G04REC	CD4_81	Blood	P2	CASSLALGGFTEAFF	TRBV11-1	39		325	7181	N.D.	N.D.	no	
PT3-REC	477	G06REC	CD4_81	Blood	P2	CASSLALGGFTEAFF	TRBV11-1	39		378	1242	N.D.	N.D.	no	
PT3-REC	478	H02REC	CD4_81	Blood	P2	CASSLALGGFTEAFF	TRBV11-1	39		531	3418	N.D.	N.D.	no	
PT3-REC	479	H03REC	CD4_81	Blood	P2	CASSLALGGFTEAFF	TRBV11-1	39		442	5065	N.D.	N.D.	no	
PT3-REC	480	H05REC	CD4_81	Blood	P2	CASSLALGGFTEAFF	TRBV11-1	39		302	2537	N.D.	N.D.	no	
PT3-REC	481	A03REC	CD4_81	Blood	P2	CASSLALGGFTEAFF	TRBV11-1	39		287	7749	N.D.	N.D.	no	
PT3-REC	482	A06REC	CD4_81	Blood	P2	CASSLALGGFTEAFF	TRBV11-1	39		245	1160	N.D.	N.D.	no	
PT3-REC	483	B05REC	CD4_81	Blood	P2	CASSLALGGFTEAFF	TRBV11-1	39		233	1422	N.D.	N.D.	no	
PT3-REC	484	C01REC	CD4_81	Blood	P2	CASSLALGGFTEAFF	TRBV11-1	39		234	1625	N.D.	N.D.	no	
PT3-REC	485	C02REC	CD4_81	Blood	P2	CASSLALGGFTEAFF	TRBV11-1	39		226	1050	N.D.	N.D.	no	
PT3-REC	486	C04REC	CD4_81	Blood	P2	CASSLALGGFTEAFF	TRBV11-1	39		273	4540	N.D.	N.D.	no	
PT3-REC	487	D05REC	CD4_81	Blood	P2	CASSLALGGFTEAFF	TRBV11-1	39		284	9276	1-15	HLA-DR	no	
PT3-REC	488	A05REC	CD4_81	Blood	P2	CASSLALGGFTEAFF	TRBV11-1	39		372	7716	1-15	HLA-DR	no	
PT3-REC	489	B04REC	CD4_82	Blood	P2	CASSLGGASSPLHF	TRBV3	36		241	3099	121-132	HLA-DR	no	
PT3-REC	490	C02REC	CD4_82	Blood	P2	CASSLGGASSPLHF	TRBV3	36		275	13560	121-132	HLA-DR	no	
PT3-REC	491	F01REC	CD4_83	Blood	P2	CASSVWGKGTDTQYF	TRBV2	39		568	1974	1-15	HLA-DR	no	
PT3-REC	492	A02REC	N.D.	Blood	P2	N.D.	N.D.	N.D.		404	3312	N.D.	N.D.	N.D.	
PT3-REC	493	E01REC	N.D.	Blood	P2	N.D.	N.D.	N.D.		457	8360	N.D.	N.D.	N.D.	
PT4-REC	494	F07REC	CD4_84	Blood	P2	CASSLAYEQYF	TRBV8-1	33		71	1091	N.D.	N.D.	no	
PT4-REC	495	B12REC	CD4_84	Blood	P2	CASSLAYEQYF	TRBV8-1	33		134	1231	N.D.	N.D.	no	
PT4-REC	496	H10REC	CD4_85	Blood	P2	CASVNKSRELRADF	TRBV10-1	42		36	1033	N.D.	N.D.	no	
PT4-AC	497	B03AC	N.D.	Blood	P2	N.D.	N.D.	N.D.		78	5078	101-115	HLA-DR	N.D.	
PT4-AC	498	B08AC	N.D.	Blood	P2	N.D.	N.D.	N.D.		101	4097	101-115	N.D.	N.D.	
PT4-AC	499	C05AC	N.D.	Blood	P2	N.D.	N.D.	N.D.		101	4164	101-115	HLA-DR	N.D.	
PT4-AC	500	H02AC	N.D.	Blood	P2	N.D.	N.D.	N.D.		41	5634	101-115	N.D.	N.D.	
PT4-AC	501	A08AC	N.D.	Blood	P2	N.D.	N.D.	N.D.		128	5443	101-115	HLA-DR	N.D.	
PT4-AC	502	B04AC	N.D.	Blood	P2	N.D.	N.D.	N.D.		69	4444	101-115	HLA-DR/DQ	N.D.	
PT4-AC	503	C06AC	N.D.	Blood	P2	N.D.	N.D.	N.D.		116	6044	101-115	N.D.	N.D.	
PT4-AC	504	F02AC	N.D.	Blood	P2	N.D.	N.D.	N.D.		75	5785	101-115	HLA-DR	N.D.	
PT4-AC	505	H05AC	N.D.	Blood	P2	N.D.	N.D.	N.D.		96	9277	101-115	HLA-DR	N.D.	
PT4-AC	506	A06AC	N.D.	Blood	P2	N.D.	N.D.	N.D.		140	9957	101-115	HLA-DR	N.D.	
PT4-AC	507	B06AC	N.D.	Blood	P2	N.D.	N.D.	N.D.		99	5187	101-115	HLA-DR	N.D.	
PT4-AC	508	F05AC	N.D.	Blood	P2	N.D.	N.D.	N.D.		108	2471	101-115	HLA-DR	N.D.	
PT4-AC	509	G04AC	N.D.	Blood	P2	N.D.	N.D.	N.D.		99	6851	101-115	HLA-DR	N.D.	
PT4-AC	510	H06AC	N.D.	Blood	P2	N.D.	N.D.	N.D.		82	3811	101-115	HLA-DR	N.D.	
PT4-AC	511	G03AC	N.D.	Blood	P2	N.D.	N.D.	N.D.		76	1302	N.D.	N.D.	N.D.	
PT4-AC	512	G04AC	N.D.	Blood	P2	N.D.	N.D.	N.D.		26	1388	N.D.	N.D.	N.D.	
PT4-AC	513	E05AC	N.D.	Blood	P2	N.D.	N.D.	N.D.		1157	3804	N.D.	N.D.	N.D.	
PT4-AC	514	A07AC	N.D.	Blood	P2	N.D.	N.D.	N.D.		106	4489	N.D.	N.D.	N.D.	
PT4-AC	515	F01AC	N.D.	Blood	P2	N.D.	N.D.	N.D.		79	2239	N.D.	N.D.	N.D.	
PT4-AC	516	C01AC	N.D.	Blood	P2	N.D.	N.D.	N.D.		73	6564	N.D.	N.D.	N.D.	
PT4-AC	517	F03AC	N.D.	Blood	P2	N.D.	N.D.	N.D.		95	3294	N.D.	N.D.	N.D.	
PT4-REC	518	E12REC	N.D.	Blood	P2	N.D.	N.D.	N.D.		97	1087	N.D.	N.D.	N.D.	

PT4-REC	519	B07REC	N.D.	Blood	P2	N.D.	N.D.	N.D.		64	1406	N.D.	N.D.	N.D.	
PT4-REC	520	C07REC	N.D.	Blood	P2	N.D.	N.D.	N.D.		81	1029	N.D.	N.D.	N.D.	
PT4-REC	521	E07REC	N.D.	Blood	P2	N.D.	N.D.	N.D.		41	1235	N.D.	N.D.	N.D.	
PT4-REC	522	A08REC	N.D.	Blood	P2	N.D.	N.D.	N.D.		74	1546	N.D.	N.D.	N.D.	
PT4-REC	523	C08REC	N.D.	Blood	P2	N.D.	N.D.	N.D.		276	1499	N.D.	N.D.	N.D.	
PT4-REC	524	D08REC	N.D.	Blood	P2	N.D.	N.D.	N.D.		64	1066	N.D.	N.D.	N.D.	
PT4-REC	525	E08REC	N.D.	Blood	P2	N.D.	N.D.	N.D.		64	2294	N.D.	N.D.	N.D.	
PT4-REC	526	A01REC	N.D.	Blood	P2	N.D.	N.D.	N.D.		68	1055	N.D.	N.D.	N.D.	
PT4-REC	527	A02REC	N.D.	Blood	P2	N.D.	N.D.	N.D.		49	1160	N.D.	N.D.	N.D.	
PT4-REC	528	A06REC	N.D.	Blood	P2	N.D.	N.D.	N.D.		131	1293	N.D.	N.D.	N.D.	
PT4-REC	529	B04REC	N.D.	Blood	P2	N.D.	N.D.	N.D.		94	1429	N.D.	N.D.	N.D.	
PT4-REC	530	B05REC	N.D.	Blood	P2	N.D.	N.D.	N.D.		99	1760	N.D.	N.D.	N.D.	
PT4-REC	531	B10REC	N.D.	Blood	P2	N.D.	N.D.	N.D.		67	1208	N.D.	N.D.	N.D.	
PT4-REC	532	C01REC	N.D.	Blood	P2	N.D.	N.D.	N.D.		37	1302	N.D.	N.D.	N.D.	
PT4-REC	533	D05REC	N.D.	Blood	P2	N.D.	N.D.	N.D.		63	1070	N.D.	N.D.	N.D.	
PT4-REC	534	D10REC	N.D.	Blood	P2	N.D.	N.D.	N.D.		39	1555	N.D.	N.D.	N.D.	
PT4-REC	535	F04REC	N.D.	Blood	P2	N.D.	N.D.	N.D.		91	1126	N.D.	N.D.	N.D.	
PT4-REC	536	F12REC	N.D.	Blood	P2	N.D.	N.D.	N.D.		46	1009	N.D.	N.D.	N.D.	
PT4-REC	537	H06REC	N.D.	Blood	P2	N.D.	N.D.	N.D.		56	1691	N.D.	N.D.	N.D.	
PT4-REC	538	A09REC	N.D.	Blood	P2	N.D.	N.D.	N.D.		103	6551	N.D.	N.D.	N.D.	
PT4-REC	539	H07REC	N.D.	Blood	P2	N.D.	N.D.	N.D.		96	2744	N.D.	N.D.	N.D.	
PT4-REC	540	D09REC	N.D.	Blood	P2	N.D.	N.D.	N.D.		57	2699	N.D.	N.D.	N.D.	
PT4-REC	541	F05REC	N.D.	Blood	P2	N.D.	N.D.	N.D.		106	3755	N.D.	N.D.	N.D.	
PT4-REC	542	F07REC	N.D.	Blood	P2	N.D.	N.D.	N.D.		91	1671	N.D.	N.D.	N.D.	
PT4-REC	543	F09REC	N.D.	Blood	P2	N.D.	N.D.	N.D.		84	1598	N.D.	N.D.	N.D.	
PT4-REC	544	G10REC	N.D.	Blood	P2	N.D.	N.D.	N.D.		95	2613	N.D.	N.D.	N.D.	
PT4-REC	545	G11REC	N.D.	Blood	P2	N.D.	N.D.	N.D.		91	7087	N.D.	N.D.	N.D.	
PT5-AC	546	C02AC	CD4_86	Blood	P2	CAISGQKTGELFF	TRBV12-4	33		288	3925	N.D.	N.D.	no	
PT5-AC	547	B03AC	CD4_86	Blood	P2	CAISGQKTGELFF	TRBV12-4	33		247	12072	N.D.	N.D.	no	
PT5-AC	548	A03AC	CD4_87	Blood	P2	CASGGTGAARRDNSPLHF	TRBV6-1	48		247	62980	121-132	HLA-DR	no	
PT5-REC	549	A09REC	CD4_87	Blood	P2	CASGGTGAARRDNSPLHF	TRBV6-1	48		323	26776	121-132	HLA-DR	no	
PT5-REC	550	D10REC	CD4_87	Blood	P2	CASGGTGAARRDNSPLHF	TRBV6-1	48		192	17723	121-132	HLA-DR	no	
PT5-AC	551	C03AC	CD4_88	Blood	P2	CASRNAPNTEAFF	TRBV28	33		189	9571	111-125	HLA-DR	no	
PT5-AC	552	G03AC	CD4_89	Blood	P2	CASRQGSYEQYF	TRBV2	30		214	2471	N.D.	HLA-DR	yes	CLUSTER_6
PT5-AC	553	C07AC	CD4_90	Blood	P2	CASSLGQGQTEAFF	TRBV11-2	36		137	1142	N.D.	HLA-DR	no	
PT5-AC	554	B04AC	CD4_91	Blood	P2	CASSLSPGNTIYF	TRBV7-3	33		270	1513	N.D.	HLA-DR	no	
PT5-AC	555	B08AC	CD4_91	Blood	P2	CASSLSPGNTIYF	TRBV7-3	33		242	1937	N.D.	HLA-DR	no	
PT5-AC	556	E10AC	CD4_91	Blood	P2	CASSLSPGNTIYF	TRBV7-3	33		232	2616	N.D.	HLA-DR	no	
PT5-AC	557	F05AC	CD4_91	Blood	P2	CASSLSPGNTIYF	TRBV7-3	33		257	1329	N.D.	HLA-DR	no	
PT5-AC	558	E06AC	CD4_91	Blood	P2	CASSLSPGNTIYF	TRBV7-3	33		175	3058	N.D.	HLA-DR	no	
PT5-AC	559	C03AC	CD4_91	Blood	P2	CASSLSPGNTIYF	TRBV7-3	33		308	12500	1-15	HLA-DR	no	
PT5-AC	560	E05AC	CD4_91	Blood	P2	CASSLSPGNTIYF	TRBV7-3	33		147	2116	1-15	HLA-DR	no	
PT5-AC	561	A04AC	CD4_91	Blood	P2	CASSLSPGNTIYF	TRBV7-3	33		314	4572	1-15	HLA-DR	no	
PT5-AC	562	D02AC	CD4_91	Blood	P2	CASSLSPGNTIYF	TRBV7-3	33		215	2345	1-15	HLA-DR	no	
PT5-AC	563	D07AC	CD4_91	Blood	P2	CASSLSPGNTIYF	TRBV7-3	33		116	1252	1-15	HLA-DR	no	
PT5-AC	564	H03AC	CD4_91	Blood	P2	CASSLSPGNTIYF	TRBV7-3	33		630	3363	1-15	HLA-DR	no	
PT5-AC	565	H04AC	CD4_91	Blood	P2	CASSLSPGNTIYF	TRBV7-3	33		246	2657	1-15	HLA-DR	no	
PT5-REC	566	E05REC	CD4_91	Blood	P2	CASSLSPGNTIYF	TRBV7-3	33		245	2997	1-15	HLA-DR	no	
PT5-REC	567	H10REC	CD4_91	Blood	P2	CASSLSPGNTIYF	TRBV7-3	33		314	5125	1-15	HLA-DR	no	
PT5-AC	568	H06AC	CD4_92	Blood	P2	CASSPGRFSGNTIYF	TRBV3	39		250	15399	N.D.	N.D.	no	
PT5-AC	569	H01AC	CD4_93	Blood	P2	CASSRTGQYEQYF	TRBV27	33		238	2059	N.D.	HLA-DR	no	
PT5-AC	570	H08AC	CD4_94	Blood	P2	CASSYSRTGSQETQYF	TRBV6-2	42		371	18411	1-15	HLA-DP	no	
PT5-AC	571	G07AC	CD4_94	Blood	P2	CASSYSRTGSQETQYF	TRBV6-2	42		474	3342	1-15	HLA-DP	no	
PT5-AC	572	B07AC	CD4_95	Blood	P2	CAWSVRTEAFF	TRBV30	27		178	17018	121-132	HLA-DP	no	
PT5-AC	573	H07AC	CD4_95	Blood	P2	CAWSVRTEAFF	TRBV30	27		295	47974	121-132	HLA-DP	no	
PT5-AC	574	B06AC	CD4_95	Blood	P2	CAWSVRTEAFF	TRBV30	27		318	37201	121-132	HLA-DP	no	
PT5-AC	575	F09AC	CD4_96	Blood	P2	CASSLTGTVSSYEQYF	TRBV7-3	42		287	36695	101-120	HLA-DR	no	
PT5-AC	576	B06AC	CD4_96	Blood	P2	CASSLTGTVSSYEQYF	TRBV7-3	42		318	37201	101-120	HLA-DR	no	
PT5-AC	577	F01AC	CD4_96	Blood	P2	CASSLTGTVSSYEQYF	TRBV7-3	42		251	33248	101-120	HLA-DR	no	
PT5-AC	578	F04AC	CD4_96	Blood	P2	CASSLTGTVSSYEQYF	TRBV7-3	42		253	22298	101-120	HLA-DR	no	
PT5-REC	579	C02REC	CD4_96	Blood	P2	CASSLTGTVSSYEQYF	TRBV7-3	42		361	50511	101-120	HLA-DR	no	
PT5-REC	580	A01REC	CD4_96	Blood	P2	CASSLTGTVSSYEQYF	TRBV7-3	42		312	21805	101-120	HLA-DR	no	
PT5-REC	581	B07REC	CD4_96	Blood	P2	CASSLTGTVSSYEQYF	TRBV7-3	42		238	27105	101-120	HLA-DR	no	
PT5-REC	582	E11REC	CD4_96	Blood	P2	CASSLTGTVSSYEQYF	TRBV7-3	42		192	15690	101-120	HLA-DR	no	
PT5-REC	583	F11REC	CD4_96	Blood	P2	CASSLTGTVSSYEQYF	TRBV7-3	42		204	23061	101-120	HLA-DR	no	
PT5-REC	584	G03REC	CD4_96	Blood	P2	CASSLTGTVSSYEQYF	TRBV7-3	42		234	24358	101-120	HLA-DR	no	
PT5-REC	585	D04REC	CD4_96	Blood	P2	CASSLTGTVSSYEQYF	TRBV7-3	42		253	37723	N.D.	HLA-DR	no	
PT5-REC	586	B10REC	CD4_97	Blood	P2	CASIPTGGLGQPQHF	TRBV6-6	39		278	21207	101-120	HLA-DR	no	
PT5-REC	587	C06REC	CD4_98	Blood	P2	CASSEGTRAYNEQFF	TRBV9	39		213	12551	N.D.	HLA-DR	no	
PT5-REC	588	G09REC	CD4_99	Blood	P2	CASSEVGGRKGTGELFF	TRBV2	45		279	26151	101-120	HLA-DR	no	
PT5-REC	589	G05REC	CD4_100	Blood	P2	CASSPPGPSPLHF	TRBV12-4	33		242	9453	N.D.	N.D.	no	
PT5-REC	590	G11REC	CD4_100	Blood	P2	CASSPPGPSPLHF	TRBV12-4	33		203	11348	N.D.	N.D.	no	
PT5-REC	591	H11REC	CD4_100	Blood	P2	CASSPPGPSPLHF	TRBV12-4	33		199	3574	N.D.	HLA-DR/DQ	no	
PT5-REC	592	B04REC	CD4_101	Blood	P2	CASSPRRQEAFF	TRBV18	35		261	1633	N.D.	N.D.	no	
PT5-REC	593	G02REC	CD4_102	Blood	P2	CASSSGTGSVAFF	TRBV12-3	33		198	15981	101-115	HLA-DR	no	
PT5-REC	594	B03REC	CD4_102	Blood	P2	CASSSGTGSVAFF	TRBV12-3	33		238	30701	101-115	HLA-DR	no	
PT5-REC	595	F05REC	CD4_103	Blood	P2	CASSTGVLTGDMYF	TRBV19	36		220	1895	1-15	HLA-DR	no	
PT5-REC	596	E07REC	CD4_104	Blood	P2	CASSTGVLTGELFF	TRBV19	36		144	1799	1-15	HLA-DR	no	
PT5-REC	597	D02REC	CD4_104	Blood	P2	CASSTGVLTGELFF	TRBV19	36		211	2232	1-15	HLA-DR	no	
PT5-AC	598	A04AC	N.D.	Blood	P2	N.D.	N.D.	N.D.		225	35375	101-120	HLA-DR	N.D.	
PT5-AC	599	D12AC	N.D.	Blood	P2	N.D.	N.D.	N.D.		130	23020	101-120	HLA-DR	N.D.	
PT5-AC	600	E11AC	N.D.	Blood	P2	N.D.	N.D.	N.D.		200	8382	101-120	HLA-DR	N.D.	
PT5-AC	601	G09AC	N.D.	Blood	P2	N.D.	N.D.	N.D.		215	11508	101-120	HLA-DR	N.D.	
PT5-AC	602	B03AC	N.D.	Blood	P2	N.D.	N.D.	N.D.		136	3634	101-120	HLA-DR	N.D.	
PT5-AC	603	F07AC	N.D.	Blood	P2	N.D.	N.D.	N.D.		425	5962	1-15	HLA-DP	N.D.	
PT5-AC	604	H07AC	N.D.	Blood	P2	N.D.	N.D.	N.D.		255	19619	1-15	N.D.	N.D.	
PT5-AC	605	A02AC	N.D.	Blood	P2	N.D.	N.D.	N.D.		279	1812	N.D.	N.D.	N.D.	
PT5-AC	606	D04AC	N.D.	Blood	P2	N.D.	N.D.	N.D.		270	1513	N.D.	HLA-DR	N.D.	
PT5-REC	607	H04REC	N.D.	Blood	P2	N.D.	N.D.	N.D.		336	22725	101-115	HLA-DR	N.D.	
PT5-REC	608	C10REC	N.D.	Blood	P2	N.D.	N.D.	N.D.		216	22608	101-120	HLA-DR	N.D.	
PT5-REC	609	D03REC	N.D.	Blood	P2	N.D.	N.D.	N.D.		239	41680	101-120	HLA-DR	N.D.	
PT5-REC	610	E09REC	N.D.	Blood	P2	N.D.	N.D.	N.D.		140	19199	101-120	HLA-DR	N.D.	
PT5-REC	611	C01REC	N.D.	Blood	P2	N.D.	N.D.	N.D.		238	12385	1-15	N.D.	N.D.	
PT5-REC	612	C03REC	N.D.	Blood	P2	N.D.	N.D.	N.D.		247	20298	1-15	HLA-DR	N.D.	
PT5-REC	613	G07REC	N.D.	Blood	P2	N.D.	N.D.	N.D.		175	1112	N.D.	HLA-DR	N.D.	
PT6-AC	614	D06AC	CD4_105	Blood	P2	CASSIEWANTEAFF	TRBV19	36		200	6488	N.D.	N.D.	no	
PT6-AC	615	D03AC	CD4_106	Blood	P2	CASSKLAANTGELFF	TRBV9	39		365	9477	N.D.	N.D.	no	
PT6-AC	616	D02AC	CD4_107	Blood	P2	CASSLEVGEQFF	TRBV7-2	30		247	10004	N.D.	N.D.	yes	CLUSTER_2
PT6-AC	617	D04AC	CD4_107	Blood	P2	CASSLEVGEQFF	TRBV7-2	30		118	7665	N.D.	N.D.	yes	CLUSTER_2
PT6-AC	618	D05AC	CD4_107	Blood	P2	CASSLEVGEQFF	TRBV7-2	30		216	13648	N.D.	N.D.	yes	CLUSTER_2
PT6-AC	619	D09AC	CD4_107	Blood	P2	CASSLEVGEQFF	TRBV7-2	30		112	4617	N.D.	N.D.	yes	CLUSTER_2
PT6-AC	620	E02AC	CD4_107	Blood	P2	CASSLEVGEQFF	TRBV7-2	30		150	6123	N.D.	N.D.	yes	CLUSTER_2
PT6-AC	621	F01AC	CD4_107	Blood	P2	CASSLEVGEQFF	TRBV7-2	30		166	3460	N.D.	N.D.	yes	CLUSTER_2
PT6-AC	622	F02AC	CD4_107	Blood	P2	CASSLEVGEQFF	TRBV7-2	30		239	6917	N.D.	N.D.	yes	CLUSTER_2
PT6-AC	623	F04AC	CD4_107	Blood	P2	CASSLEVGEQFF	TRBV7-2	30		122	8054	N.D.	N.D.	yes	CLUSTER_2
PT6-AC	624	F05AC	CD4_107	Blood	P2	CASSLEVGEQFF	TRBV7-2	30		224	3050	N.D.	N.D.	yes	CLUSTER_2
PT6-AC	625	F07AC	CD4_107	Blood	P2	CASSLEVGEQFF	TRBV7-2	30		108	13801	N.D.	N.D.	yes	CLUSTER_2
PT6-AC	626	F10AC	CD4_107	Blood	P2	CASSLEVGEQFF	TRBV7-2	30		322	6233	N.D.	N.D.	yes	CLUSTER_2
PT6-AC	627	F12AC	CD4_107	Blood	P2	CASSLEVGEQFF	TRBV7-2	30		130	11707	N.D.	N.D.	yes	CLUSTER_2
PT6-AC	628	G02AC	CD4_107	Blood	P2	CASSLEVGEQFF	TRBV7-2	30		242	5938	N.D.	N.D.	yes	CLUSTER_2
PT6-AC	629	G04AC	CD4_107	Blood	P2	CASSLEVGEQFF	TRBV7-2	30		152	8369	N.D.	N.D.	yes	CLUSTER_2
PT6-AC	630	G05AC	CD4_107	Blood	P2	CASSLEVGEQFF	TRBV7-2	30		121	2619	N.D.	N.D.	yes	CLUSTER_2
PT6-AC	631	G06AC	CD4_107	Blood	P2	CASSLEVGEQFF	TRBV7-2	30		148	5169	N.D.	N.D.	yes	CLUSTER_2
PT6-AC	632	G07AC	CD4_107	Blood	P2	CASSLEVGEQFF	TRBV7-2	30		124	2832	N.D.	N.D.	yes	CLUSTER_2
PT6-AC	633	G10AC	CD4_107	Blood	P2	CASSLEVGEQFF	TRBV7-2	30		147	7673	N.D.	N.D.	yes	CLUSTER_2
PT6-AC	634	G11AC	CD4_107	Blood	P2	CASSLEVGEQFF	TRBV7-2	30		147	9854	N.D.	N.D.	yes	CLUSTER_2
PT6-AC	635	H04AC	CD4_107	Blood	P2	CASSLEVGEQFF	TRBV7-2	30		195	4132	N.D.	N.D.	yes	CLUSTER_2
PT6-AC	636	H08AC	CD4_107	Blood	P2	CASSLEVGEQFF	TRBV7-2	30		183	3892	N.D.	N.D.	yes	CLUSTER_2
PT6-AC	637	H10AC	CD4_107	Blood	P2	CASSLEVGEQFF	TRBV7-2	30		191	15945	N.D.	N.D.	yes	CLUSTER_2
PT6-AC	638	H11AC	CD4_107	Blood	P2	CASSLEVGEQFF	TRBV7-2	30		179	9193	N.D.	N.D.	yes	CLUSTER_2
PT6-AC	639	A10AC	CD4_107	Blood	P2	CASSLEVGEQFF	TRBV7-2	30		118	7509	N.D.	N.D.	yes	CLUSTER_2
PT6-AC	640	A11AC	CD4_107	Blood	P2	CASSLEVGEQFF	TRBV7-2	30		133	26855	N.D.	N.D.	yes	CLUSTER_2
PT6-AC	641	B10AC	CD4_107	Blood	P2	CASSLEVGEQFF	TRBV7-2	30		184	10172	N.D.	N.D.	yes	CLUSTER_2
PT6-AC	642	C10AC	CD4_107	Blood	P2	CASSLEVGEQFF	TRBV7-2	30		149	10313	N.D.	N.D.	yes	CLUSTER_2
PT6-AC	643	C11AC	CD4_107	Blood	P2	CASSLEVGEQFF	TRBV7-2	30		63	3597	N.D.	N.D.	yes	CLUSTER_2
PT6-AC	644	D01AC	CD4_107	Blood	P2	CASSLEVGEQFF	TRBV7-2	30		146	4247	N.D.	N.D.	yes	CLUSTER_2
PT6-AC	645	D04AC	CD4_107	Blood	P2	CASSLEVGEQFF	TRBV7-2	30		192	8419	N.D.	N.D.	yes	CLUSTER_2
PT6-AC	646	D08AC	CD4_107	Blood	P2	CASSLEVGEQFF	TRBV7-2	30		136	20804	N.D.	N.D.	yes	CLUSTER_2
PT6-AC	647	D09AC	CD4_107	Blood	P2	CASSLEVGEQFF	TRBV7-2	30		176	3559	N.D.	N.D.	yes	CLUSTER_2
PT6-AC	648	E05AC	CD4_107	Blood	P2	CASSLEVGEQFF	TRBV7-2	30		147	6373	N.D.	N.D.	yes	CLUSTER_2

PT6-AC	649	E06AC	CD4_107	Blood	P2	CASSLEVGEQFF	TRBV7-2	30		167	13372	N.D.	N.D.	yes	CLUSTER_2
PT6-AC	650	E09AC	CD4_107	Blood	P2	CASSLEVGEQFF	TRBV7-2	30		168	15292	N.D.	N.D.	yes	CLUSTER_2
PT6-AC	651	E10AC	CD4_107	Blood	P2	CASSLEVGEQFF	TRBV7-2	30		118	12617	N.D.	N.D.	yes	CLUSTER_2
PT6-AC	652	E12AC	CD4_107	Blood	P2	CASSLEVGEQFF	TRBV7-2	30		179	9977	N.D.	N.D.	yes	CLUSTER_2
PT6-AC	653	F04AC	CD4_107	Blood	P2	CASSLEVGEQFF	TRBV7-2	30		155	6690	N.D.	N.D.	yes	CLUSTER_2
PT6-AC	654	F06AC	CD4_107	Blood	P2	CASSLEVGEQFF	TRBV7-2	30		200	8686	N.D.	N.D.	yes	CLUSTER_2
PT6-AC	655	F08AC	CD4_107	Blood	P2	CASSLEVGEQFF	TRBV7-2	30		179	3204	N.D.	N.D.	yes	CLUSTER_2
PT6-AC	656	F09AC	CD4_107	Blood	P2	CASSLEVGEQFF	TRBV7-2	30		179	18573	N.D.	N.D.	yes	CLUSTER_2
PT6-AC	657	F11AC	CD4_107	Blood	P2	CASSLEVGEQFF	TRBV7-2	30		130	11446	N.D.	N.D.	yes	CLUSTER_2
PT6-AC	658	F12AC	CD4_107	Blood	P2	CASSLEVGEQFF	TRBV7-2	30		208	22231	N.D.	N.D.	yes	CLUSTER_2
PT6-AC	659	G01AC	CD4_107	Blood	P2	CASSLEVGEQFF	TRBV7-2	30		166	6066	N.D.	N.D.	yes	CLUSTER_2
PT6-AC	660	G03AC	CD4_107	Blood	P2	CASSLEVGEQFF	TRBV7-2	30		157	17848	N.D.	N.D.	yes	CLUSTER_2
PT6-AC	661	G05AC	CD4_107	Blood	P2	CASSLEVGEQFF	TRBV7-2	30		195	12139	N.D.	N.D.	yes	CLUSTER_2
PT6-AC	662	G07AC	CD4_107	Blood	P2	CASSLEVGEQFF	TRBV7-2	30		140	6074	N.D.	N.D.	yes	CLUSTER_2
PT6-AC	663	G09AC	CD4_107	Blood	P2	CASSLEVGEQFF	TRBV7-2	30		155	8863	N.D.	N.D.	yes	CLUSTER_2
PT6-AC	664	G11AC	CD4_107	Blood	P2	CASSLEVGEQFF	TRBV7-2	30		191	3753	N.D.	N.D.	yes	CLUSTER_2
PT6-AC	665	H01AC	CD4_107	Blood	P2	CASSLEVGEQFF	TRBV7-2	30		141	21333	N.D.	N.D.	yes	CLUSTER_2
PT6-AC	666	H02AC	CD4_107	Blood	P2	CASSLEVGEQFF	TRBV7-2	30		202	14426	N.D.	N.D.	yes	CLUSTER_2
PT6-AC	667	H05AC	CD4_107	Blood	P2	CASSLEVGEQFF	TRBV7-2	30		141	7363	N.D.	N.D.	yes	CLUSTER_2
PT6-AC	668	H06AC	CD4_107	Blood	P2	CASSLEVGEQFF	TRBV7-2	30		160	16876	N.D.	N.D.	yes	CLUSTER_2
PT6-AC	669	H11AC	CD4_107	Blood	P2	CASSLEVGEQFF	TRBV7-2	30		151	19236	N.D.	N.D.	yes	CLUSTER_2
PT6-AC	670	F10AC	CD4_107	Blood	P2	CASSLEVGEQFF	TRBV7-2	30		114	15405	N.D.	N.D.	yes	CLUSTER_2
PT6-AC	671	C12AC	CD4_107	Blood	P2	CASSLEVGEQFF	TRBV7-2	30		145	12877	26-40	HLA-DR	yes	CLUSTER_2
PT6-AC	672	E09AC	CD4_107	Blood	P2	CASSLEVGEQFF	TRBV7-2	30		104	9344	26-40	HLA-DR	yes	CLUSTER_2
PT6-AC	673	A10AC	CD4_107	Blood	P2	CASSLEVGEQFF	TRBV7-2	30		243	3724	26-40	HLA-DR	yes	CLUSTER_2
PT6-AC	674	B11AC	N.D.	Blood	P2	N.D.	N.D.	N.D.		224	14773	N.D.	N.D.	N.D.	
PT6-AC	675	E10AC	N.D.	Blood	P2	N.D.	N.D.	N.D.		289	12333	N.D.	N.D.	N.D.	
PT6-AC	676	E11AC	N.D.	Blood	P2	N.D.	N.D.	N.D.		244	11072	N.D.	N.D.	N.D.	
PT6-AC	677	H02AC	N.D.	Blood	P2	N.D.	N.D.	N.D.		266	19013	N.D.	N.D.	N.D.	
PT6-AC	678	H05AC	N.D.	Blood	P2	N.D.	N.D.	N.D.		148	3534	N.D.	N.D.	N.D.	
PT6-AC	679	E04AC	N.D.	Blood	P2	N.D.	N.D.	N.D.		179	6914	N.D.	N.D.	N.D.	
PT6-AC	680	E07AC	N.D.	Blood	P2	N.D.	N.D.	N.D.		152	7228	N.D.	N.D.	N.D.	
PT6-AC	681	F07AC	N.D.	Blood	P2	N.D.	N.D.	N.D.		136	3044	N.D.	N.D.	N.D.	
PT6-AC	682	H04AC	N.D.	Blood	P2	N.D.	N.D.	N.D.		414	7600	N.D.	N.D.	N.D.	
PT6-AC	683	F11AC	N.D.	Blood	P2	N.D.	N.D.	N.D.		224	4900	N.D.	N.D.	N.D.	
PT7-REC	684	CL46REC	CD4_108	Blood	P2	CAISSDRAGEEKLFF	TRBV10-3	39	in PT11	2482	8470	N.D.	HLA-DR	no	
PT7-REC	685	CL65REC	CD4_108	Blood	P2	CAISSDRAGEEKLFF	TRBV10-3	39	in PT11	1111	4977	N.D.	HLA-DR	no	
PT7-REC	686	CL03REC	CD4_108	Blood	P2	CAISSDRAGEEKLFF	TRBV10-3	39	in PT11	382	3419	N.D.	HLA-DR	no	
PT7-REC	687	CL34REC	CD4_109	Blood	P2	CAITTGSANYGYTF	TRBV19	40		1344	14077	N.D.	HLA-DR	no	
PT7-REC	688	CL62REC	CD4_109	Blood	P2	CAITTGSANYGYTF	TRBV19	40		2007	19001	N.D.	HLA-DR	no	
PT7-REC	689	CL33REC	CD4_109	Blood	P2	CAITTGSANYGYTF	TRBV19	40		1970	17893	51-65	HLA-DR	no	
PT7-REC	690	CL36REC	CD4_109	Blood	P2	CAITTGSANYGYTF	TRBV19	40		1290	14356	51-65	HLA-DR	no	
PT7-REC	691	CL27REC	CD4_110	Blood	P2	CASRRTVQETQYF	TRBV27	33	in PT11	587	2370	1-15	HLA-DR	yes	
PT7-REC	692	CL05REC	CD4_111	Blood	P2	CASRTGEYSNQPQHF	TRBV6-6	39	in PT10	491	4489	N.D.	HLA-DR	no	
PT7-REC	693	CL64REC	CD4_111	Blood	P2	CASRTGEYSNQPQHF	TRBV6-6	39	in PT10	3031	19403	51-65	HLA-DR	yes	
PT7-REC	694	CL41REC	CD4_112	Blood	P2	CASSEPGQGRLNTEAFF	TRBV6-6	45		3492	15903	N.D.	HLA-DR	no	
PT7-REC	695	CL23REC	CD4_113	Blood	P2	CASSIGGASTDTQYF	TRBV19	39		152	2197	N.D.	N.D.	no	
PT7-REC	696	CL61REC	CD4_113	Blood	P2	CASSIGGASTDTQYF	TRBV19	39		144	4956	81-95	HLA-DR	no	
PT7-REC	697	CL28REC	CD4_113	Blood	P2	CASSIGGASTDTQYF	TRBV19	39		135	2123	81-95	N.D.	no	
PT7-REC	698	CL11REC	CD4_113	Blood	P2	CASSIGGASTDTQYF	TRBV19	39		3652	8663	81-95	HLA-DR	no	
PT7-REC	699	CL13REC	CD4_113	Blood	P2	CASSIGGASTDTQYF	TRBV19	39		39	9281	81-95	HLA-DR	no	
PT7-REC	700	CL50REC	CD4_113	Blood	P2	CASSIGGASTDTQYF	TRBV19	39		988	8280	81-95	HLA-DR	no	
PT7-REC	701	CL61REC	CD4_113	Blood	P2	CASSIGGASTDTQYF	TRBV19	39		208	9365	81-95	HLA-DR	no	
PT7-REC	702	CL60REC	CD4_113	Blood	P2	CASSIGGASTDTQYF	TRBV19	39		138	6544	81-95	HLA-DR	no	
PT7-REC	703	CL11REC	CD4_114	Blood	P2	CASSQEDGRGEQYF	TRBV28	36	in PT11	1116	5875	96-110	HLA-DR	no	
PT7-REC	704	CL51REC	CD4_115	Blood	P2	CASSQGQGTDTQYF	TRBV4-1	36		414	8683	1-15	N.D.	yes	CLUSTER_4
PT7-REC	705	CL48REC	CD4_116	Blood	P2	CASSSGLAGAYEQYF	TRBV6-2	39		1717	6365	N.D.	HLA-DR	no	CLUSTER_3
PT7-REC	706	CL71REC	CD4_116	Blood	P2	CASSSGLAGAYEQYF	TRBV6-2	39		1111	4977	N.D.	HLA-DR	no	CLUSTER_3
PT7-REC	707	CL29REC	CD4_116	Blood	P2	CASSSGLAGAYEQYF	TRBV6-2	39		333	2018	N.D.	HLA-DR	no	CLUSTER_3
PT7-REC	708	CL39REC	CD4_117	Blood	P2	CASSSGLAGYYEQYF	TRBV6-2	39		261	5431	N.D.	HLA-DR	no	
PT7-REC	709	CL12REC	CD4_118	Blood	P2	CASSSGTSGREQYF	TRBV19	30	in PT11	439	3160	N.D.	HLA-DR	no	
PT7-REC	710	CL10REC	CD4_118	Blood	P2	CASSSGTSGREQYF	TRBV19	30	in PT11	674	2883	N.D.	HLA-DR	no	
PT7-REC	711	CL14REC	CD4_118	Blood	P2	CASSSGTSGREQYF	TRBV19	30	in PT11	182	5086	N.D.	N.D.	no	
PT7-REC	712	CL30REC	CD4_118	Blood	P2	CASSSGTSGREQYF	TRBV19	30	in PT11	681	3816	N.D.	HLA-DR	no	
PT7-REC	713	CL33REC	CD4_118	Blood	P2	CASSSGTSGREQYF	TRBV19	30	in PT11	859	4580	106-120	HLA-DR	yes	
PT7-REC	714	CL03REC	CD4_119	Blood	P2	CASSSSTLMNTEAFF	TRBV7-2	39		367	3876	N.D.	HLA-DR	no	
PT7-REC	715	CL64REC	CD4_120	Blood	P2	CVSSTRWGGTQGEQHF	TRBV18	42		2372	7433	106-120	HLA-DR	no	
PT7-REC	716	CL10REC	N.D.	Blood	P2	N.D.	N.D.	N.D.		28	14525	N.D.	N.D.	N.D.	
PT7-REC	717	CL15REC	N.D.	Blood	P2	N.D.	N.D.	N.D.		1025	12850	N.D.	N.D.	N.D.	
PT7-REC	718	CL15REC	N.D.	Blood	P2	N.D.	N.D.	N.D.		2360	19898	N.D.	N.D.	N.D.	
PT7-REC	719	CL17REC	N.D.	Blood	P2	N.D.	N.D.	N.D.		5605	22457	N.D.	N.D.	N.D.	
PT7-REC	720	CL20REC	N.D.	Blood	P2	N.D.	N.D.	N.D.		956	12562	N.D.	N.D.	N.D.	
PT7-REC	721	CL22REC	N.D.	Blood	P2	N.D.	N.D.	N.D.		1463	5140	N.D.	N.D.	N.D.	
PT7-REC	722	CL23REC	N.D.	Blood	P2	N.D.	N.D.	N.D.		2993	18591	N.D.	N.D.	N.D.	
PT7-REC	723	CL25REC	N.D.	Blood	P2	N.D.	N.D.	N.D.		6666	19164	N.D.	N.D.	N.D.	
PT7-REC	724	CL27REC	N.D.	Blood	P2	N.D.	N.D.	N.D.		199	3785	N.D.	N.D.	N.D.	
PT7-REC	725	CL32REC	N.D.	Blood	P2	N.D.	N.D.	N.D.		980	3715	N.D.	N.D.	N.D.	
PT7-REC	726	CL34REC	N.D.	Blood	P2	N.D.	N.D.	N.D.		3825	16194	N.D.	N.D.	N.D.	
PT7-REC	727	CL43REC	N.D.	Blood	P2	N.D.	N.D.	N.D.		126	2780	N.D.	N.D.	N.D.	
PT7-REC	728	CL44REC	N.D.	Blood	P2	N.D.	N.D.	N.D.		2042	19351	N.D.	N.D.	N.D.	
PT7-REC	729	CL47REC	N.D.	Blood	P2	N.D.	N.D.	N.D.		1385	5956	N.D.	N.D.	N.D.	
PT7-REC	730	CL51REC	N.D.	Blood	P2	N.D.	N.D.	N.D.		29	4046	N.D.	N.D.	N.D.	
PT7-REC	731	CL52REC	N.D.	Blood	P2	N.D.	N.D.	N.D.		1763	19771	N.D.	N.D.	N.D.	
PT7-REC	732	CL53REC	N.D.	Blood	P2	N.D.	N.D.	N.D.		30	6310	N.D.	N.D.	N.D.	
PT7-REC	733	CL55REC	N.D.	Blood	P2	N.D.	N.D.	N.D.		1070	13973	N.D.	N.D.	N.D.	
PT7-REC	734	CL60REC	N.D.	Blood	P2	N.D.	N.D.	N.D.		2572	16365	N.D.	N.D.	N.D.	
PT7-REC	735	CL63REC	N.D.	Blood	P2	N.D.	N.D.	N.D.		3639	10547	N.D.	N.D.	N.D.	
PT7-REC	736	CL73REC	N.D.	Blood	P2	N.D.	N.D.	N.D.		1659	5709	N.D.	N.D.	N.D.	
PT7-REC	737	CL74REC	N.D.	Blood	P2	N.D.	N.D.	N.D.		126	1852	N.D.	N.D.	N.D.	
PT9-AC	738	H01AC	CD4_121	Blood	P2	CASMHPGTTTEAFF	TRBV19	36		1279	5865	N.D.	N.D.	no	
PT9-AC	739	B03AC	CD4_122	Blood	P2	CASSEFQGRYF	TRBV6-1	33		222	9778	N.D.	N.D.	no	
PT9-AC	740	A08AC	CD4_123	Blood	P2	CASSLSDREIPDNEQFF	TRBV5-5	45	in PT11	1486	29306	N.D.	N.D.	no	
PT9-AC	741	B01AC	CD4_123	Blood	P2	CASSLSDREIPDNEQFF	TRBV5-5	45	in PT11	25	21267	N.D.	N.D.	no	
PT9-AC	742	B07AC	CD4_123	Blood	P2	CASSLSDREIPDNEQFF	TRBV5-5	45	in PT11	326	58546	N.D.	N.D.	no	
PT9-AC	743	A01AC	CD4_124	Blood	P2	CASSLVSSGIPTDTQYF	TRBV7-2	45		1461	16766	N.D.	N.D.	no	
PT9-AC	744	B05AC	CD4_125	Blood	P2	CASSRAGDGIYEQYF	TRBV18	39		131	25049	N.D.	N.D.	no	
PT9-AC	745	B04AC	CD4_125	Blood	P2	CASSRAGDGIYEQYF	TRBV18	39		108	7214	N.D.	HLA-DR	no	
PT9-AC	746	H07AC	CD4_126	Blood	P2	CAWSVRGRGNGYTF	TRBV30	36		4239	11824	N.D.	N.D.	no	
PT9-AC	747	C09AC	CD4_126	Blood	P2	CAWSVRGRGNGYTF	TRBV30	36		1078	3727	N.D.	N.D.	no	
PT9-REC	748	C11REC	CD4_127	Blood	P2	CASSHPGGPRNTEAFF	TRBV19	42		534	2994	N.D.	N.D.	no	
PT9-REC	749	E08REC	CD4_128	Blood	P2	CASSPPGTSNYGYTF	TRBV28	39		609	7001	N.D.	N.D.	no	
PT9-REC	750	F06REC	CD4_129	Blood	P2	CASSSWGGNTEAFF	TRBV7-8	36		337	3654	N.D.	N.D.	no	
PT9-REC	751	H01REC	CD4_129	Blood	P2	CASSSWGGNTEAFF	TRBV7-8	36		2584	7456	N.D.	N.D.	no	
PT9-REC	752	A01REC	CD4_129	Blood	P2	CASSSWGGNTEAFF	TRBV7-8	36		56	5174	N.D.	N.D.	no	
PT9-REC	753	A08REC	CD4_129	Blood	P2	CASSSWGGNTEAFF	TRBV7-8	36		1214	3199	N.D.	N.D.	no	
PT9-AC	754	A11AC	N.D.	Blood	P2	N.D.	N.D.	N.D.		490	18443	N.D.	N.D.	N.D.	
PT9-AC	755	A02AC	N.D.	Blood	P2	N.D.	N.D.	N.D.		449	5187	N.D.	N.D.	N.D.	
PT9-AC	756	A03AC	N.D.	Blood	P2	N.D.	N.D.	N.D.		86	3156	N.D.	N.D.	N.D.	
PT9-AC	757	A07AC	N.D.	Blood	P2	N.D.	N.D.	N.D.		96	2056	N.D.	N.D.	N.D.	
PT9-AC	758	B02AC	N.D.	Blood	P2	N.D.	N.D.	N.D.		43	9184	N.D.	N.D.	N.D.	
PT9-AC	759	B08AC	N.D.	Blood	P2	N.D.	N.D.	N.D.		251	2248	N.D.	N.D.	N.D.	
PT9-REC	760	C01REC	N.D.	Blood	P2	N.D.	N.D.	N.D.		601	2392	N.D.	N.D.	N.D.	
PT9-REC	761	G05REC	N.D.	Blood	P2	N.D.	N.D.	N.D.		822	2530	N.D.	N.D.	N.D.	
PT9-REC	762	H07REC	N.D.	Blood	P2	N.D.	N.D.	N.D.		4674	14944	N.D.	N.D.	N.D.	
PT9-REC	763	H08REC	N.D.	Blood	P2	N.D.	N.D.	N.D.		1632	7819	N.D.	N.D.	N.D.	
PT10-AC	764	C05AC	CD4_130	Blood	P2	CASEPTRRGLKQPQHF	TRBV19	43		106	13322	N.D.	HLA-DR	no	CLUSTER_5
PT10-AC	765	C09AC	CD4_131	Blood	P2	CASSLVLSAQGAYNEQFF	TRBV7-8	48		98	2191	N.D.	HLA-DR	no	
PT10-AC	766	G04AC	CD4_132	Blood	P2	CASSTVGTLNYGYTF	TRBV19	39		130	2329	N.D.	N.D.	no	
PT10-AC	767	H08AC	CD4_132	Blood	P2	CASSTVGTLNYGYTF	TRBV19	39		94	2775	N.D.	N.D.	no	
PT10-AC	768	G05AC	CD4_132	Blood	P2	CASSTVGTLNYGYTF	TRBV19	39		122	1815	26-40	N.D.	no	
PT10-AC	769	A05AC	CD4_43	Blood	P2	CASTEQWTYSGNTIYF	TRBV19	42		298	3874	N.D.	N.D.	no	
PT10-AC	770	A06AC	CD4_43	Blood	P2	CASTEQWTYSGNTIYF	TRBV19	42		123	4357	N.D.	N.D.	no	
PT10-AC	771	B04AC	CD4_43	Blood	P2	CASTEQWTYSGNTIYF	TRBV19	42		108	2700	N.D.	HLA-DR	no	
PT10-AC	772	B06AC	CD4_43	Blood	P2	CASTEQWTYSGNTIYF	TRBV19	42		101	4258	N.D.	HLA-DR	no	
PT10-AC	773	B08AC	CD4_43	Blood	P2	CASTEQWTYSGNTIYF	TRBV19	42		138	3423	N.D.	HLA-DR	no	
PT10-AC	774	E01AC	CD4_43	Blood	P2	CASTEQWTYSGNTIYF	TRBV19	42		147	4298	N.D.	N.D.	no	
PT10-AC	775	A08AC	CD4_43	Blood	P2	CASTEQWTYSGNTIYF	TRBV19	42		113	2738	N.D.	HLA-DR	no	
PT10-AC	776	E03AC	CD4_133	Blood	P2	CSAREAGTHHEQFF	TRBV20	36		436	4316	36-55	HLA-DR	no	
PT10-AC	777	D10AC	CD4_134	Blood	P2	CSARQVGGAGKPQHF	TRBV20	39		157	2032	N.D.	N.D.	no	
PT10-AC	778	A07AC	N.D.	Blood	P2	N.D.	N.D.	N.D.		157	1070	N.D.	N.D.	N.D.	

PT10-AC	779	B01AC	N.D.	Blood	P2	N.D.	N.D.	N.D.		186	1773	N.D.	N.D.	N.D.	
PT10-AC	780	C04AC	N.D.	Blood	P2	N.D.	N.D.	N.D.		133	1636	N.D.	N.D.	N.D.	
PT10-AC	781	D08AC	N.D.	Blood	P2	N.D.	N.D.	N.D.		171	6733	N.D.	N.D.	N.D.	
PT10-AC	782	D11AC	N.D.	Blood	P2	N.D.	N.D.	N.D.		212	2338	N.D.	N.D.	N.D.	
PT10-AC	783	E06AC	N.D.	Blood	P2	N.D.	N.D.	N.D.		105	2316	N.D.	N.D.	N.D.	
PT10-AC	784	E09AC	N.D.	Blood	P2	N.D.	N.D.	N.D.		212	14790	N.D.	N.D.	N.D.	
PT10-AC	785	E11AC	N.D.	Blood	P2	N.D.	N.D.	N.D.		152	7764	N.D.	N.D.	N.D.	
PT10-AC	786	E12AC	N.D.	Blood	P2	N.D.	N.D.	N.D.		118	17795	N.D.	N.D.	N.D.	
PT10-AC	787	F09AC	N.D.	Blood	P2	N.D.	N.D.	N.D.		144	3975	N.D.	N.D.	N.D.	
PT10-AC	788	F11AC	N.D.	Blood	P2	N.D.	N.D.	N.D.		142	16557	N.D.	N.D.	N.D.	
PT10-AC	789	G02AC	N.D.	Blood	P2	N.D.	N.D.	N.D.		128	2403	N.D.	N.D.	N.D.	
PT10-AC	790	H02AC	N.D.	Blood	P2	N.D.	N.D.	N.D.		76	2436	N.D.	N.D.	N.D.	
PT12-AC	791	G04AC	CD4_135	Blood	P2	CASSQASGVGNTIYF	TRBV3	45		126	1471	51-65	HLA-DR	no	
PT12-AC	792	G04AC	N.D.	Blood	P2	N.D.	N.D.	N.D.		252	2128	N.D.	N.D.	N.D.	
PT12-AC	793	C01AC	N.D.	Blood	P2	N.D.	N.D.	N.D.		181	1777	N.D.	N.D.	N.D.	
PT12-AC	794	A03AC	N.D.	Blood	P2	N.D.	N.D.	N.D.		148	1379	N.D.	N.D.	N.D.	
PT12-AC	795	F03AC	N.D.	Blood	P2	N.D.	N.D.	N.D.		136	5515	N.D.	N.D.	N.D.	
PT12-AC	796	C05AC	N.D.	Blood	P2	N.D.	N.D.	N.D.		122	1261	N.D.	N.D.	N.D.	
PT12-REC	797	B02REC	N.D.	Blood	P2	N.D.	N.D.	N.D.		90	1209	N.D.	HLA-DR	N.D.	
PT12-REC	798	C11REC	N.D.	Blood	P2	N.D.	N.D.	N.D.		94	1165	N.D.	N.D.	N.D.	
PT12-REC	799	E03REC	N.D.	Blood	P2	N.D.	N.D.	N.D.		86	1184	N.D.	N.D.	N.D.	
PT12-REC	800	G05REC	N.D.	Blood	P2	N.D.	N.D.	N.D.		122	2036	N.D.	HLA-DR	N.D.	
PT12-REC	801	H07REC	N.D.	Blood	P2	N.D.	N.D.	N.D.		94	1485	N.D.	N.D.	N.D.	
PT12-REC	802	H08REC	N.D.	Blood	P2	N.D.	N.D.	N.D.		98	1297	N.D.	HLA-DR	N.D.	
PT14-REC	803	B05REC	CD4_136	Blood	P2	CASSLAYEQYF	TRBV7-8	33		887	7896	N.D.	N.D.	no	CLUSTER_1
PT14-REC	804	D03REC	CD4_137	Blood	P2	CASSLEAGYTGELFF	TRBV5-1	45		143	3261	16-30	N.D.	no	
PT14-REC	805	A03REC	CD4_137	Blood	P2	CASSLEAGYTGELFF	TRBV5-1	45		400	16149	N.D.	N.D.	no	
PT14-REC	806	A42REC	CD4_137	Blood	P2	CASSLEAGYTGELFF	TRBV5-1	45		195	3561	N.D.	N.D.	no	
PT14-REC	807	A05REC	CD4_137	Blood	P2	CASSLEAGYTGELFF	TRBV5-1	45		283	3479	N.D.	N.D.	no	
PT14-REC	808	A06REC	CD4_137	Blood	P2	CASSLEAGYTGELFF	TRBV5-1	45		143	2215	N.D.	N.D.	no	
PT14-REC	809	B01REC	CD4_137	Blood	P2	CASSLEAGYTGELFF	TRBV5-1	45		146	3351	N.D.	N.D.	no	
PT14-REC	810	B03REC	CD4_137	Blood	P2	CASSLEAGYTGELFF	TRBV5-1	45		887	7896	N.D.	N.D.	no	
PT14-REC	811	C05REC	CD4_137	Blood	P2	CASSLEAGYTGELFF	TRBV5-1	45		138	2804	N.D.	N.D.	no	
PT14-REC	812	D01REC	CD4_137	Blood	P2	CASSLEAGYTGELFF	TRBV5-1	45		154	4183	N.D.	N.D.	no	
PT14-REC	813	D04REC	CD4_137	Blood	P2	CASSLEAGYTGELFF	TRBV5-1	45		246	7823	N.D.	N.D.	no	
PT14-REC	814	E01REC	CD4_137	Blood	P2	CASSLEAGYTGELFF	TRBV5-1	45		236	2153	N.D.	N.D.	no	
PT14-REC	815	E02REC	CD4_137	Blood	P2	CASSLEAGYTGELFF	TRBV5-1	45		102	2675	N.D.	N.D.	no	
PT14-REC	816	E03REC	CD4_137	Blood	P2	CASSLEAGYTGELFF	TRBV5-1	45		102	2675	N.D.	N.D.	no	
PT14-REC	817	E04REC	CD4_137	Blood	P2	CASSLEAGYTGELFF	TRBV5-1	45		129	5872	N.D.	N.D.	no	
PT14-REC	818	F04REC	CD4_137	Blood	P2	CASSLEAGYTGELFF	TRBV5-1	45		115	2670	N.D.	N.D.	no	
PT14-REC	819	F05REC	CD4_137	Blood	P2	CASSLEAGYTGELFF	TRBV5-1	45		108	2080	N.D.	N.D.	no	
PT14-REC	820	G01REC	CD4_137	Blood	P2	CASSLEAGYTGELFF	TRBV5-1	45		173	5311	N.D.	N.D.	no	
PT14-REC	821	G02REC	CD4_137	Blood	P2	CASSLEAGYTGELFF	TRBV5-1	45		282	7653	N.D.	N.D.	no	
PT14-REC	822	G03REC	CD4_137	Blood	P2	CASSLEAGYTGELFF	TRBV5-1	45		124	1805	N.D.	N.D.	no	
PT14-REC	823	G05REC	CD4_137	Blood	P2	CASSLEAGYTGELFF	TRBV5-1	45		171	5090	N.D.	N.D.	no	
PT14-REC	824	H02REC	CD4_137	Blood	P2	CASSLEAGYTGELFF	TRBV5-1	45		286	1884	N.D.	N.D.	no	
PT14-REC	825	H03REC	CD4_137	Blood	P2	CASSLEAGYTGELFF	TRBV5-1	45		194	2399	N.D.	N.D.	no	
PT14-REC	826	H05REC	CD4_137	Blood	P2	CASSLEAGYTGELFF	TRBV5-1	45		194	4105	N.D.	N.D.	no	
PT14-REC	827	C06REC	CD4_137	Blood	P2	CASSLEAGYTGELFF	TRBV5-1	45		96	6548	N.D.	N.D.	no	
PT14-REC	828	F03REC	CD4_137	Blood	P2	CASSLEAGYTGELFF	TRBV5-1	45		168	7711	16-30	HLA-DR	no	
PT14-REC	829	D01REC	CD4_138	Blood	P2	CASSQVRQGASNTGELFF	TRBV4-3	54		142	4484	16-30	HLA-DR	no	
PT14-REC	830	A02REC	CD4_139	Blood	P2	CASSVGPSQETQYF	TRBV9	42		112	2137	N.D.	N.D.	no	
PT14-REC	831	B04REC	CD4_140	Blood	P2	CAWRGTGAEAFF	TRBV30	36		173	1941	31-45	HLA-DR	no	
PT14-REC	832	B06REC	N.D.	Blood	P2	N.D.	N.D.	N.D.		209	1889	N.D.	N.D.	N.D.	
PT1-REC	833	E10REC	CD4_141	Blood	PMP22	CASSDSESIGEKLFF	TRBV7-3	39		118	1064	86-100	HLA-DP	no	
PT5-REC	834	D04REC	CD4_142	Blood	PMP22	CASKPGQPNEKLFF	TRBV6-6	36		399	15685	81-100	N.D.	no	
PT5-REC	835	F05REC	CD4_143	Blood	PMP22	CASSLATGSNQPQHF	TRBV12-3	39		318	22889	126-140	HLA-DQ	no	
PT5-REC	836	G08REC	CD4_143	Blood	PMP22	CASSLATGSNQPQHF	TRBV12-3	39		219	8144	126-140	HLA-DQ	no	
PT5-REC	837	H02REC	CD4_143	Blood	PMP22	CASSLATGSNQPQHF	TRBV12-3	39		222	13587	N.D.	HLA-DQ	no	
PT5-REC	838	H09REC	CD4_143	Blood	PMP22	CASSLATGSNQPQHF	TRBV12-3	39		199	7178	126-140	HLA-DQ	no	
PT5-REC	839	D06REC	CD4_143	Blood	PMP22	CASSLATGSNQPQHF	TRBV12-3	39		265	22921	126-140	HLA-DQ	no	
PT5-REC	840	A08REC	CD4_144	Blood	PMP22	CASSLGRTGNEKLFF	TRBV7-9	39		351	32672	N.D.	N.D.	no	
PT5-REC	841	A10REC	CD4_144	Blood	PMP22	CASSLGRTGNEKLFF	TRBV7-9	39		471	18691	N.D.	N.D.	no	
PT5-REC	842	B04REC	CD4_144	Blood	PMP22	CASSLGRTGNEKLFF	TRBV7-9	39		213	7932	N.D.	N.D.	no	
PT5-REC	843	E07REC	CD4_144	Blood	PMP22	CASSLGRTGNEKLFF	TRBV7-9	39		216	9569	N.D.	N.D.	no	
PT5-REC	844	E08REC	CD4_144	Blood	PMP22	CASSLGRTGNEKLFF	TRBV7-9	39		271	23020	N.D.	N.D.	no	
PT5-REC	845	E10REC	CD4_144	Blood	PMP22	CASSLGRTGNEKLFF	TRBV7-9	39		395	13540	N.D.	N.D.	no	
PT5-REC	846	F06REC	CD4_144	Blood	PMP22	CASSLGRTGNEKLFF	TRBV7-9	39		212	7330	81-100	HLA-DR	no	
PT5-REC	847	G01REC	CD4_144	Blood	PMP22	CASSLGRTGNEKLFF	TRBV7-9	39		383	7552	81-100	HLA-DR	no	
PT5-REC	848	G04REC	CD4_144	Blood	PMP22	CASSLGRTGNEKLFF	TRBV7-9	39		234	18178	81-100	HLA-DR	no	
PT5-REC	849	F04REC	CD4_144	Blood	PMP22	CASSLGRTGNEKLFF	TRBV7-9	39		371	16888	81-100	HLA-DR	yes	
PT5-REC	850	A04REC	CD4_145	Blood	PMP22	CASSLITQRQETQYF	TRBV27	39		382	34528	N.D.	N.D.	no	
PT5-REC	851	A12REC	CD4_145	Blood	PMP22	CASSLITQRQETQYF	TRBV27	39		361	33395	N.D.	N.D.	no	
PT5-REC	852	B01REC	CD4_145	Blood	PMP22	CASSLITQRQETQYF	TRBV27	39		316	33628	N.D.	N.D.	no	
PT5-REC	853	B11REC	CD4_145	Blood	PMP22	CASSLITQRQETQYF	TRBV27	39		298	26173	81-100	HLA-DR	no	
PT5-REC	854	C01REC	CD4_145	Blood	PMP22	CASSLITQRQETQYF	TRBV27	39		284	34109	N.D.	N.D.	no	
PT5-REC	855	C03REC	CD4_145	Blood	PMP22	CASSLITQRQETQYF	TRBV27	39		370	27332	N.D.	N.D.	no	
PT5-REC	856	C09REC	CD4_145	Blood	PMP22	CASSLITQRQETQYF	TRBV27	39		290	26155	N.D.	N.D.	no	
PT5-REC	857	D07REC	CD4_145	Blood	PMP22	CASSLITQRQETQYF	TRBV27	39		287	25884	81-100	HLA-DR	no	
PT5-REC	858	D09REC	CD4_145	Blood	PMP22	CASSLITQRQETQYF	TRBV27	39		255	23429	N.D.	N.D.	no	
PT5-REC	859	E01REC	CD4_145	Blood	PMP22	CASSLITQRQETQYF	TRBV27	39		215	20670	N.D.	N.D.	no	
PT5-REC	860	E12REC	CD4_145	Blood	PMP22	CASSLITQRQETQYF	TRBV27	39		236	23298	N.D.	N.D.	no	
PT5-REC	861	H01REC	CD4_145	Blood	PMP22	CASSLITQRQETQYF	TRBV27	39		517	28832	81-100	HLA-DR	no	
PT5-REC	862	B07REC	CD4_145	Blood	PMP22	CASSLITQRQETQYF	TRBV27	39		339	21620	81-100	HLA-DR	no	
PT5-REC	863	E11REC	CD4_146	Blood	PMP22	CASSLSGGNSPLHF	TRBV28	32		257	31307	21-35	N.D.	no	
PT5-REC	864	C07REC	CD4_147	Blood	PMP22	CASSVVGTPGADTQYF	TRBV9	42		198	2478	N.D.	N.D.	no	
PT5-REC	865	G06REC	CD4_147	Blood	PMP22	CASSVVGTPGADTQYF	TRBV9	42		242	6693	21-35	HLA-DR	no	
PT5-REC	866	B06REC	CD4_147	Blood	PMP22	CASSVVGTPGADTQYF	TRBV9	42		270	57165	21-35	HLA-DR	no	
PT5-AC	867	A01AC	N.D.	Blood	PMP22	N.D.	N.D.	N.D.		197	4069	56-70	HLA-DR	N.D.	
PT5-AC	868	D10AC	N.D.	Blood	PMP22	N.D.	N.D.	N.D.		350	5409	N.D.	HLA-DR	N.D.	
PT5-AC	869	G01AC	N.D.	Blood	PMP22	N.D.	N.D.	N.D.		186	1389	N.D.	HLA-DR	N.D.	
PT5-AC	870	H01AC	N.D.	Blood	PMP22	N.D.	N.D.	N.D.		254	1102	N.D.	N.D.	N.D.	
PT5-REC	871	F03REC	N.D.	Blood	PMP22	N.D.	N.D.	N.D.		292	35856	N.D.	N.D.	N.D.	
PT5-REC	872	D03REC	N.D.	Blood	PMP22	N.D.	N.D.	N.D.		923	14212	N.D.	N.D.	N.D.	
PT5-REC	873	G07REC	N.D.	Blood	PMP22	N.D.	N.D.	N.D.		271	28862	N.D.	HLA-DR	N.D.	
PT5-REC	874	G10REC	N.D.	Blood	PMP22	N.D.	N.D.	N.D.		203	17136	N.D.	HLA-DR	N.D.	
PT5-REC	875	H06REC	N.D.	Blood	PMP22	N.D.	N.D.	N.D.		430	25519	N.D.	HLA-DR	N.D.	
PT6-REC	876	A11REC	CD4_148	Blood	PMP22	CSARSSYNEQFF	TRBV20	30		165	9676	N.D.	N.D.	no	
PT6-REC	877	C11REC	CD4_148	Blood	PMP22	CSARSSYNEQFF	TRBV20	30		149	4473	N.D.	N.D.	no	
PT6-REC	878	E03REC	CD4_148	Blood	PMP22	CSARSSYNEQFF	TRBV20	30		178	2276	N.D.	N.D.	no	
PT6-REC	879	E04REC	CD4_148	Blood	PMP22	CSARSSYNEQFF	TRBV20	30		122	1957	N.D.	N.D.	no	
PT6-REC	880	F11REC	CD4_148	Blood	PMP22	CSARSSYNEQFF	TRBV20	30		240	1642	N.D.	HLA-DQ	no	
PT6-REC	881	G01REC	CD4_148	Blood	PMP22	CSARSSYNEQFF	TRBV20	30		226	3020	N.D.	N.D.	no	
PT6-REC	882	G09REC	CD4_148	Blood	PMP22	CSARSSYNEQFF	TRBV20	30		191	2492	N.D.	HLA-DQ	no	
PT6-REC	883	H02REC	CD4_148	Blood	PMP22	CSARSSYNEQFF	TRBV20	30		133	17754	N.D.	N.D.	no	
PT6-REC	884	F09REC	CD4_148	Blood	PMP22	CSARSSYNEQFF	TRBV20	30		164	7589	91-105	HLA-DQ	no	
PT7-AC	885	CL34AC	CD4_149	Blood	PMP22	CSAPAGGGSPLHF	TRBV20	33		73	1883	N.D.	N.D.	yes	CLUSTER_12
PT7-AC	886	CL59AC	CD4_149	Blood	PMP22	CSAPAGGGSPLHF	TRBV20	33		2323	16214	N.D.	HLA-DR	yes	CLUSTER_12
PT7-REC	887	CL22REC	CD4_150	Blood	PMP22	CASEAGTSGNTIYF	TRBV6-1	36		172	4911	N.D.	HLA-DR	no	
PT7-REC	888	CL11REC	CD4_150	Blood	PMP22	CASEAGTSGNTIYF	TRBV6-1	36		637	4641	N.D.	HLA-DR	no	
PT7-REC	889	CL23REC	CD4_150	Blood	PMP22	CASEAGTSGNTIYF	TRBV6-1	36		1129	5717	N.D.	HLA-DR	no	
PT7-REC	890	CL34REC	CD4_150	Blood	PMP22	CASEAGTSGNTIYF	TRBV6-1	36		1227	4599	N.D.	HLA-DR	no	
PT7-REC	891	CL47REC	CD4_150	Blood	PMP22	CASEAGTSGNTIYF	TRBV6-1	36		2091	7105	N.D.	HLA-DR	no	
PT7-REC	892	CL51REC	CD4_150	Blood	PMP22	CASEAGTSGNTIYF	TRBV6-1	36		2525	7835	N.D.	HLA-DR	no	
PT7-REC	893	CL55REC	CD4_150	Blood	PMP22	CASEAGTSGNTIYF	TRBV6-1	36		1155	6192	81-95	HLA-DR	no	
PT7-REC	894	CL58REC	CD4_150	Blood	PMP22	CASEAGTSGNTIYF	TRBV6-1	36		1607	5296	N.D.	HLA-DR	no	
PT7-REC	895	CL64REC	CD4_150	Blood	PMP22	CASEAGTSGNTIYF	TRBV6-1	36		2464	6105	N.D.	HLA-DR	no	
PT7-REC	896	CL68REC	CD4_150	Blood	PMP22	CASEAGTSGNTIYF	TRBV6-1	36		1717	7232	N.D.	HLA-DR	no	
PT7-REC	897	CL41REC	CD4_150	Blood	PMP22	CASEAGTSGNTIYF	TRBV6-1	36		642	9391	81-95	HLA-DR	no	
PT7-REC	898	CL95REC	CD4_151	Blood	PMP22	CASHTGTANTGELFF	TRBV19	39	in PT11	3755	12945	101-115 / 106-120	HLA-DR	no	
PT7-REC	899	CL23REC	CD4_151	Blood	PMP22	CASHTGTANTGELFF	TRBV19	39	in PT11	1410	7324	101-115 / 106-120	HLA-DR	yes	
PT7-REC	900	CL48REC	CD4_152	Blood	PMP22	CASSERGGALNTEAFF	TRBV10-2	42		335	4731	141-155	HLA-DR	no	
PT7-REC	901	CL105REC	CD4_153	Blood	PMP22	CASSGGQGVNQPQHF	TRBV6-6	39		814	10680	106-120	HLA-DR	no	
PT7-REC	902	CL16REC	CD4_154	Blood	PMP22	CASSGLGVGELFF	TRBV27	33		1361	4990	56-70	HLA-DR	yes	
PT7-REC	903	CL33REC	CD4_155	Blood	PMP22	CASSLEGGRTQYF	TRBV7-6	33		12	5000	106-120	HLA-DR	no	
PT7-REC	904	CL61REC	CD4_156	Blood	PMP22	CASSLEGSREQYF	TRBV7-6	33		303	7709	N.D.	HLA-DR	no	
PT7-REC	905	CL42REC	CD4_156	Blood	PMP22	CASSLEGSREQYF	TRBV7-6	33		3392	14415	106-120	HLA-DR	no	
PT7-REC	906	CL106REC	CD4_156	Blood	PMP22	CASSLEGSREQYF	TRBV7-6	33		2256	15306	N.D.	HLA-DR	no	
PT7-REC	907	CL11REC	CD4_156	Blood	PMP22	CASSLEGSREQYF	TRBV7-6	33		2506	15351	N.D.	HLA-DR	no	
PT7-REC	908	CL75REC	CD4_156	Blood	PMP22	CASSLEGSREQYF	TRBV7-6	33		3617	16758	106-120	HLA-DR	no	

PT7-REC	909	CL53REC	CD4_157	Blood	PMP22	CASSRSWDSGYEQYF	TRBV4-3	39		684	4176	81-95	HLA-DR	no	
PT7-REC	910	CL10REC	CD4_158	Blood	PMP22	CASSSGLAGALTQYF	TRBV6-2	39		2229	8512	N.D.	HLA-DR	no	
PT7-REC	911	CL07REC	CD4_158	Blood	PMP22	CASSSGLAGALTQYF	TRBV6-2	39		1598	8525	N.D.	HLA-DR	no	
PT7-REC	912	CL05REC	CD4_158	Blood	PMP22	CASSSGLAGALTQYF	TRBV6-2	39		4475	16158	N.D.	HLA-DR	no	
PT7-REC	913	CL08REC	CD4_158	Blood	PMP22	CASSSGLAGALTQYF	TRBV6-2	39		231	5946	N.D.	HLA-DR	no	
PT7-REC	914	CL12REC	CD4_158	Blood	PMP22	CASSSGLAGALTQYF	TRBV6-2	39		2538	12487	N.D.	HLA-DR	no	
PT7-REC	915	CL16REC	CD4_158	Blood	PMP22	CASSSGLAGALTQYF	TRBV6-2	39		1627	7447	N.D.	HLA-DR	no	
PT7-REC	916	CL20REC	CD4_158	Blood	PMP22	CASSSGLAGALTQYF	TRBV6-2	39		2432	7857	N.D.	HLA-DR	no	
PT7-REC	917	CL34REC	CD4_158	Blood	PMP22	CASSSGLAGALTQYF	TRBV6-2	39		16	9066	N.D.	HLA-DR	no	
PT7-REC	918	CL38REC	CD4_158	Blood	PMP22	CASSSGLAGALTQYF	TRBV6-2	39		1267	6057	N.D.	HLA-DR	no	
PT7-REC	919	CL43REC	CD4_158	Blood	PMP22	CASSSGLAGALTQYF	TRBV6-2	39		644	5220	N.D.	HLA-DR	no	
PT7-REC	920	CL47REC	CD4_158	Blood	PMP22	CASSSGLAGALTQYF	TRBV6-2	39		153	3763	N.D.	HLA-DR	no	
PT7-REC	921	CL48REC	CD4_158	Blood	PMP22	CASSSGLAGALTQYF	TRBV6-2	39		235	12137	N.D.	HLA-DR	no	
PT7-REC	922	CL50REC	CD4_158	Blood	PMP22	CASSSGLAGALTQYF	TRBV6-2	39		927	7258	N.D.	HLA-DR	no	
PT7-REC	923	CL51REC	CD4_158	Blood	PMP22	CASSSGLAGALTQYF	TRBV6-2	39		2488	11718	N.D.	HLA-DR	no	
PT7-REC	924	CL52REC	CD4_158	Blood	PMP22	CASSSGLAGALTQYF	TRBV6-2	39		1671	8159	N.D.	HLA-DR	no	
PT7-REC	925	CL60REC	CD4_158	Blood	PMP22	CASSSGLAGALTQYF	TRBV6-2	39		1774	8098	N.D.	HLA-DR	no	
PT7-REC	926	CL68REC	CD4_158	Blood	PMP22	CASSSGLAGALTQYF	TRBV6-2	39		2034	7927	N.D.	HLA-DR	no	
PT7-REC	927	CL70REC	CD4_158	Blood	PMP22	CASSSGLAGALTQYF	TRBV6-2	39		1887	6033	N.D.	HLA-DR	no	
PT7-REC	928	CL72REC	CD4_158	Blood	PMP22	CASSSGLAGALTQYF	TRBV6-2	39		792	6055	N.D.	HLA-DR	no	
PT7-REC	929	CL79REC	CD4_158	Blood	PMP22	CASSSGLAGALTQYF	TRBV6-2	39		2445	11334	106-120	HLA-DR	no	
PT7-REC	930	CL83REC	CD4_158	Blood	PMP22	CASSSGLAGALTQYF	TRBV6-2	39		746	7400	N.D.	HLA-DR	no	
PT7-REC	931	CL88REC	CD4_158	Blood	PMP22	CASSSGLAGALTQYF	TRBV6-2	39		443	5145	N.D.	HLA-DR	no	
PT7-REC	932	CL92REC	CD4_158	Blood	PMP22	CASSSGLAGALTQYF	TRBV6-2	39		3251	11652	N.D.	HLA-DR	no	
PT7-REC	933	CL93REC	CD4_158	Blood	PMP22	CASSSGLAGALTQYF	TRBV6-2	39		154	4457	N.D.	HLA-DR	no	
PT7-REC	934	CL100REC	CD4_158	Blood	PMP22	CASSSGLAGALTQYF	TRBV6-2	39		128	3094	N.D.	HLA-DR	no	
PT7-REC	935	CL103REC	CD4_158	Blood	PMP22	CASSSGLAGALTQYF	TRBV6-2	39		1469	6530	N.D.	HLA-DR	no	
PT7-REC	936	CL31REC	CD4_158	Blood	PMP22	CASSSGLAGALTQYF	TRBV6-2	39		2178	8296	106-120	HLA-DR	yes	
PT7-AC	937	CL75AC	N.D.	Blood	PMP22	N.D.	N.D.	N.D.		2474	6069	N.D.	N.D.	N.D.	
PT7-REC	938	CL35REC	N.D.	Blood	PMP22	N.D.	N.D.	N.D.		152	6299	N.D.	N.D.	N.D.	
PT7-REC	939	CL36REC	N.D.	Blood	PMP22	N.D.	N.D.	N.D.		178	2594	N.D.	N.D.	N.D.	
PT7-REC	940	CL04REC	N.D.	Blood	PMP22	N.D.	N.D.	N.D.		3250	9792	N.D.	HLA-DR	N.D.	
PT7-REC	941	CL41REC	N.D.	Blood	PMP22	N.D.	N.D.	N.D.		1502	8061	N.D.	HLA-DR	N.D.	
PT7-REC	942	CL63REC	N.D.	Blood	PMP22	N.D.	N.D.	N.D.		3495	11824	N.D.	HLA-DR	N.D.	
PT7-REC	943	CL76REC	N.D.	Blood	PMP22	N.D.	N.D.	N.D.		229	4664	N.D.	HLA-DR	N.D.	
PT8-AC	944	A08AC	CD4_159	Blood	PMP22	CASRSRKLGPTSRLY	TRBV19	24		1061	10629	N.D.	HLA-DR	no	
PT8-AC	945	A09AC	CD4_160	Blood	PMP22	CASSKQEAGAKVLTF	TRBV19	39		1142	11359	106-120	HLA-DR	no	
PT8-AC	946	D01AC	CD4_161	Blood	PMP22	CASSKQEAGANVLTF	TRBV19	39		1286	3839	106-120	HLA-DR	no	
PT8-AC	947	E03AC	CD4_162	Blood	PMP22	CAWSVRGGAYSEAFF	TRBV30	38		3084	14423	91-105	N.D.	no	
PT8-REC	948	B11REC	CD4_163	Blood	PMP22	CASITGGAVSQYF	TRBV6-1	33		1180	3093	N.D.	N.D.	no	
PT8-REC	949	C08REC	CD4_164	Blood	PMP22	CASTRGAAKGSSFF	TRBV2	36		1211	2980	N.D.	N.D.	no	
PT8-AC	950	C10AC	N.D.	Blood	PMP22	N.D.	N.D.	N.D.		2897	8240	105-120	N.D.	N.D.	
PT8-AC	951	E02AC	N.D.	Blood	PMP22	N.D.	N.D.	N.D.		3770	8782	105-120	N.D.	N.D.	
PT8-AC	952	E03AC	N.D.	Blood	PMP22	N.D.	N.D.	N.D.		3084	14423	91-105	N.D.	N.D.	
PT8-AC	953	C03AC	N.D.	Blood	PMP22	N.D.	N.D.	N.D.		755	17836	N.D.	N.D.	N.D.	
PT8-AC	954	C06AC	N.D.	Blood	PMP22	N.D.	N.D.	N.D.		798	8058	N.D.	N.D.	N.D.	
PT8-AC	955	D05AC	N.D.	Blood	PMP22	N.D.	N.D.	N.D.		787	10647	N.D.	N.D.	N.D.	
PT8-AC	956	F02AC	N.D.	Blood	PMP22	N.D.	N.D.	N.D.		1416	6560	N.D.	N.D.	N.D.	
PT8-AC	957	B02AC	N.D.	Blood	PMP22	N.D.	N.D.	N.D.		1666	13265	105-120	N.D.	N.D.	
PT8-AC	958	B03AC	N.D.	Blood	PMP22	N.D.	N.D.	N.D.		3095	8530	91-105	N.D.	N.D.	
PT8-REC	959	C08REC	N.D.	Blood	PMP22	N.D.	N.D.	N.D.		1211	2980	N.D.	N.D.	N.D.	
PT13-REC	960	B09REC	N.D.	Blood	PMP22	N.D.	N.D.	N.D.		229	1017	N.D.	N.D.	N.D.	
PT14-AC	961	C08AC	CD4_136	Blood	PMP22	CASSLAYEQYF	TRBV7-8	33		98	8441	N.D.	N.D.	no	CLUSTER_1
PT14-AC	962	C06AC	CD4_165	Blood	PMP22	CASSLEGGREQFF	TRBV7-6	39		84	2377	N.D.	N.D.	no	
PT14-AC	963	B08AC	CD4_166	Blood	PMP22	CSVVLARGADTQYF	TRBV29	42		116	4086	N.D.	N.D.	no	
PT14-AC	964	B09AC	CD4_166	Blood	PMP22	CSVVLARGADTQYF	TRBV29	42		113	4990	N.D.	N.D.	no	
PT14-AC	965	C07AC	CD4_166	Blood	PMP22	CSVVLARGADTQYF	TRBV29	42		118	16680	N.D.	N.D.	no	
PT14-AC	966	C09AC	CD4_166	Blood	PMP22	CSVVLARGADTQYF	TRBV29	42		123	19241	N.D.	N.D.	no	
PT14-AC	967	D06AC	CD4_166	Blood	PMP22	CSVVLARGADTQYF	TRBV29	42		63	10083	N.D.	N.D.	no	
PT14-AC	968	D07AC	CD4_166	Blood	PMP22	CSVVLARGADTQYF	TRBV29	42		128	2779	N.D.	N.D.	no	
PT14-AC	969	D08AC	CD4_166	Blood	PMP22	CSVVLARGADTQYF	TRBV29	42		258	14001	N.D.	N.D.	no	
PT14-AC	970	E06AC	CD4_166	Blood	PMP22	CSVVLARGADTQYF	TRBV29	42		66	4660	N.D.	N.D.	no	
PT14-AC	971	E07AC	CD4_166	Blood	PMP22	CSVVLARGADTQYF	TRBV29	42		105	10861	N.D.	N.D.	no	
PT14-AC	972	E08AC	CD4_166	Blood	PMP22	CSVVLARGADTQYF	TRBV29	42		91	15932	N.D.	N.D.	no	
PT14-AC	973	F08AC	CD4_166	Blood	PMP22	CSVVLARGADTQYF	TRBV29	42		139	15189	N.D.	N.D.	no	
PT14-AC	974	G07AC	CD4_166	Blood	PMP22	CSVVLARGADTQYF	TRBV29	42		123	3990	N.D.	N.D.	no	
PT14-AC	975	G08AC	CD4_166	Blood	PMP22	CSVVLARGADTQYF	TRBV29	42		127	11568	N.D.	N.D.	no	
PT14-AC	976	H08AC	CD4_166	Blood	PMP22	CSVVLARGADTQYF	TRBV29	42		81	7841	N.D.	N.D.	no	
PT14-AC	977	H07AC	CD4_166	Blood	PMP22	CSVVLARGADTQYF	TRBV29	42		24	4826	N.D.	N.D.	no	
PT14-AC	978	B07AC	CD4_166	Blood	PMP22	CSVVLARGADTQYF	TRBV29	42		126	9409	N.D.	N.D.	no	
PT14-AC	979	A01AC	N.D.	Blood	PMP22	N.D.	N.D.	N.D.		205	5035	N.D.	N.D.	N.D.	
PT14-AC	980	A03AC	N.D.	Blood	PMP22	N.D.	N.D.	N.D.		122	2392	N.D.	N.D.	N.D.	
PT14-AC	981	A08AC	N.D.	Blood	PMP22	N.D.	N.D.	N.D.		158	14593	N.D.	N.D.	N.D.	
PT14-AC	982	A09AC	N.D.	Blood	PMP22	N.D.	N.D.	N.D.		209	11629	N.D.	N.D.	N.D.	
PT14-AC	983	B02AC	N.D.	Blood	PMP22	N.D.	N.D.	N.D.		132	3205	N.D.	N.D.	N.D.	
PT14-AC	984	C01AC	N.D.	Blood	PMP22	N.D.	N.D.	N.D.		112	5081	N.D.	N.D.	N.D.	
PT14-AC	985	E02AC	N.D.	Blood	PMP22	N.D.	N.D.	N.D.		157	3447	N.D.	N.D.	N.D.	
PT14-AC	986	F07AC	N.D.	Blood	PMP22	N.D.	N.D.	N.D.		114	19125	N.D.	N.D.	N.D.	
PT14-AC	987	G01AC	N.D.	Blood	PMP22	N.D.	N.D.	N.D.		130	2617	N.D.	N.D.	N.D.	
PT5-REC	988	E10CD8	CD8_1	Blood	P0	CSVLGGYGELFF	TRBV29	38		200	2197	146-160	class I	N.D.	
PT5-REC	989	B09CD8	CD8_2	Blood	P0	CASSQQTGGLNTEAFF	TRBV29	42		202	1542	N.D.	class I	N.D.	
PT6-AC	990	A01AC	CD8_3	Blood	P0	CASKIGLGAYGYTF	TRBV6-5			70	1704	176-195	class I	N.D.	
PT6-AC	991	B03AC	CD8_3	Blood	P0	CASKIGLGAYGYTF	TRBV6-5			25	2636	176-195	N.D.	N.D.	
PT6-AC	992	B05AC	CD8_3	Blood	P0	CASKIGLGAYGYTF	TRBV6-5			37	1304	176-195	N.D.	N.D.	
PT6-AC	993	D04AC	N.D.	Blood	P0	N.D.	N.D.	N.D.		84	7164	N.D.	N.D.	N.D.	
PT6-AC	994	D05AC	N.D.	Blood	P0	N.D.	N.D.	N.D.		34	1921	N.D.	N.D.	N.D.	
PT7-REC	995	H07REC	CD8_4	Blood	P0	CASSATLRGDTEAFF	TRBV19	39		374	3788	N.D.	N.D.	N.D.	
PT7-REC	996	A01REC	CD8_5	Blood	P2	CASTHGRGYTEAFF	TRBV4-2	36		491	2357	N.D.	N.D.	N.D.	
PT7-REC	997	A02REC	N.D.	Blood	P2	N.D.	N.D.	N.D.		559	7628	N.D.	N.D.	N.D.	
PT7-REC	998	D02REC	N.D.	Blood	P2	N.D.	N.D.	N.D.		161	4537	N.D.	N.D.	N.D.	
PT9-REC	999	H04REC	CD8_6	Blood	P2	CASRQGPRTDTQYF	TRBV19	42		270	2298	N.D.	N.D.	N.D.	
PT9-REC	1000	C09REC	CD8_7	Blood	P2	CASSLAGTTRELFF	TRBV7-8	42		982	2763	N.D.	N.D.	N.D.	
PT9-REC	1001	A03REC	CD8_8	Blood	P2	CASSQGANNEQFF	TRBV7-9	39		1499	4737	N.D.	N.D.	N.D.	
PT9-REC	1002	B05REC	CD8_9	Blood	P2	CASSRASGQLNEQFF	TRBV19	45		7529	32610	N.D.	N.D.	N.D.	
PT9-REC	1003	H06REC	CD8_9	Blood	P2	CASSRASGQLNEQFF	TRBV19	45		934	16848	N.D.	N.D.	N.D.	
PT9-REC	1004	B09REC	CD8_10	Blood	P2	CASSRGTDFNYGYTF	TRBV7-9	45		953	3620	N.D.	N.D.	N.D.	
PT9-REC	1005	H02REC	CD8_11	Blood	P2	CAWSAINRYTDTQYF	TRBV30	45		1094	7427	N.D.	N.D.	N.D.	
PT9-REC	1006	C10REC	N.D.	Blood	P2	N.D.	N.D.	N.D.		903	3765	N.D.	N.D.	N.D.	
PT9-REC	1007	E01REC	N.D.	Blood	P2	N.D.	N.D.	N.D.		2309	10243	N.D.	N.D.	N.D.	
PT9-REC	1008	H01REC	N.D.	Blood	P2	N.D.	N.D.	N.D.		109	2350	N.D.	N.D.	N.D.	
PT9-REC	1009	H09REC	N.D.	Blood	P2	N.D.	N.D.	N.D.		114	6859	N.D.	N.D.	N.D.	
PT6-REC	1010	F5REC	CD8_12	Blood	PMP22	CTSSPDWETQYF	TRBV18	30		40	4235	N.D.	N.D.	N.D.	
PT6-REC	1011	C5REC	N.D.	Blood	PMP22	N.D.	N.D.	N.D.		66	4001	N.D.	N.D.	N.D.	
PT6-REC	1012	F9REC	N.D.	Blood	PMP22	N.D.	N.D.	N.D.		30	1461	N.D.	N.D.	N.D.	
PT7-REC	1013	A07REC	CD8_13	Blood	PMP22	CASRILGDTQYF	TRBV28	30		1194	23808	145-160	N.D.	N.D.	
PT7-REC	1014	H01REC	CD8_13	Blood	PMP22	CASRILGDTQYF	TRBV28	30		900	20777	N.D.	N.D.	N.D.	
PT7-REC	1015	C01REC	CD8_13	Blood	PMP22	CASRILGDTQYF	TRBV28	30		3016	17526	N.D.	N.D.	N.D.	
PT7-REC	1016	C08REC	CD8_13	Blood	PMP22	CASRILGDTQYF	TRBV28	30		1088	7101	N.D.	N.D.	N.D.	
PT7-REC	1017	D09REC	CD8_13	Blood	PMP22	CASRILGDTQYF	TRBV28	30		459	10261	N.D.	N.D.	N.D.	
PT7-REC	1018	G12REC	CD8_14	Blood	PMP22	CASSQGRGSTEAFF	TRBV4-2	36		16	3384	N.D.	N.D.	N.D.	
PT7-REC	1019	C02REC	N.D.	Blood	PMP22	N.D.	N.D.	N.D.		950	6489	N.D.	N.D.	N.D.	
PT7-REC	1020	F11REC	N.D.	Blood	PMP22	N.D.	N.D.	N.D.		5116	19480	N.D.	N.D.	N.D.	
PT8-REC	1021	A08REC	N.D.	Blood	PMP22	N.D.	N.D.	N.D.		1113	2733	N.D.	N.D.	N.D.	
PT9-REC	1022	B05REC	CD8_15	Blood	PMP22	CASSLGGAGTEAFF	TRBV7-3	36		298	5687	N.D.	N.D.	N.D.	
PT9-REC	1023	G01REC	CD8_15	Blood	PMP22	CASSLGGAGTEAFF	TRBV7-3	36		1384	3277	N.D.	N.D.	N.D.	
PT9-REC	1024	A05REC	CD8_16	Blood	PMP22	CASSYPTGGLMNTEAFF	TRBV6-2	45		763	3567	N.D.	N.D.	N.D.	
PT9-REC	1025	C08REC	CD8_16	Blood	PMP22	CASSYPTGGLMNTEAFF	TRBV6-2	45		615	2854	N.D.	N.D.	N.D.	
PT9-REC	1026	D01REC	CD8_16	Blood	PMP22	CASSYPTGGLMNTEAFF	TRBV6-2	45		1127	3735	N.D.	N.D.	N.D.	
PT9-REC	1027	G02REC	CD8_16	Blood	PMP22	CASSYPTGGLMNTEAFF	TRBV6-2	45		542	4310	N.D.	N.D.	N.D.	
PT9-REC	1028	H12REC	CD8_16	Blood	PMP22	CASSYPTGGLMNTEAFF	TRBV6-2	45		1513	9926	N.D.	N.D.	N.D.	
PT9-REC	1029	A01REC	N.D.	Blood	PMP22	N.D.	N.D.	N.D.		408	2332	N.D.	N.D.	N.D.	
PT14-AC	1030	A01AC	CD8_17	Blood	PMP22	CASSLRTGELFF	TRBV27	36		133	1001	N.D.	N.D.	N.D.	
PT14-AC	1031	B02AC	CD8_17	Blood	PMP22	CASSLRTGELFF	TRBV27	36		111	1016	N.D.	N.D.	N.D.	
PT14-AC	1032	A08AC	CD8_17	Blood	PMP22	CASSLRTGELFF	TRBV27	36		118	2009	N.D.	N.D.	N.D.	
PT14-AC	1033	A09AC	CD8_17	Blood	PMP22	CASSLRTGELFF	TRBV27	36		128	2041	N.D.	N.D.	N.D.	
PT14-AC	1034	B06AC	CD8_17	Blood	PMP22	CASSLRTGELFF	TRBV27	36		84	929	N.D.	N.D.	N.D.	
PT14-AC	1035	B09AC	CD8_17	Blood	PMP22	CASSLRTGELFF	TRBV27	36		175	1992	N.D.	N.D.	N.D.	
PT14-AC	1036	B11AC	CD8_17	Blood	PMP22	CASSLRTGELFF	TRBV27	36		115	3818	N.D.	N.D.	N.D.	
PT14-AC	1037	C11AC	CD8_17	Blood	PMP22	CASSLRTGELFF	TRBV27	36		106	2266	N.D.	N.D.	N.D.	
PT14-AC	1038	D05AC	CD8_17	Blood	PMP22	CASSLRTGELFF	TRBV27	36		56	1862	N.D.	N.D.	N.D.	

PT14-AC	1039	D12AC	CD8_17	Blood	PMP22	CASSLRTGELFF	TRBV27	36		212	4642	N.D.	N.D.	N.D.	
PT14-AC	1040	F04AC	CD8_17	Blood	PMP22	CASSLRTGELFF	TRBV27	36		73	1173	N.D.	N.D.	N.D.	
PT14-AC	1041	G08AC	CD8_17	Blood	PMP22	CASSLRTGELFF	TRBV27	36		127	1402	N.D.	N.D.	N.D.	
PT14-AC	1042	H09AC	CD8_17	Blood	PMP22	CASSLRTGELFF	TRBV27	36		120	1802	N.D.	N.D.	N.D.	
PT2-REC	1043	B03REC3	N.D.	Blood	CMV-P0/P2	N.D.	N.D.	N.D.		192	2414	N.D.	N.D.	N.D.	
PT2-REC	1044	A02REC3	N.D.	Blood	CMV-P0/P2	N.D.	N.D.	N.D.		199	2216	N.D.	N.D.	N.D.	
PT2-REC	1045	G01REC3	CD4_167	Blood	CMV-P0/P2	CASSQVSAQGLGYGYTF	TRBV4-3	51		365	2439	N.D.	N.D.	no	
PT2-REC	1046	B08REC3	CD4_168	Blood	CMV	CASSEQDSGNTIYF	TRBV10-2	42		140	2064	N.D.	N.D.	no	
PT2-REC	1047	B01REC3	CD4_169	Blood	CMV	CASSLTSDYGYTF	TRBV12-3	39		150	1355	N.D.	N.D.	no	
PT2-REC	1048	F12REC1	N.D.	Blood	CMV	N.D.	N.D.	N.D.		150	1548	N.D.	N.D.	N.D.	

N.D. = not determined

Supplementary Table 3

Frequencies of CDR3β lengths. Frequency of *TCRB* complementarity-determining region 3 (CDR3β) length of clonotypes reactive to PNS-myelin antigens (n = 166), SARS-CoV-2 antigens from post-COVID-19 GBS patients (n = 92) as well as TCRβ clonotypes identified in microbe-reactive CFSE^{low} fractions from HD. The original data sources are indicated.

Dataset Name	Dataset ID	Source	Clonotype (n)	Freq of CDR3β nucleotide length (%)																												
				3 nt	6 nt	9 nt	12 nt	15 nt	18 nt	21 nt	24 nt	27 nt	30 nt	33 nt	36 nt	39 nt	42 nt	45 nt	48 nt	51 nt	54 nt	57 nt	60 nt	63 nt	66 nt	69 nt	72 nt	75 nt	78 nt	81 nt	84 nt	87 nt
PT1_AC	PT1	current manuscript	72777	0	0	0	0	0.00137	0	0.00275	0.00962	0.02748	0.02336	0.07695	0.37237	3.17133	7.42542	16.682468	23.4154	24.3126	13.6018	6.17503	2.84568	1.19268	0.42321	0.15389	0.06321	0.01924	0.00137	0.00137	0.00137	0
PT5 AC	PT5	current manuscript	63248	0	0	0	0	0.00158	0	0.00316	0.01107	0.01739	0.03004	0.07905	0.4095	2.78744	7.51644	17.07722	23.0695	24.3502	13.6494	6.0998	2.86807	1.29016	0.49962	0.16127	0.04901	0.01581	0.00949	0.00474	0	0
PT7_AC	PT7	current manuscript	66244	0	0	0	0	0	0	0.00755	0.00302	0.02415	0.04529	0.05736	0.37437	2.91045	7.25802	16.821146	23.5946	23.9358	13.965	6.28887	2.80176	1.14425	0.47853	0.17813	0.06491	0.0317	0.01359	0.00151	0	0
PT10_AC	PT10	current manuscript	1639	0	0	0	0	0	0	0	0	0	0	0	0.30506	2.50153	7.80964	17.327639	23.3679	25.1373	12.3246	6.16229	3.17267	1.22026	0.36608	0.06101	0.12203	0.12203	0	0	0	0
PT11_AC	PT11	current manuscript	8607	0	0	0	0	0	0	0.02324	0	0	0.02324	0.08133	0.37179	2.76519	7.48228	16.405252	23.9456	23.7597	13.6401	6.20425	3.04403	1.49878	0.49959	0.17428	0.02324	0.04647	0.01162	0	0	0
PT12_AC	PT12	current manuscript	12572	0	0	0	0	0	0.00795	0.00795	0	0.01591	0.05568	0.09545	0.2784	2.79987	7.19854	15.725422	22.216	24.5068	13.8085	7.3099	3.6112	1.51129	0.51702	0.17499	0.1034	0.02386	0.00795	0.00795	0.00795	0.00795
narcolepsy_P2	A1	Latorre et al. 2018	40896	0	0	0	0	0	0.01223	0.00245	0.00734	0.01712	0.01467	0.06602	0.34967	3.22525	7.19386	16.683783	22.7724	24.2249	14.1799	6.51653	2.76311	1.20061	0.48905	0.18095	0.06358	0.03179	0.00245	0	0.00245	0
narcolepsy _P3	A2	Latorre et al. 2018	43981	0	0	0	0	0	0	0.00909	0.00227	0.01592	0.03638	0.0955	0.56615	3.4333	8.39453	17.171051	22.8258	22.7689	13.4194	6.29135	2.92172	1.26873	0.52523	0.14779	0.06594	0.02501	0.00682	0.00227	0.00682	0
narcolepsy _P4	A3	Latorre et al. 2018	27380	0	0	0	0.00365	0	0	0	0	0.01461	0.04383	0.04383	0.3981	2.68079	7.02703	16.563185	23.298	24.0248	13.477	6.84076	3.15194	1.40979	0.58437	0.25566	0.10957	0.04383	0.01826	0.00365	0.0073	0
narcolepsy _P5	A4	Latorre et al. 2018	15283	0	0	0	0.00365	0	0.00654	0	0	0.00654	0.01309	0.08506	0.22901	2.8463	7.11902	17.941504	23.2546	23.2284	13.9109	6.15062	3.06877	1.26284	0.52346	0.22247	0.06543	0.03926	0.01309	0.00654	0.00654	0
narcolepsy _P7	A5	Latorre et al. 2018	34738	0	0	0	0	0	0.00576	0.00864	0.01151	0.03742	0.0403	0.07485	0.51241	3.4717	8.21003	16.221429	22.6409	22.9576	13.6623	6.7275	3.12626	1.31556	0.58725	0.23605	0.08924	0.03742	0.01727	0.00864	0	0
narcolepsy_C10	C5	Latorre et al. 2018	34176	0	0	0	0	0	0	0.00878	0.00293	0.01756	0.02341	0.08778	0.37161	3.07526	7.3853	15.663038	23.2298	24.0988	14.2029	6.46945	3.09574	1.44838	0.54717	0.16093	0.06145	0.03511	0.00878	0.00585	0	0
narcolepsy_C11	C6	Latorre et al. 2018	38149	0	0	0	0	0	0.00524	0.00786	0	0.02097	0.01049	0.0734	0.38009	2.87295	7.25052	16.592833	23.1985	24.0583	13.8641	6.74723	2.95158	1.17172	0.53212	0.19135	0.0498	0.01049	0.00524	0	0.00262	0.00262
narcolepsy_C12	C7	Latorre et al. 2018	30330	0	0	0.00659	0.01319	0	0	0.00989	0.0033	0.01319	0.02967	0.07583	0.46489	3.32674	8.11078	17.075503	23.4289	23.485	13.6795	5.75008	2.65084	1.22321	0.40884	0.17804	0.04616	0.01319	0.00659	0	0	0
narcolepsy_C13	C8	Latorre et al. 2018	41663	0	0	0.0024	0	0.0024	0	0	0.012	0.0312	0.0216	0.0432	0.42724	3.34589	7.87989	17.807167	23.8173	23.066	13.0355	5.84931	2.76264	1.0969	0.50164	0.17522	0.09121	0.0216	0.0048	0.0024	0.0024	0
narcolepsy_C14	C9	Latorre et al. 2018	36260	0	0	0	0	0	0	0	0.01105	0.01381	0.058	0.08562	0.38391	2.70121	7.50704	17.712534	22.9409	23.9214	13.3541	6.32492	2.88626	1.2567	0.52754	0.1961	0.06905	0.02762	0.01105	0.00829	0.00276	0
narcolepsy_C15	C10	Latorre et al. 2018	19999	0	0	0	0	0	0.025	0.01	0.015	0.005	0.04	0.05	0.36002	3.34017	7.53038	18.175909	22.7211	23.2512	12.9256	6.26531	3.07015	1.33007	0.56503	0.18501	0.08	0.035	0.02	0	0	0
Don A _entero	C13	Cassotta et al. 2020	34819	0	0	0	0	0.00862	0.00574	0.00574	0.00574	0.00862	0.04021	0.08042	0.4509	3.07016	7.03352	15.290502	22.5423	24.59	14.6012	6.67739	3.2712	1.38143	0.59738	0.21253	0.09765	0.02298	0.00287	0.00287	0	0
Don B _entero	C14	Cassotta et al. 2020	36301	0	0	0	0.00275	0	0.00275	0	0.00826	0.01653	0.04683	0.06611	0.31955	2.86493	7.11275	16.740586	22.7597	23.9277	14.242	6.64445	3.06328	1.28371	0.53167	0.21762	0.07989	0.04959	0.00826	0.00551	0.00551	0
P34 COVID	C15	Low et al. 2021	36215	0	0	0.00112	0.00112	0	0.00561	0.00449	0.00898	0.01235	0.02918	0.07408	0.37378	3.0419	7.44649	17.081795	23.5416	24.0939	13.5168	6.19268	2.73996	1.11574	0.43889	0.17623	0.06959	0.01235	0.01571	0.00449	0.00112	0
infectious_DonA_EA	B1	Cassotta et al. 2020	839	0	0	0	0	0	0	0	0	0	0	0	0.11919	1.66865	2.38379	3.2181168	15.2563	66.7461	3.93325	2.50298	3.69487	0.23838	0.11919	0	0	0.11919	0	0	0	0
infectious_DonB_EA	B2	Cassotta et al. 2020	566	0	0	0	0	0	0	0	0	0	0	0	0.35336	4.41696	7.24382	19.964664	24.0283	18.1979	12.7208	8.65724	3.71025	0.53004	0.17668	0	0	0	0	0	0	0
infectious_DonC_EA	B3	Cassotta et al. 2020	169	0	0	0	0.59524	0.59524	0	0.59524	0.59524	0	0	0	0	0.59524	4.16667	18.452381	23.2143	27.9762	11.3095	8.33333	2.97619	0.59524	0.59524	0	0	0	0	0	0	0
infectious_DonA_EC	B4	Cassotta et al. 2020	65	0	0	0	0	0	0	0	0	0	0	0	0	6.15385	3.07692	3.0769231	18.4615	40	7.69231	9.23077	10.7692	0	1.53846	0	0	0	0	0	0	0
infectious_DonB_EC	B5	Cassotta et al. 2020	890	0	0	0	0	0	0	0	0	0	0	0	0.5618	5.39326	7.86517	23.033708	21.1236	18.8764	11.3483	5.73034	3.82022	1.46067	0.44944	0.22472	0.11236	0	0	0	0	0
P28_Tem_SARS-CoV-2_Nuc	V1	Low et al. 2021	827	0	0	0	0	0	0	0	0	0	0.12092	0	1.57195	5.19952	4.35308	18.258767	25.7557	21.7654	13.7848	5.0786	2.41838	1.45103	0.24184	0	0	0	0	0	0	0
P28_Tem_SARS-CoV-2_Spike	V2	Low et al. 2021	666	0	0	0	0	0	0	0	0	0	0.15015	0	0.15015	1.05105	3.45345	20.42042	21.021	23.8739	18.6186	3.3033	6.60661	0.9009	0.45045	0	0	0	0	0	0	0
SARS-CoV-2-specific	Virus-specific	current manuscript	92	0	0	0	0	0	0	0	0	0	0	8.69565	3.26087	19.5652	17.3913	25.00000	17.3913	7.6087	1.08696	0	0	0	0	0	0	0	0	0	0	0
PNS-myelin specific	PNS-myelin specific	current manuscript	166	0	0	0	0	0	0	0	0.60241	0.60241	4.81928	18.6747	15.6627	30.1205	10.8434	9.0361446	6.62651	1.20482	1.80723	0	0	0	0	0	0	0	0	0	0	0

Supplementary Table 4

HLA haplotypes of GBS patients. Human leukocyte antigen (HLA) typing of the GBS patients included in the study.

	DRB1	DRB3	DRB4	DRB5	DQB1	DQA1	DPB1	DPA1	HLA-A	HLA-B	HLA-C
PT1	11:04:01:01	02:02:01:02		01:01:01:01	06:02:01:01	01:02:01:10	02:01:02:01	01:03:01:04	01:01:01:01	07:02:01:01	04:01:01:06
	15:01:01:01				06:03:01:01	01:03:01:02	04:01:01:01	02:01:01:02	02:01:01:01	35:02:01:01	07:02:01:03
PT2	10:01:01:01	02:02:01:14			03:01:01:03	01:05:01:02	17:01:01:01	02:01:01:03	24:02:01:01	45:01:01:01	06:02:01:01
	11:01:02:01				05:01:01:02	05:11:01:01	105:01:01:01	03:01:01:05	68:01:01:02	58:02:01:01	16:01:01:01
PT3	03:01:01:01	01:01:02:01			02:01:01:01	04:01:01:01	04:01:01:01	01:03:01:02	02:01:01:01	08:01:01:01	07:01:01:01
	08:01:01:01				04:02:01:01	05:01:01:02	11:01:01:01	02:01:01:01	24:02:01:01	51:01:01:03	14:02:01:01
PT4	13:02:01:02	03:01:01:01		01:01:01:01	06:02:01:01	01:02:01:01	03:01:01:01	01:03:01:03	02:01:01:01	07:02:01:01	03:04:01:01
	15:01:01:01				06:04:01:01	01:02:01:04	04:01:01:01	01:03:01:04	03:01:01:01	40:01:02:01	07:02:01:20
PT5	11:01:01:01	02:02:01:02			03:01:01:03	01:02:01:04	02:01:02:01	01:03:01:01	01:01:01:01	35:03:01:01	04:01:01:01
	13:02:01:02	03:01:01:01			06:04:01:01	05:05:01:01	02:01:02:01	01:03:01:01	02:01:01:01	44:02:01:03	07:04:01:01
PT6	07:01:01:01	02:02:01:02	01:03:01:02N		03:01:01:03	02:01:01:01	03:01:01:01	01:03:01:03	01:01:01:01	18:01:01:02	06:02:01:01
	11:04:01:01				03:03:02:01	05:05:01:01	13:01:01:02	2:01:04	02:01:01:01	57:01:01:01	07:01:01:01
PT7	01:01:01:01		01:03:01:01		03:01:01:01	01:01:01:01	04:01:01:83	01:03:01:02	02:01:01:01	35:01:01:02	04:01:01:01
	04:01:01:01				05:01:01:03	03:03:01:01	104:01:01:03	01:03:01:12	32:01:01:01	44:02:01:01	05:01:01:02
PT8	04:01:01:01		01:03:01:01	2:02:01	03:01:01:01	01:02:02:01	04:01:01:01	01:03:01:04	02:01:01:01	40:02:01:01	02:02:02:01
	16:01:01:01				05:02:01:01	03:03:01:01	04:01:01:01	01:03:01:04	03:01:01:01	44:02:01:01	05:01:01:02
PT9	04:01:01:01		01:03:01:01		02:02:01:01	02:01:01:01	04:01:01:01	01:03:01:02	02:01:01:01	13:02:01:01	06:02:01:01
	07:01:01:01				03:02:01:01	03:01:01:01	04:01:01:01	01:03:01:02	24:02:01:01	51:01:01:01	15:02:01:01
PT10	03:01:01:01	01:01:02:01			02:01:01:01	01:04:01:01	04:02:01:02	01:03:01:05	01:01:01:01	08:01:01:01	07:01:01:01
	14:54:01:01	02:02:01:03			05:03:01:01	05:01:01:02	09:01:01:01	02:01:01:02	11:01:01:01	18:03:01:01	07:01:01:01
PT11	04:07:01:01		01:03:01:01	1:02:01	03:01:01:01	01:03:01:01	03:01:01:01	01:03:01:03	01:01:01:01	38:01:01:01	12:02:02:01
	15:02:01:02				06:01:01:01	03:03:01:01	04:01:01:41	02:07:01:01	03:01:01:01	52:01:01:02	12:03:01:01
PT12	07:01:01:01		01:01:01:01		02:02:01:01	02:01:01:01	02:01:02:01	01:03:01:01	23:01:01:01	44:03:01:01	03:03:01:01
	07:01:01:01				02:02:01:01	02:01:01:01	04:01:01:01	01:03:01:02	26:01:01:01	55:01:01:01	04:01:01:01
PT13	04:04:01:02	02:02:01:02	01:03:02:01		03:01:01:03	03:01:01:01	02:01:02:01	01:03:01:01	02:01:01:01	39:01:01:03	07:02:01:03
	11:01:01:01				03:02:01:02	05:05:01:01	04:01:01:01	01:03:01:04	03:01:01:01	51:01:01:01	12:03:01:01
PT14	01:01:01:01				04:02:01:04	01:01:01:01	03:01:01:01	01:03:01:02	01:01:01:01	15:01:01:06	01:02:01:01
	08:01:01:01				05:01:01:03	04:01:01:01	04:01:01:01	01:03:01:03	02:01:01:01	52:01:01:02	12:02:02:01
PT15	13:01:01:01	01:01:02:04			06:03:01:01	01:02:01:04	03:01:01:01	01:03:01:02	24:02:01:01	15:01:01:01	03:03:01:01
	13:02:01:02	03:01:01:01			06:04:01:01	01:03:01:02	04:01:01:01	01:03:01:03	33:03:01:01	39:01:01:03	12:03:01:01
PT16	03:01:01:01	01:01:02:01		01:01:01:01	02:01:01:01	01:02:01:10	04:02:01:02	01:03:01:05	01:01:01:01	08:01:01:01	04:01:01:01
	15:01:01:01				06:02:01:01	05:01:01:02	14:01:01:01	02:01:01:02	03:01:01:01	35:01:01:02	07:01:01:01

Supplementary Table 5

GLYPH2 Clusters. List of TCRβ specificity clusters obtained from GLYPH2 analysis and including PNS-myelin specific clonotypes based on global or local similarity.

Cluster ID	Cluster Motif ID	Seq ID	CDR3β	TRBV	TRBJ	PT/ C (ID)	Counts	Disease State	Source	Population	Clonotype ID*	ultCDR3β	Identity Type	Total Score	Fisher Score	V gene Score	Clonal Expansion Score	HLA score
CLUSTER_1	SL%YE_ADEFGINQRSTV	18	CASSLAYEQYF	TCRBV07-08	TCRBJ02-07	PT14	2	AC+ REC GBS	Blood	P2/PMP22-specific memory CD4+ T cell clone	CD4_136	CASSLaYEQYF	global	3.00E-11	0.036	0.001	0.38	0.041
CLUSTER_1	SL%YE_ADEFGINQRSTV	35623	CASSLVYEQFF	TRBV11-2	TRBJ2-1	PT1	7	AC GBS	Blood	Ex vivo total memory CD4+ T cells		CASSLvyEQFF	global	3.00E-11	0.036	0.001	0.38	0.041
CLUSTER_1	SL%YE_ADEFGINQRSTV	38685	CASSLAYEQYF	TRBV12-3/12-4	TRBJ2-7	PT1	4	AC GBS	Blood	Ex vivo total memory CD4+ T cells		CASSLaYEQYF	global	3.00E-11	0.036	0.001	0.38	0.041
CLUSTER_1	SL%YE_ADEFGINQRSTV	42201	CASSLSYEQYF	TRBV28-1	TRBJ2-7	PT1	2	AC GBS	Blood	Ex vivo total memory CD4+ T cells		CASSLSYEQYF	global	3.00E-11	0.036	0.001	0.38	0.041
CLUSTER_1	SL%YE_ADEFGINQRSTV	48998	CASSLGYEQYF	TRBV28-1	TRBJ2-7	PT1	2	AC GBS	Blood	Ex vivo total memory CD4+ T cells		CASSLgYEQYF	global	3.00E-11	0.036	0.001	0.38	0.041
CLUSTER_1	SL%YE_ADEFGINQRSTV	49272	CASSLQYEQYF	TRBV7-8	TRBJ2-7	PT1	2	AC GBS	Blood	Ex vivo total memory CD4+ T cells		CASSLqYEQYF	global	3.00E-11	0.036	0.001	0.38	0.041
CLUSTER_1	SL%YE_ADEFGINQRSTV	79213	CASSLGYEQYF	TCRBV07-02	TCRBJ02-07	PT2	1	AC GBS	Blood	Ex vivo total memory CD4+ T cells		CASSLgYEQYF	global	8.9E-12	0.075	0.001	0.41	0.2
CLUSTER_1	SL%YE_ADEFGINQRSTV	82013	CASSLGYEQYF	TCRBV12-03/12-04	TCRBJ02-07	PT2	1	AC GBS	Blood	Ex vivo total memory CD4+ T cells		CASSLgYEQYF	global	8.9E-12	0.075	0.001	0.41	0.2
CLUSTER_1	SL%YE_ADEFGINQRSTV	79219	CASSLSYEQYF	TCRBV12-03/12-04	TCRBJ02-07	PT2	2	AC GBS	Blood	Ex vivo total memory CD4+ T cells		CASSLSYEQYF	global	8.9E-12	0.075	0.001	0.41	0.2
CLUSTER_1	SL%YE_ADEFGINQRSTV	18843	CASSLSYEQFF	TRBV7-9	TRBJ2-1	PT5	11	AC GBS	Blood	Ex vivo total memory CD4+ T cells		CASSLsyEQFF	global	3.00E-11	0.036	0.001	0.38	0.041
CLUSTER_1	SL%YE_ADEFGINQRSTV	19524	CASSLNYEQYF	TRBV28-1	TRBJ2-7	PT5	10	AC GBS	Blood	Ex vivo total memory CD4+ T cells		CASSLnYEQYF	global	3.00E-11	0.036	0.001	0.38	0.041
CLUSTER_1	SL%YE_ADEFGINQRSTV	19534	CASSLGYEQYF	TRBV28-1	TRBJ2-7	PT5	7	AC GBS	Blood	Ex vivo total memory CD4+ T cells		CASSLgYEQYF	global	3.00E-11	0.036	0.001	0.38	0.041
CLUSTER_1	SL%YE_ADEFGINQRSTV	21046	CASSLAYEQYF	TRBV7-2	TRBJ2-7	PT5	6	AC GBS	Blood	Ex vivo total memory CD4+ T cells		CASSLaYEQYF	global	3.00E-11	0.036	0.001	0.38	0.041
CLUSTER_1	SL%YE_ADEFGINQRSTV	25611	CASSLTYEQYF	TRBV7-2	TRBJ2-7	PT5	3	AC GBS	Blood	Ex vivo total memory CD4+ T cells		CASSLtYEQYF	global	3.00E-11	0.036	0.001	0.38	0.041
CLUSTER_1	SL%YE_ADEFGINQRSTV	26117	CASSLQYEQYF	TRBV3-1/03-2	TRBJ2-7	PT5	2	AC GBS	Blood	Ex vivo total memory CD4+ T cells		CASSLqYEQYF	global	3.00E-11	0.036	0.001	0.38	0.041
CLUSTER_1	SL%YE_ADEFGINQRSTV	27951	CASSLFYEQYF	TRBV27-1	TRBJ2-7	PT5	2	AC GBS	Blood	Ex vivo total memory CD4+ T cells		CASSLfYEQYF	global	3.00E-11	0.036	0.001	0.38	0.041
CLUSTER_1	SL%YE_ADEFGINQRSTV	32129	CASSLAYEQYF	TRBV7-9	TRBJ2-7	PT5	2	AC GBS	Blood	Ex vivo total memory CD4+ T cells		CASSLaYEQYF	global	3.00E-11	0.036	0.001	0.38	0.041
CLUSTER_1	SL%YE_ADEFGINQRSTV	33692	CASSLIYEQYF	TRBV7-2	TRBJ2-7	PT5	2	AC GBS	Blood	Ex vivo total memory CD4+ T cells		CASSLiYEQYF	global	3.00E-11	0.036	0.001	0.38	0.041
CLUSTER_1	SL%YE_ADEFGINQRSTV	7941	CASSLGYEQYF	TRBV28-1	TRBJ2-7	PT7	3	AC GBS	Blood	Ex vivo total memory CD4+ T cells		CASSLgYEQYF	global	3.00E-11	0.036	0.001	0.38	0.041
CLUSTER_1	SL%YE_ADEFGINQRSTV	9432	CASSLSYEQYF	TRBV11-3	TRBJ2-7	PT7	5	AC GBS	Blood	Ex vivo total memory CD4+ T cells		CASSLSYEQYF	global	3.00E-11	0.036	0.001	0.38	0.041
CLUSTER_1	SL%YE_ADEFGINQRSTV	14040	CASSLGYEQYF	TRBV5-1	TRBJ2-7	PT7	2	AC GBS	Blood	Ex vivo total memory CD4+ T cells		CASSLgYEQYF	global	3.00E-11	0.036	0.001	0.38	0.041
CLUSTER_1	SL%YE_ADEFGINQRSTV	14045	CASSLSYEQYF	TRBV11-3	TRBJ2-7	PT7	2	AC GBS	Blood	Ex vivo total memory CD4+ T cells		CASSLSYEQYF	global	3.00E-11	0.036	0.001	0.38	0.041
CLUSTER_1	SL%YE_ADEFGINQRSTV	16026	CAWSLTYEQYF	TRBV30-1	TRBJ2-7	PT7	2	AC GBS	Blood	Ex vivo total memory CD4+ T cells		CAWSltYEQYF	global	3.00E-11	0.036	0.001	0.38	0.041
CLUSTER_1	SL%YE_ADEFGINQRSTV	17237	CASSLGYEQYF	TRBV5-6	TRBJ2-7	PT7	2	AC GBS	Blood	Ex vivo total memory CD4+ T cells		CASSLgYEQYF	global	3.00E-11	0.036	0.001	0.38	0.041
CLUSTER_1	SL%YE_ADEFGINQRSTV	17395	CASSLEYEQYF	TRBV7-9	TRBJ2-7	PT7	2	AC GBS	Blood	Ex vivo total memory CD4+ T cells		CASSLeYEQYF	global	3.00E-11	0.036	0.001	0.38	0.041
CLUSTER_1	SL%YE_ADEFGINQRSTV	18024	CASSLRYEQYF	TRBV12-3/12-4	TRBJ2-7	PT7	25	AC GBS	Blood	Ex vivo total memory CD4+ T cells		CASSLrYEQYF	global	3.00E-11	0.036	0.001	0.38	0.041
CLUSTER_1	SL%YE_ADEFGINQRSTV	54751	CASSLDYEQYF	TCRBV05-04	TCRBJ02-07	PT11	1	AC GBS	Blood	Ex vivo total memory CD4+ T cells		CASSLdYEQYF	global	3.00E-11	0.036	0.001	0.38	0.041
CLUSTER_1	SL%YE_ADEFGINQRSTV	57286	CASSLNYEQYF	TCRBV05-06	TCRBJ02-07	PT11	1	AC GBS	Blood	Ex vivo total memory CD4+ T cells		CASSLnYEQYF	global	3.00E-11	0.036	0.001	0.38	0.041
CLUSTER_1	SL%YE_ADEFGINQRSTV	60428	CASSLNYEQYF	TCRBV05-04	TCRBJ02-07	PT12	1	AC GBS	Blood	Ex vivo total memory CD4+ T cells		CASSLnYEQYF	global	3.00E-11	0.036	0.001	0.38	0.041
CLUSTER_1	SL%YE_ADEFGINQRSTV	64181	CASSLNYEQYF	TCRBV05-01	TCRBJ02-07	PT12	1	AC GBS	Blood	Ex vivo total memory CD4+ T cells		CASSLnYEQYF	global	3.00E-11	0.036	0.001	0.38	0.041
CLUSTER_1	SL%YE_ADEFGINQRSTV	67489	CASSLTYEQFF	TCRBV07	TCRBJ02-01	PT12	1	AC GBS	Blood	Ex vivo total memory CD4+ T cells		CASSLtyEQFF	global	3.00E-11	0.036	0.001	0.38	0.041
CLUSTER_1	SL%YE_ADEGKNRS	84751	CASSLAYEQYF	TCRBV28-01	TCRBJ02-07	PT2	1	REC GBS	Blood	Ex vivo total memory CD4+ T cells		CASSLaYEQYF	global	3.00E-11	0.6	0.001	0.76	0.16
CLUSTER_1	SL%YE_ADEGKNRS	88925	CASSLDYEQYF	TCRBV07-06	TCRBJ02-07	PT2	1	REC GBS	Blood	Ex vivo total memory CD4+ T cells		CASSLdYEQYF	global	3.00E-11	0.6	0.001	0.76	0.16
CLUSTER_1	SL%YE_ADEGKNRS	98227	CASSLEYEQYF	TCRBV11-02	TCRBJ02-07	PT2	1	REC GBS	Blood	Ex vivo total memory CD4+ T cells		CASSLeYEQYF	global	3.00E-11	0.6	0.001	0.76	0.16
CLUSTER_1	SL%YE_ADEGKNRS	105801	CASSLGYEQYF	TCRBV11-02	TCRBJ02-07	PT2	1	REC GBS	Blood	Ex vivo total memory CD4+ T cells		CASSLgYEQYF	global	3.00E-11	0.6	0.001	0.76	0.16
CLUSTER_1	SL%YE_ADEGKNRS	98223	CASSLNYEQYF	TCRBV07-02	TCRBJ02-07	PT2	1	REC GBS	Blood	Ex vivo total memory CD4+ T cells		CASSLnYEQYF	global	3.00E-11	0.6	0.001	0.76	0.16
CLUSTER_1	SL%YE_ADEGKNRS	106099	CASSLRYEQYF	TCRBV03-01/03-02	TCRBJ02-07	PT2	1	REC GBS	Blood	Ex vivo total memory CD4+ T cells		CASSLrYEQYF	global	3.00E-11	0.6	0.001	0.76	0.16
CLUSTER_1	SL%YE_ADEGKNRS	80032	CASSLSYEQYF	TCRBV12-03/12-04	TCRBJ02-07	PT2	5	REC GBS	Blood	Ex vivo total memory CD4+ T cells		CASSLSYEQYF	global	3.00E-11	0.6	0.001	0.76	0.16
CLUSTER_1	SL%YE_ADEGKNRS	81407	CASSLSYEQYF	TCRBV28-01	TCRBJ02-07	PT2	2	REC GBS	Blood	Ex vivo total memory CD4+ T cells		CASSLSYEQYF	global	3.00E-11	0.6	0.001	0.76	0.16
CLUSTER_1	SL%YE_ADEGKNRS	88478	CASSLSYEQYF	TCRBV12-03/12-04	TCRBJ02-07	PT2	1	REC GBS	Blood	Ex vivo total memory CD4+ T cells		CASSLSYEQYF	global	3.00E-11	0.6	0.001	0.76	0.16
CLUSTER_1	SL%YE_ADEGKNRS	90365	CASSLSYEQYF	TCRBV03-01/03-02	TCRBJ02-07	PT2	1	REC GBS	Blood	Ex vivo total memory CD4+ T cells		CASSLSYEQYF	global	3.00E-11	0.6	0.001	0.76	0.16
CLUSTER_1	SL%YE_ADEGKNRS	108026	CSASLSYEQYF	TCRBV20-01	TCRBJ02-07	PT2	1	REC GBS	Blood	Ex vivo total memory CD4+ T cells		CSASlSYEQYF	global	3.00E-11	0.6	0.001	0.76	0.16
CLUSTER_1	SL%YE_ADEFGINQRSTV	45012	CASSTAYEQYF	TCRBV12-03/12-04	TCRBJ02-07	PT5	6	REC GBS	Blood	Ex vivo total memory CD4+ T cells		CASStaYEQYF	global	3.00E-11	0.036	0.001	0.78	0.27
CLUSTER_1	S%AYE_AFGLTV	46426	CASSLAYEQYF	TCRBV07-02	TCRBJ02-07	PT5	3	REC GBS	Blood	Ex vivo total memory CD4+ T cells		CASSLaYEQYF	global	2.10E-10	8.40E-01	0.001	0.78	0.27
CLUSTER_1	S%AYE_AFGLTV	52131	CASSFAYEQYF	TCRBV11-02	TCRBJ02-07	PT5	1	REC GBS	Blood	Ex vivo total memory CD4+ T cells		CASSfaYEQYF	global	2.10E-10	8.40E-01	0.001	0.78	0.27
CLUSTER_1	S%AYE_AFGLTV	58948	CASSLAYEQYF	TCRBV28-01	TCRBJ02-07	PT5	2	REC GBS	Blood	Ex vivo total memory CD4+ T cells		CASSLaYEQYF	global	2.10E-10	8.40E-01	0.001	0.78	0.27
CLUSTER_1	S%AYE_AFGLTV	68180	CASSGAYEQYF	TCRBV06	TCRBJ02-07	PT5	2	REC GBS	Blood	Ex vivo total memory CD4+ T cells		CASSgaYEQYF	global	2.10E-10	8.40E-01	0.001	0.78	0.27
CLUSTER_1	S%AYE_AFGLTV	79752	CSASLAYEQYF	TCRBV20-01	TCRBJ02-07	PT5	1	REC GBS	Blood	Ex vivo total memory CD4+ T cells		CSASLAYEQYF	global	2.10E-10	8.40E-01	0.001	0.78	0.27
CLUSTER_1	S%AYE_AFGLTV	86225	CASSTAYEQYF	TCRBV11-02	TCRBJ02-07	PT5	1	REC GBS	Blood	Ex vivo total memory CD4+ T cells		CASStaYEQYF	global	2.10E-10	8.40E-01	0.001	0.78	0.27
CLUSTER_1	S%AYE_AFGLTV	90655	CASSAAYEQYF	TCRBV02-01	TCRBJ02-07	PT5	1	REC GBS	Blood	Ex vivo total memory CD4+ T cells		CASSaaYEQYF	global	2.10E-10	8.40E-01	0.001	0.78	0.27
CLUSTER_1	S%AYE_AFGLTV	92978	CSASAAYEQYF	TCRBV20-01	TCRBJ02-07	PT5	1	REC GBS	Blood	Ex vivo total memory CD4+ T cells		CSASaaYEQYF	global	2.10E-10	8.40E-01	0.001	0.78	0.27
CLUSTER_1	S%AYE_AFGLTV	93885	CASSVAYEQFF	TCRBV09-01	TCRBJ02-01	PT5	1	REC GBS	Blood	Ex vivo total memory CD4+ T cells		CASSVayEQFF	global	2.10E-10	8.40E-01	0.001	0.78	0.27
CLUSTER_1	S%AYE_AFGLTV	108516	CASSLAYEQYF	TCRBV07-06	TCRBJ02-07	PT5	1	REC GBS	Blood	Ex vivo total memory CD4+ T cells		CASSLaYEQYF	global	2.10E-10	8.40E-01	0.001	0.78	0.27
CLUSTER_1	S%AYE_AFGLTV	114403	CASSVAYEQYF	TCRBV09-01	TCRBJ02-07	PT5	1	REC GBS	Blood	Ex vivo total memory CD4+ T cells		CASSVaYEQYF	global	2.10E-10	8.40E-01	0.001	0.78	0.27
CLUSTER_1	S%AYE_AFGLTV	124470	CASSLAYEQYF	TCRBV07-09	TCRBJ02-07	PT7	2	REC GBS	Blood	Ex vivo total memory CD4+ T cells		CASSLaYEQYF	global	2.10E-10	8.40E-01	0.001	0.78	0.27
CLUSTER_1	S%AYE_AFGLTV	128076	CASSGAYEQYF	TCRBV07-09	TCRBJ02-07	PT7	2	REC GBS	Blood	Ex vivo total memory CD4+ T cells		CASSgaYEQY	global	2.10E-10	8.40E-01	0.001	0.78	0.27
CLUSTER_1	S%AYE_AFGLTV	21112	CASSLGYEQYF	TCRBV28-01	TCRBJ02-07	PT9	1	REC GBS	Blood	Ex vivo total memory CD4+ T cells		CASSLgYEQYF	global	2.10E-10	8.40E-01	0.001	0.78	0.27
CLUSTER_1	S%AYE_AFGLTV	21567	CASSLRYEQYF	TCRBV28-01	TCRBJ02-07	PT9	13	REC GBS	Blood	Ex vivo total memory CD4+ T cells		CASSLrYEQYF	global	2.10E-10	8.40E-01	0.001	0.78	0.27
CLUSTER_1	S%AYE_AFGLTV	21755	CASSLTYEQYF	TCRBV07-02	TCRBJ02-07	PT9	1	REC GBS	Blood	Ex vivo total memory CD4+ T cells		CASSLtYEQYF	global	2.10E-10	8.40E-01	0.001	0.78	0.27
CLUSTER_1	S%AYE_AFGLTV	22052	CASSLTYEQYF	TCRBV07-09	TCRBJ02-07	PT9	12	REC GBS	Blood	Ex vivo total memory CD4+ T cells		CASSLtYEQYF	global	2.10E-10	8.40E-01	0.001	0.78	0.27
CLUSTER_1	S%AYE_AFGLTV	23090	CASSLDYEQYF	TCRBV05-01	TCRBJ02-07	PT9	2	REC GBS	Blood	Ex vivo total memory CD4+ T cells		CASSLdYEQYF	global	2.10E-10	8.40E-01	0.001	0.78	0.27
CLUSTER_1	S%AYE_AFGLTV	25060	CASSLSYEQYF	TCRBV18-01	TCRBJ02-07	PT9	1	REC GBS	Blood	Ex vivo total memory CD4+ T cells		CASSLSYEQYF	global	2.10E-10	8.40E-01	0.001	0.78	0.27
CLUSTER_1	S%AYE_AFGLTV	29591	CASSLCYEQYF	TCRBV06-07	TCRBJ02-07	PT9	2	REC GBS	Blood	Ex vivo total memory CD4+ T cells		CASSLcYEQYF	global	2.10E-10	8.40E-01	0.001	0.78	0.27
CLUSTER_1	S%AYE_AFGLTV	34706	CASSLDYEQYF	TCRBV07-09	TCRBJ02-07	PT9	1	REC GBS	Blood	Ex vivo total memory CD4+ T cells		CASSLdYEQYF	global	2.10E-10	8.40E-01	0.001	0.78	0.27
CLUSTER_1	S%AYE_AFGLTV	36428	CASSLAYEQYF	TCRBV05-01	TCRBJ02-07	PT9	1	REC GBS	Blood	Ex vivo total memory CD4+ T cells		CASSLaYEQYF	global	2.10E-10	8.40E-01	0.001	0.78	0.27
CLUSTER_1	S%AYE_AFGLTV	44978	CASSLTYEQYF	TCRBV14-01	TCRBJ02-07	PT9	1	REC GBS	Blood	Ex vivo total memory CD4+ T cells		CASSLtYEQY	global	2.10E-10	8.40E-01	0.001	0.78	0.27
CLUSTER_1	S%AYE_AFGLTV	794	CASSLNYEQYF	TCRBV05-04	TCRBJ02-07	PT13	2	REC GBS	Blood	Ex vivo total memory CD4+ T cells		CASSLnYEQYF	global	2.10E-10	8.40E-01	0.001	0.78	0.27
CLUSTER_1	S%AYE_AFGLTV	819	CASSLSYEQYF	TCRBV07-02	TCRBJ02-07	PT13	1	REC GBS	Blood	Ex vivo total memory CD4+ T cells		CASSLSYEQYF	global	2.10E-10	8.40E-01	0.001	0.78	0.27
CLUSTER_1	S%AYE_AFGLTV	1276	CASSLAYEQFF	TCRBV05-05	TCRBJ02-01	PT13	2	REC GBS	Blood	Ex vivo total memory CD4+ T cells		CASSLayEQFF	global	2.10E-10	8.40E-01	0.001	0.78	0.27
CLUSTER_1	S%AYE_AFGLTV	2854	CASSLGYEQYF	TCRBV07	TCRBJ02-07	PT13	1	REC GBS	Blood	Ex vivo total memory CD4+ T cells		CASSLgYEQYF	global	2.10E-10	8.40E-01	0.001	0.78	0.27
CLUSTER_1	S%AYE_AFGLTV	7921	CASSLSYEQYF	TCRBV07	TCRBJ02-07	PT13	1	REC GBS	Blood	Ex vivo total memory CD4+ T cells		CASSLSYEQYF	global	2.10E-10	8.40E-01	0.001	0.78	0.27
CLUSTER_1	S%AYE_AFGLTV	15017	CASSLSYEQYF	TCRBV06-02/06-03	TCRBJ02-07	PT13	1	REC GBS	Blood	Ex vivo total memory CD4+ T cells		CASSLSYEQYF	global	2.10E-10	8.40E-01	0.001	0.78	0.27
CLUSTER_1	S%AYE_AFGLTV	15966	CATSLSYEQYF	TCRBV06-05	TCRBJ02-07	PT13	1	REC GBS	Blood	Ex vivo total memory CD4+ T cells		CATSlSYEQYF	global	2.10E-10	8.40E-01	0.001	0.78	0.27
CLUSTER_1	S%AYE_AFGLTV	17881	CASSLGYEQYF	TCRBV06-02	TCRBJ02-07	PT13	1	REC GBS	Blood	Ex vivo total memory CD4+ T cells		CASSLgYEQYF	global	2.10E-10	8.40E-01	0.001	0.78	0.27
CLUSTER_1	SL%YE_ADEGHLNRSTV	10318	CASSSAYEQYF	TCRBV06-05	TCRBJ02-07	C1	2	HD	Blood	Ex vivo total memory CD4+ T cells		CASSSaYEQYF	global	5.60E-11	0.001	0.001	0.73	0.2
CLUSTER_1	SL%YE_ADEGHLNRSTV	10435	CASSVAYEQYF	TCRBV07-02	TCRBJ02-07	C1	83	HD	Blood	Ex vivo total memory CD4+ T cells		CASSVaYEQYF	global	5.60E-11	0.001	0.001	0.73	0.2
CLUSTER_1	SL%YE_ADEGHLNRSTV	10873	CASSSAYEQYF	TCRBV06-06	TCRBJ02-07	C1	135	HD	Blood	Ex vivo total memory CD4+ T cells		CASSSaYEQYF	global	5.60E-11	0.001	0.001	0.73	0.2
CLUSTER_1	SL%YE_ADEGHLNRSTV	13451	CATSVAYEQYF	TCRBV06-05	TCRBJ02-07	C1	3	HD	Blood	Ex vivo total memory CD4+ T cells		CATSvaYEQYF	global	5.60E-11	0.001	0.001	0.73	0.2
CLUSTER_1	SL%YE_ADEGHLNRSTV	15045	CASSLAYEQYF	TCRBV11-02	TCRBJ02-07	C1	63	HD	Blood	Ex vivo total memory CD4+ T cells		CASSLaYEQYF	global	5.60E-11	0.001	0.001	0.73	0.2
CLUSTER_1	SL%YE_ADEGHLNRSTV	17923	CATSVAYEQYF	TCRBV06-01	TCRBJ02-07	C1	95	HD	Blood	Ex vivo total memory CD4+ T cells		CATSvaYEQYF	global	5.60E-11	0.001	0.001	0.73	0.2
CLUSTER_1	SL%YE_ADEGHLNRSTV	18127	CASSGAYEQYF	TCRBV02-01	TCRBJ02-07	C1	68	HD	Blood	Ex vivo total memory CD4+ T cells		CASSgaYEQYF	global	5.60E-11	0.001	0.001	0.73	0.2
CLUSTER_1	SL%YE_ADEGHLNRSTV	1951	CASSLAGEQFF	TCRBV05-08	TCRBJ02-01	C1	93	HD	Blood	Ex vivo total memory CD4+ T cells		CASSLAGEQFF	global	5.60E-11	0.001	0.001	0.73	0.2
CLUSTER_1	SL%YE_ADEGHLNRSTV	15045	CASSLAYEQYF	TCRBV11-02	TCRBJ02-07	C1	63	HD	Blood	Ex vivo total memory CD4+ T cells		CASSLaYEQYF	global	5.60E-11	0.001	0.001	0.73	0.2
CLUSTER_1	SL%YE_ADEGHLNRSTV	24742	CASSLAGEQFF	TCRBV05-01	TCRBJ02-01	C1	25	HD	Blood	Ex vivo total memory CD4+ T cells		CASSLAGEQFF	global	5.60E-11	0.001	0.001	0.73	0.2
CLUSTER_1	SL%YE_ADEGHLNRSTV	31053	CSASGAYEQYF	TCRBV20-01	TCRBJ02-07	C2	8	HD	Blood	Ex vivo total memory CD4+ T cells		CSASgaYEQYF	global	5.60E-11	0.001	0.001	0.73	0.2
CLUSTER_1	SL%YE_ADEGHLNRSTV	33045	CASSEAYEQYF	TCRBV06-05	TCRBJ02-07	C2	2	HD	Blood	Ex vivo total memory CD4+ T cells		CASSEaYEQYF	global	5.60E-11	0.001	0.001	0.73	0.2
CLUSTER_1	SL%YE_ADEGHLNRSTV	47604	CASSSAYEQYF	TCRBV07-09	TCRBJ02-07	C3	9	HD	Blood	Ex vivo total memory CD4+ T cells		CASSSaYEQYF	global	5.60E-11	0.001	0.001	0.73	0.2
CLUSTER_1	SL%YE_ADEGHLNRSTV	39063	CASSLAGEQYF	TCRBV05-06	TCRBJ02-05	C3	3	HD	Blood	Ex vivo total memory CD4+ T cells		CASSLAGEQYF	global	5.60E-11	0.001	0.001	0.73	0.2
CLUSTER_1	SL%YE_ADEGHLNRSTV	40691	CASSLANEQYF	TCRBV11-02	TCRBJ02-07	C3	18	HD	Blood	Ex vivo total memory CD4+ T cells		CASSLanEQYF	global	5.60E-11	0.001	0.001	0.73	0.2
CLUSTER_1	SL%YE_ADEGHLNRSTV	42948	CASSLAGEQYF	TCRBV05-06	TCRBJ02-07	C3	42	HD	Blood	Ex vivo total memory CD4+ T cells		CASSLAGEQYF	global	5.60E-11	0.001	0.001	0.73	0.2
CLUSTER_1	SL%YE_ADEGHLNRSTV	48309	CASSLADEQYF	TCRBV05-01	TCRBJ02-07	C3	86	HD	Blood	Ex vivo total memory CD4+ T cells		CASSLadEQYF	global	5.60E-11	0.001	0.001	0.73	0.2
CLUSTER_1	SL%YE_ADEGHLNRSTV	54108	CASSLAHEQFF	TCRBV07-03	TCRBJ02-01	C3	20	HD	Blood	Ex vivo total memory CD4+ T cells		CASSLahEQFF	global	5.60E-11	0.001	0.001	0.73	0.2
CLUSTER_1	SL%YE_ADEGHLNRSTV	59530	CASSLAGELFF	TCRBV28-01	TCRBJ02-02	C3	80	HD	Blood	Ex vivo total memory CD4+ T cells		CASSLaGELFF	global	5.60E-11	0.001	0.001	0.73	0.2
CLUSTER_1	SL%YE_ADEGHLNRSTV	61176	CASSQAYEQYF	TRBV3-1	TRBJ2-7	C4	3	HD	Blood	Ex vivo total memory CD4+ T cells		CASSQaYEQYF	global	5.60E-11	0.001	0.001	0.73	0.2
CLUSTER_1	SL%YE_ADEGLNQRSTV	229	CASSLGYEQYF	TCRBV13-01	TCRBJ02-07	C10	43	HD	Blood	Ex vivo total memory CD4+ T cells		CASSLgYEQYF	global	3.40E-11	0.001	0.001	0.22	0.2
CLUSTER_1	SL%YE_ADEGLNQRSTV	1340	CASSLSYEQYF	TCRBV05-08	TCRBJ02-07	C10	155	HD	Blood	Ex vivo total memory CD4+ T cells		CASSLSYEQYF	global	3.40E-11	0.001	0.001	0.22	0.2
CLUSTER_1	SL%YE_ADEGLNQRSTV	3488	CASSLEYEQFF	TCRBV07-09	TCRBJ02-01	C10	29	HD	Blood	Ex vivo total memory CD4+ T cells		CASSLeyEQFF	global	3.40E-11	0.001	0.001	0.22	0.2
CLUSTER_1	SL%YE_ADEGLNQRSTV	4933	CASSLSYEQYF	TCRBV05-06	TCRBJ02-07	C10	2	HD	Blood	Ex vivo total memory CD4+ T cells		CASSLSYEQYF	global	3.40E-11	0.001	0.001	0.22	0.2
CLUSTER_1	SL%YE_ADEGLNQRSTV	6518	CASSLSYEQYF	TCRBV07-09	TCRBJ02-07	C10	2	HD	Blood	Ex vivo total memory CD4+ T cells		CASSLSYEQYF	global	3.40E-11	0.001	0.001	0.22	0.2
CLUSTER_1	SL%YE_ADEGLNQRSTV	11968	CASSLLYEQYF	TCRBV06-01	TCRBJ02-07	C10	2	HD	Blood	Ex vivo total memory CD4+ T cells		CASSLlYEQYF	global	3.40E-11	0.001	0.001	0.22	0.2
CLUSTER_1	SL%YE_ADEGLNQRSTV	20133	CASSLQYEQYF	TCRBV06-01	TCRBJ02-07	C11	84	HD	Blood	Ex vivo total memory CD4+ T cells		CASSLqYEQYF	global	3.40E-11	0.001	0.001	0.22	0.2
CLUSTER_1	SL%YE_ADEGLNQRSTV	20240	CASSLSYEQYF	TCRBV06-01	TCRBJ02-07	C11	74	HD	Blood	Ex vivo total memory CD4+ T cells		CASSLSYEQYF	global	3.40E-11	0.001	0.001	0.22	0.2
CLUSTER_1	SL%YE_ADEGLNQRSTV	22604	CASSLGYEQYF	TCRBV05-01	TCRBJ02-05	C11	2	HD	Blood	Ex vivo total memory CD4+ T cells		CASSLgYEQYF	global	3.40E-11	0.001	0.001	0.22	0.2
CLUSTER_1	SL%YE_ADEGLNQRSTV	23322	CASSLTYEQYF	TCRBV07-02	TCRBJ02-07	C11	45	HD	Blood	Ex vivo total memory CD4+ T cells		CASSLtYEQYF	global	3.40E-11	0.001	0.001	0.22	0.2
CLUSTER_1	SL%YE_ADEGLNQRSTV	23361	CASSLRYEQYF	TCRBV02-01	TCRBJ02-07	C11	70	HD	Blood	Ex vivo total memory CD4+ T cells		CASSLrYEQYF	global	3.40E-11	0.001	0.001	0.22	0.2
CLUSTER_1	SL%YE_ADEGLNQRSTV	25630	CASSLEYEQFF	TCRBV05-04	TCRBJ02-01	C11	57	HD	Blood	Ex vivo total memory CD4+ T cells		CASSLeyEQFF	global	3.40E-11	0.001	0.001	0.22	0.2
CLUSTER_1	SL%YE_ADEGLNQRSTV	30519	CASSLDYEQYF	TCRBV11-03	TCRBJ02-07	C11	44	HD	Blood	Ex vivo total memory CD4+ T cells		CASSLdYEQYF	global	3.40E-11	0.001	0.001	0.22	0.2
CLUSTER_1	SL%YE_ADEGLNQRSTV	30783	CASSLGYEQYF	TCRBV11-03	TCRBJ02-07	C11	42	HD	Blood	Ex vivo total memory CD4+ T cells		CASSLgYEQYF	global	3.40E-11	0.001	0.001	0.22	0.2
CLUSTER_1	SL%YE_ADEGLNQRSTV	34984	CASSLTYEQYF	TCRBV28-01	TCRBJ02-07	C12	16	HD	Blood	Ex vivo total memory CD4+ T cells		CASSLtYEQYF	global	3.40E-11	0.001	0.001	0.22	0.2
CLUSTER_1	SL%YE_ADEGLNQRSTV	35382	CASSLQYEQYF	TCRBV07-02	TCRBJ02-07	C12	16	HD	Blood	Ex vivo total memory CD4+ T cells		CASSLqYEQYF	global	3.40E-11	0.001	0.001	0.22	0.2
CLUSTER_1	SL%YE_ADEGLNQRSTV	39041	CASSLRYEQYF	TCRBV28-01	TCRBJ02-07	C12	27	HD	Blood	Ex vivo total memory CD4+ T cells		CASSLrYEQYF	global	3.40E-11	0.001	0.001	0.22	0.2
CLUSTER_1	SL%YE_ADEGLNQRSTV	39267	CASSLAYEQYF	TCRBV06-06	TCRBJ02-07	C12	24	HD	Blood	Ex vivo total memory CD4+ T cells		CASSLaYEQYF	global	3.40E-11	0.001	0.001	0.22	0.2
CLUSTER_1	SL%YE_ADEGLNQRSTV	39712	CASSLGYEQYF	TCRBV05-05	TCRBJ02-07	C12	6	HD	Blood	Ex vivo total memory CD4+ T cells		CASSLgYEQYF	global	3.40E-11	0.001	0.001	0.22	0.2
CLUSTER_1	SL%YE_ADEGLNQRSTV	42630	CASSLGYEQYF	TCRBV11-02	TCRBJ02-07	C12	8	HD	Blood	Ex vivo total memory CD4+ T cells		CASSLgYEQYF	global	3.40E-11	0.001	0.001	0.22	0.2
CLUSTER_1	SL%YE_ADEGLNQRSTV	43880	CASSLNYEQYF	TCRBV05-06	TCRBJ02-07	C12	100	HD	Blood	Ex vivo total memory CD4+ T cells		CASSLnYEQYF	global	3.40E-11	0.001	0.001	0.22	0.2
CLUSTER_1	SL%YE_ADEGLNQRSTV	45572	CASSLVYEQFF	TCRBV11-02	TCRBJ02-01	C12	10	HD	Blood	Ex vivo total memory CD4+ T cells		CASSLvyEQFF	global	3.40E-11	0.001	0.001	0.22	0.2
CLUSTER_1	SL%YE_ADEGLNQRSTV	46048	CASSLSYEQYF	TCRBV11-02	TCRBJ02-07	C12	20	HD	Blood	Ex vivo total memory CD4+ T cells		CASSLSYEQYF	global	3.40E-11	0.001	0.001	0.22	0.2

CLUSTER_1	SL%YE_ADEGLNQRSTV	60491	CASSLSYEQYF	TCRBV06-01	TCRBJ02-07	C12	30	HD	Blood	Ex vivo total memory CD4+ T cells		CASSLSYEQYF	global	3.40E-11	0.001	0.001	0.22	0.2
CLUSTER_1	SL%YE_ADEGLNQRSTV	64913	CASSLGYEQYF	TCRBV05-04	TCRBJ02-07	C12	25	HD	Blood	Ex vivo total memory CD4+ T cells		CASSLgYEQYF	global	3.40E-11	0.001	0.001	0.22	0.2
CLUSTER_1	SL%YE_ADEGLNQRSTV	65803	CASSLNYEQYF	TCRBV05-04	TCRBJ02-07	C12	26	HD	Blood	Ex vivo total memory CD4+ T cells		CASSLnYEQYF	global	3.40E-11	0.001	0.001	0.22	0.2
CLUSTER_1	SL%YE_ADEGLNQRSTV	67033	CASSLEYEQFF	TCRBV05-01	TCRBJ02-01	C12	25	HD	Blood	Ex vivo total memory CD4+ T cells		CASSLeyEQFF	global	3.40E-11	0.001	0.001	0.22	0.2
CLUSTER_1	SL%YE_ADEGLNQRSTV	68673	CAWSLGYEQYF	TCRBV30-01	TCRBJ02-07	C12	11	HD	Blood	Ex vivo total memory CD4+ T cells		CAWSlgYEQYF	global	3.40E-11	0.001	0.001	0.22	0.2
CLUSTER_1	SL%YE_ADEGHLNRSTV	68359	CASSKAYEQYF	TRBV5-1	TRBJ2-7	C13	2	HD	Blood	Ex vivo total memory CD4+ T cells		CASSkaYEQYF	global	5.60E-11	0.001	0.001	0.73	0.2
CLUSTER_1	SL%YE_ADEGHLNRSTV	71496	CASSLANEQFF	TRBV12-1	TRBJ2-1	C13	2	HD	Blood	Ex vivo total memory CD4+ T cells		CASSLaNEQFF	global	5.60E-11	0.001	0.001	0.73	0.2
CLUSTER_1	SL%YE_ADEGHLNRSTV	72783	CASSLAGEQYF	TRBV3-1	TRBJ2-7	C13	2	HD	Blood	Ex vivo total memory CD4+ T cells		CASSLAGEQYF	global	5.60E-11	0.001	0.001	0.73	0.2
CLUSTER_1	SL%YE_ADEGHLNRSTV	79664	CAWSTAYEQYF	TRBV30-1	TRBJ2-7	C15	2	HD	Blood	Ex vivo total memory CD4+ T cells		CAWStaYEQYF	global	5.60E-11	0.001	0.001	0.73	0.2
CLUSTER_2	SLE%GE_AGLTV	21	CASSLEVGEQFF	TCRBV07-02	TCRBJ02-01	PT6	58	AC GBS	Blood	P2-specific memory CD4+ T cell clone	CD4_107	CASSLevgEQFF	global	2.60E-10	0.45	0.015	0.03	0.041
CLUSTER_2	SLE%GE_AGLTV	33998	CASSLETGELFF	TRBV5-1	TRBJ2-2	PT1	38	AC GBS	Blood	Ex vivo total memory CD4+ T cells		CASSLeTGELFF	global	2.60E-10	0.45	0.015	0.03	0.041
CLUSTER_2	SLE%GE_AGLTV	37814	CASSLEAGEQYF	TRBV5-5	TRBJ2-7	PT1	4	AC GBS	Blood	Ex vivo total memory CD4+ T cells		CASSLeAGEQYF	global	2.60E-10	0.45	0.015	0.03	0.041
CLUSTER_2	SLE%GE_AGLTV	48555	CASSLDVGELFF	TRBV11-2	TRBJ2-2	PT1	2	AC GBS	Blood	Ex vivo total memory CD4+ T cells		CASSLdvGELFF	global	2.60E-10	0.45	0.015	0.03	0.041
CLUSTER_2	SLE%GE_AGLTV	74557	CASSLEAGEQYF	TCRBV11-02	TCRBJ02-07	PT2	1	AC GBS	Blood	Ex vivo total memory CD4+ T cells		CASSLeAGEQYF	global	3.50E-13	5.60E-01	0.002	0.041	0.041
CLUSTER_2	SLE%GE_AGLTV	19325	CASSLEVGELFF	TRBV11-3	TRBJ2-2	PT5	12	AC GBS	Blood	Ex vivo total memory CD4+ T cells		CASSLevGELFF	global	2.60E-10	0.45	0.015	0.03	0.041
CLUSTER_2	SLE%GE_AGLTV	21036	CASSLELGELFF	TRBV7-8	TRBJ2-2	PT5	6	AC GBS	Blood	Ex vivo total memory CD4+ T cells		CASSLelGELFF	global	2.60E-10	0.45	0.015	0.03	0.041
CLUSTER_2	SLE%GE_AGLTV	25973	CASSLELGEQFF	TRBV7-2	TRBJ2-1	PT5	3	AC GBS	Blood	Ex vivo total memory CD4+ T cells		CASSLelgEQFF	global	2.60E-10	0.45	0.015	0.03	0.041
CLUSTER_2	SLE%GE_AGLTV	26337	CASSLELGEQFF	TRBV7-2	TRBJ2-1	PT5	2	AC GBS	Blood	Ex vivo total memory CD4+ T cells		CASSLelgEQFF	global	2.60E-10	0.45	0.015	0.03	0.041
CLUSTER_2	SLE%GE_AGLTV	29602	CASSLELGEQFF	TRBV7-1	TRBJ2-1	PT5	2	AC GBS	Blood	Ex vivo total memory CD4+ T cells		CASSLelgEQFF	global	2.60E-10	0.45	0.015	0.03	0.041
CLUSTER_2	SLE%GE_AGLTV	5732	CASSLRVGEQYF	TRBV28-1	TRBJ2-7	PT7	4	AC GBS	Blood	Ex vivo total memory CD4+ T cells		CASSLrvgEQYF	global	2.60E-10	0.45	0.015	0.03	0.041
CLUSTER_2	SLE%GE_AGLTV	66086	CASSLEGGELFF	TCRBV27-01	TCRBJ02-02	PT12	1	AC GBS	Blood	Ex vivo total memory CD4+ T cells		CASSLegGELFF	global	2.60E-10	0.45	0.015	0.03	0.041
CLUSTER_2	SLE%GE_AGLTV	97018	CASSLEGGEAFF	TCRBV05-01	TCRBJ01-01	PT2	1	REC GBS	Blood	Ex vivo total memory CD4+ T cells		CASSLeGGEAFF	global	2.50E-11	0.7	0.006	0.45	0.011
CLUSTER_2	SLE%GE_AGLTV	105955	CASSLEGGEQYF	TCRBV06-05	TCRBJ02-07	PT2	1	REC GBS	Blood	Ex vivo total memory CD4+ T cells		CASSLeGGEQYF	global	2.50E-11	0.7	0.006	0.45	0.011
CLUSTER_2	SLE%GE_AGLTV	106904	CASSLEGGEQYF	TCRBV07-09	TCRBJ02-07	PT2	1	REC GBS	Blood	Ex vivo total memory CD4+ T cells		CASSLeGGEQYF	global	2.50E-11	0.7	0.006	0.45	0.011
CLUSTER_2	SLE%GE_AGLTV	80230	CASSLELGELFF	TCRBV05-01	TCRBJ02-02	PT2	5	REC GBS	Blood	Ex vivo total memory CD4+ T cells		CASSLelGELFF	global	2.50E-11	0.7	0.006	0.45	0.011
CLUSTER_2	SLE%GE_AGLTV	81491	CASSLETGEQYF	TCRBV05-04	TCRBJ02-07	PT2	2	REC GBS	Blood	Ex vivo total memory CD4+ T cells		CASSLeTGEQYF	global	2.50E-11	0.7	0.006	0.45	0.011
CLUSTER_2	SLE%GE_LTV	22097	CASSLETGELFF	TCRBV07-09	TCRBJ02-02	PT4	11	REC GBS	Blood	Ex vivo total memory CD4+ T cells		CASSLeTGELFF	global	6.20E-11	9.30E-01	0.006	0.012	0.2
CLUSTER_2	SLE%GE_LTV	43258	CASSLEVGELFF	TCRBV11-03	TCRBJ02-02	PT5	16	REC GBS	Blood	Ex vivo total memory CD4+ T cells		CASSLevGELFF	global	6.20E-11	9.30E-01	0.006	0.012	0.2
CLUSTER_2	SLE%GE_LTV	44846	CASSLELGELFF	TCRBV07-08	TCRBJ02-02	PT5	8	REC GBS	Blood	Ex vivo total memory CD4+ T cells		CASSLelGELFF	global	6.20E-11	9.30E-01	0.006	0.012	0.2
CLUSTER_2	SLE%GE_LTV	47485	CASSLELGEQFF	TCRBV07	TCRBJ02-01	PT5	6	REC GBS	Blood	Ex vivo total memory CD4+ T cells		CASSLelgEQFF	global	6.20E-11	9.30E-01	0.006	0.012	0.2
CLUSTER_2	SLE%GE_LTV	53938	CASSLEVGEQYF	TCRBV05-01	TCRBJ02-07	PT5	4	REC GBS	Blood	Ex vivo total memory CD4+ T cells		CASSLevgEQYF	global	6.20E-11	9.30E-01	0.006	0.012	0.2
CLUSTER_2	SLE%GE_LTV	98185	CASSLETGELFF	TCRBV07-02	TCRBJ02-02	PT5	1	REC GBS	Blood	Ex vivo total memory CD4+ T cells		CASSLeTGELFF	global	6.20E-11	9.30E-01	0.006	0.012	0.2
CLUSTER_2	SLE%GE_LTV	99174	CASSLELGEQFF	TCRBV11-02	TCRBJ02-01	PT5	1	REC GBS	Blood	Ex vivo total memory CD4+ T cells		CASSLelgEQFF	global	6.20E-11	9.30E-01	0.006	0.012	0.2
CLUSTER_2	SLE%GE_LTV	108744	CASSLEVGEQFF	TCRBV07-02	TCRBJ02-01	PT5	1	REC GBS	Blood	Ex vivo total memory CD4+ T cells		CASSLevgEQFF	global	6.20E-11	9.30E-01	0.006	0.012	0.2
CLUSTER_2	SLE%GE_AGLTV	46738	CASSLEAGELFF	TCRBV12-03/12-04	TCRBJ02-02	PT13	1	REC GBS	Blood	Ex vivo total memory CD4+ T cells		CASSLeaGELFF	global	2.50E-11	0.7	0.006	0.45	0.011
CLUSTER_3	S%GLAGAYE_KQRS	38	CASSSGLAGAYEQYF	TCRBV06-02/06-03	TCRBJ02-07	PT7	3	REC GBS	Blood	P2-specific memory CD4+ T cell clone	CD4_116	CASSSGLAGaYEQYF	global	4.80E-07	6.20E-01	0.34	0.81	0.033
CLUSTER_3	S%GLAGAYE_KQRS	43897	CASSKGLAGAYEQYF	TRBV5-1	TRBJ2-7	PT1	2	AC GBS	Blood	Ex vivo total memory CD4+ T cells		CASSkGLAGaYEQYF	global	4.80E-07	6.20E-01	0.34	0.81	0.033
CLUSTER_3	S%GLAGAYE_KQRS	47380	CASSRGLAGAYEQYF	TRBV27-1	TRBJ2-7	PT1	2	AC GBS	Blood	Ex vivo total memory CD4+ T cells		CASSRGLAGaYEQYF	global	4.80E-07	6.20E-01	0.34	0.81	0.033
CLUSTER_3	S%GLAGAYE_KQRS	24297	CASSQGLAGAYEQYF	TRBV4-2	TRBJ2-7	PT5	3	AC GBS	Blood	Ex vivo total memory CD4+ T cells		CASSQGLAGaYEQYF	global	4.80E-07	6.20E-01	0.34	0.81	0.033
CLUSTER_4	S%GQGTDT_DKQR	33	CASSQGQGTDTQYF	TCRBV04-01	TCRBJ02-03	PT7	1	REC GBS	Blood	P2-specific memory CD4+ T cell clone	CD4_115	CASSQGQGTDTQYF	global	4.90E-07	0.83	0.21	1	0.45
CLUSTER_4	S%GQGTDT_DKQR	30580	CATSDGQGTDTQYF	TRBV24-1	TRBJ2-3	PT5	2	AC GBS	Blood	Ex vivo total memory CD4+ T cells		CATSDGQGTDTQYF	global	4.90E-07	0.83	0.21	1	0.45
CLUSTER_4	S%GQGTDT_DKQR	18344	CASSRGQGTDTQYF	TRBV13-1	TRBJ2-3	PT7	2	AC GBS	Blood	Ex vivo total memory CD4+ T cells		CASSRGQGTDTQYF	global	4.90E-07	0.83	0.21	1	0.45
CLUSTER_4	S%GQGTDT_DKQR	57776	CASSKGQGTDTQYF	TCRBV05-04	TCRBJ02-03	PT11	1	AC GBS	Blood	Ex vivo total memory CD4+ T cells		CASSkGQGTDTQYF	global	4.90E-07	0.83	0.21	1	0.45
CLUSTER_4	S%GQGTDT_DKQR	59315	CASSRGQGTDTQYF	TCRBV06-06	TCRBJ02-03	PT11	1	AC GBS	Blood	Ex vivo total memory CD4+ T cells		CASSRGQGTDTQYF	global	4.90E-07	0.83	0.21	1	0.45
CLUSTER_4	S%GQGTDT_DKQR	59692	CASSQGQGTDTQYF	TCRBV04-01	TCRBJ02-03	PT12	1	AC GBS	Blood	Ex vivo total memory CD4+ T cells		CASSQGQGTDTQYF	global	4.90E-07	0.83	0.21	1	0.45
CLUSTER_5	LKQP_4_24	5	CASEPTRRGLKQPQHF	TCRBV19-01	TCRBJ02-02	PT10	1	AC GBS	Blood	P2-specific memory CD4+ T cell clone	CD4_130	CASeptrRGlkQPQHF	local	8.70E-05	0.00061	0.34	0.97	0.31
CLUSTER_5	LKQP_4_24	27781	CASIRQGALKQPQHF	TRBV5-8	TRBJ1-5	PT5	2	AC GBS	Blood	Ex vivo total memory CD4+ T cells		CASirQGalkQPQHF	local	8.70E-05	0.00061	0.34	0.97	0.31
CLUSTER_5	LKQP_4_24	15929	CASRTGVWLKQPQHF	TRBV28-1	TRBJ1-5	PT7	2	AC GBS	Blood	Ex vivo total memory CD4+ T cells		CASrTGvwlkQPQHF	local	8.70E-05	0.00061	0.34	0.97	0.31
CLUSTER_5	LKQP_4_24	69516	CATSRDLLKQPQHF	TCRBV15-01	TCRBJ01-05	PT12	1	AC GBS	Blood	Ex vivo total memory CD4+ T cells		CATSRdllkQPQHF	local	8.70E-05	0.00061	0.34	0.97	0.31
CLUSTER_6	RQG%YE_AGST	42603	CASRQGSYEQYF	TCRBV02-01	TCRBJ02-05	PT5	1	AC GBS	Blood	P2-specific memory CD4+ T cell clone	CD4_89	CASrQGSYEQYF	global	1.30E-08	9.30E-01	0.015	1	1
CLUSTER_6	RQG%YE_AGST	129	CASRQGSYEQYF	TCRBV02-01	TCRBJ02-07	PT1	1	REC GBS	Blood	Ex vivo total memory CD4+ T cells		CASrQGSYEQYF	global	1.30E-08	9.30E-01	0.015	1	1
CLUSTER_6	RQG%YE_AGST	49792	CSARQGSYEQYF	TCRBV20	TCRBJ02-07	PT5	1	REC GBS	Blood	Ex vivo total memory CD4+ T cells		CSARQGSYEQYF	global	1.30E-08	9.30E-01	0.015	1	1
CLUSTER_6	RQG%YE_AGST	59008	CASRQGGYEQYF	TCRBV07-02	TCRBJ02-07	PT5	1	REC GBS	Blood	Ex vivo total memory CD4+ T cells		CASrQGgYEQYF	global	1.30E-08	9.30E-01	0.015	1	1
CLUSTER_6	RQG%YE_AGST	71689	CSVRQGGYEQYF	TCRBV29-01	TCRBJ02-07	PT5	1	REC GBS	Blood	Ex vivo total memory CD4+ T cells		CSVrQGgYEQYF	global	1.30E-08	9.30E-01	0.015	1	1
CLUSTER_6	RQG%YE_AGST	86085	CASRQGGYEQYF	TCRBV10-03	TCRBJ02-07	PT5	1	REC GBS	Blood	Ex vivo total memory CD4+ T cells		CASrQGgYEQYF	global	1.30E-08	9.30E-01	0.015	1	1
CLUSTER_6	RQG%YE_AGST	99256	CSVRQGTYEQYF	TCRBV29-01	TCRBJ02-07	PT5	1	REC GBS	Blood	Ex vivo total memory CD4+ T cells		CSVrQGtYEQYF	global	1.30E-08	9.30E-01	0.015	1	1
CLUSTER_6	RQG%YE_AGST	100323	CASRQGAYEQYF	TCRBV07	TCRBJ02-07	PT5	1	REC GBS	Blood	Ex vivo total memory CD4+ T cells		CASrQGaYEQYF	global	1.30E-08	9.30E-01	0.015	1	1
CLUSTER_6	RQG%YE_AGST	112807	CSARQGAYEQYF	TCRBV20	TCRBJ02-07	PT5	1	REC GBS	Blood	Ex vivo total memory CD4+ T cells		CSARQGaYEQYF	global	1.30E-08	9.30E-01	0.015	1	1
CLUSTER_7	%TGGYE_AQST	4072	CANQTGGYEQYF	TCRBV06-05	TCRBJ02-05	PT10	11	AC GBS	Blood	P0-specific memory CD4+ T cell clone	CD4_42	CANqTGGYEQYF	global	6.60E-10	6.90E-01	0.007	0.078	1
CLUSTER_7	%TGGYE_AQST	20	CANQTGGYEQYF	TCRBV06-05	TCRBJ02-07	PT1	16	REC GBS	Blood	Ex vivo total memory CD4+ T cells		CANqTGGYEQYF	global	6.60E-10	6.90E-01	0.007	0.078	1
CLUSTER_7	%TGGYE_AQST	42622	CANQTGGYEQYF	TCRBV06-05	TCRBJ02-07	PT5	3	REC GBS	Blood	Ex vivo total memory CD4+ T cells		CANqTGGYEQYF	global	6.60E-10	6.90E-01	0.007	0.078	1
CLUSTER_7	%TGGYE_AQST	91325	CASSTGGYEQYF	TCRBV06-05	TCRBJ02-07	PT5	1	REC GBS	Blood	Ex vivo total memory CD4+ T cells		CASSTGGYEQYF	global	6.60E-10	6.90E-01	0.007	0.078	1
CLUSTER_7	%TGGYE_AQST	95151	CATSTGGYEQYF	TCRBV15-01	TCRBJ02-07	PT5	1	REC GBS	Blood	Ex vivo total memory CD4+ T cells		CATSTGGYEQYF	global	6.60E-10	6.90E-01	0.007	0.078	1
CLUSTER_7	%TGGYE_AQST	113796	CATATGGYEQFF	TCRBV18-01	TCRBJ02-01	PT5	1	REC GBS	Blood	Ex vivo total memory CD4+ T cells		CATaTGGyEQFF	global	6.60E-10	6.90E-01	0.007	0.078	1
CLUSTER_7	%TGGYE_AQST	116888	CANQTGGYEQYF	TCRBV06-05	TCRBJ02-07	PT7	3	REC GBS	Blood	Ex vivo total memory CD4+ T cells		CANqTGGYEQYF	global	6.60E-10	6.90E-01	0.007	0.078	1
CLUSTER_7	%TGGYE_AQST	145611	CASTTGGYEQFF	TCRBV19-01	TCRBJ02-01	PT7	1	REC GBS	Blood	Ex vivo total memory CD4+ T cells		CASTTGGyEQF	global	6.60E-10	6.90E-01	0.007	0.078	1
CLUSTER_7	%TGGYE_AQST	12783	CASSTGGYEQFF	TCRBV21-01	TCRBJ02-01	PT12	1	REC GBS	Blood	Ex vivo total memory CD4+ T cells		CASSTGGyEQFF	global	6.60E-10	6.90E-01	0.007	0.078	1
CLUSTER_8	%QGGNQP_AGQRST	76	CAWGQGGNQPQHF	TCRBV30-2	TCRBJ1-5	PT2	1	REC GBS	Blood	P0/CMV-specific memory CD4+ T cell clone	CD4_21	CAWGQGgNQPQHF	global	7.60E-11	0.14	0.002	0.33	0.095
CLUSTER_8	%QGGNQP_AGQRST	39151	CASRQGGNQPQHF	TRBV10-2	TRBJ1-5	PT1	3	AC GBS	Blood	Ex vivo total memory CD4+ T cells		CASrQGgNQPQHF	global	7.60E-11	0.14	0.002	0.33	0.095
CLUSTER_8	%QGGNQP_AGQRST	41063	CASSQGGNQPQHF	TRBV14-1	TRBJ1-5	PT1	3	AC GBS	Blood	Ex vivo total memory CD4+ T cells		CASSQGGNQPQHF	global	7.60E-11	0.14	0.002	0.33	0.095
CLUSTER_8	%QGGNQP_AGQRST	44525	CSVQQGGNQPQHF	TRBV29-1	TRBJ1-5	PT1	2	AC GBS	Blood	Ex vivo total memory CD4+ T cells		CSVqQGgNQPQHF	global	7.60E-11	0.14	0.002	0.33	0.095
CLUSTER_8	%QGGNQP_AGQRST	76795	CASTQGGNQPQHF	TCRBV19-01	TCRBJ01-05	PT2	1	AC GBS	Blood	Ex vivo total memory CD4+ T cells		CASTQGgNQPQHF	global	7.60E-11	0.14	0.002	0.33	0.095
CLUSTER_8	%QGGNQP_AGQRST	20678	CSVAQGGNQPQHF	TRBV29-1	TRBJ1-5	PT5	7	AC GBS	Blood	Ex vivo total memory CD4+ T cells		CSVaQGgNQPQHF	global	7.60E-11	0.14	0.002	0.33	0.095
CLUSTER_8	%QGGNQP_AGQRST	22429	CASSQGGNQPQHF	TRBV18-1	TRBJ1-5	PT5	4	AC GBS	Blood	Ex vivo total memory CD4+ T cells		CASSQGGNQPQHF	global	7.60E-11	0.14	0.002	0.33	0.095
CLUSTER_8	%QGGNQP_AGQRST	23449	CASSQGGNQPQHF	TRBV4-2	TRBJ1-5	PT5	3	AC GBS	Blood	Ex vivo total memory CD4+ T cells		CASSQGGNQPQHF	global	7.60E-11	0.14	0.002	0.33	0.095
CLUSTER_8	%QGGNQP_AGQRST	12563	CASRQGGNQPQHF	TRBV6-5	TRBJ1-5	PT7	2	AC GBS	Blood	Ex vivo total memory CD4+ T cells		CASrQGgNQPQHF	global	7.60E-11	0.14	0.002	0.33	0.095
CLUSTER_8	%QGGNQP_AGQRST	59827	CASRQGGNQPQHF	TCRBV06-1	TCRBJ01-05	PT12	13	AC GBS	Blood	Ex vivo total memory CD4+ T cells		CASrQGgNQPQHF	global	7.60E-11	0.14	0.002	0.33	0.095
CLUSTER_8	%QGGNQP_AGQRST	67377	CASSQGGNQPQHF	TCRBV03-01/03-02	TCRBJ01-05	PT12	1	AC GBS	Blood	Ex vivo total memory CD4+ T cells		CASSQGGNQPQHF	global	7.60E-11	0.14	0.002	0.33	0.095
CLUSTER_8	%QGGNQP_GS	92628	CASSQGGNQPQHF	TCRBV03-01/03-02	TCRBJ01-05	PT2	1	REC GBS	Blood	Ex vivo total memory CD4+ T cells		CASSQGGNQPQHF	global	6.80E-08	0.4	0.5	1	0.093
CLUSTER_8	%QGGNQP_GS	100035	CAAGQGGNQPQHF	TCRBV07-02	TCRBJ01-05	PT2	1	REC GBS	Blood	Ex vivo total memory CD4+ T cells		CAaGQGgNQPQHF	global	6.80E-08	0.4	0.5	1	0.093
CLUSTER_8	GQG%NQP_AGST	166281	CSAGQGSNQPQHF	TCRBV20	TCRBJ01-05	PT4	1	REC GBS	Blood	Ex vivo total memory CD4+ T cells		CSAGQGSNQPQHF	global	4.30E-09	0.77	0.022	0.76	0.18
CLUSTER_8	GQG%NQP_AGST	173590	CANGQGTNQPQHF	TCRBV06-05	TCRBJ01-05	PT4	1	REC GBS	Blood	Ex vivo total memory CD4+ T cells		CANGQGtNQPQHF	global	4.30E-09	0.77	0.022	0.76	0.18
CLUSTER_8	GQG%NQP_AGST	179908	CSVGQGANQPQHF	TCRBV29-01	TCRBJ01-05	PT4	1	REC GBS	Blood	Ex vivo total memory CD4+ T cells		CSVGQGaNQPQHF	global	4.30E-09	0.77	0.022	0.76	0.18
CLUSTER_8	%QGGNQP_GS	35467	CSASQGGNQPQHF	TCRBV20-01	TCRBJ01-05	PT7	1	REC GBS	Blood	Ex vivo total memory CD4+ T cells		CSASQGgNQPQHF	global	6.80E-08	0.4	0.5	1	0.093
CLUSTER_8	GQG%NQP_AGST	57559	CSAGQGSNQPQHF	TCRBV20	TCRBJ01-05	PT9	4	REC GBS	Blood	Ex vivo total memory CD4+ T cells		CSAGQGSNQPQHF	global	4.30E-09	0.77	0.022	0.76	0.18
CLUSTER_8	G%GGNQP_EQ	78526	CAWGEGGNQPQHF	TCRBV30-01	TCRBJ01-05	PT12	1	REC GBS	Blood	Ex vivo total memory CD4+ T cells		CAWGeGGNQPQHF	global	2.70E-08	0.73	0.35	1	0.033
CLUSTER_8	%QGGNQP_GS	51568	CSAGQGGNQPQHF	TCRBV29-01	TCRBJ01-05	PT13	1	REC GBS	Blood	Ex vivo total memory CD4+ T cells		CSAGQGgNQPQHF	global	6.80E-08	0.4	0.5	1	0.093
CLUSTER_9	SM%YE_AGNQV	21	CAWSMQYEQYF	TCRBV30-2	TCRBJ2-7	PT2	1	REC GBS	Blood	P0/CMV-specific memory CD4+ T cell clone	CD4_13	CAWSmqYEQYF	global	1.20E-08	0.048	0.13	0.67	0.095
CLUSTER_9	SM%YE_AGNQV	2789	CASSMNYEQYF	TCRBV02-01	TCRBJ02-07	PT1	1	RECGBS	Blood	Ex vivo total memory CD4+ T cells		CASSmnYEQYF	global	1.20E-08	0.048	0.13	0.67	0.095
CLUSTER_9	SM%YE_AGNQV	104846	CASSMGYEQYF	TCRBV02-01	TCRBJ02-07	PT2	1	RECGBS	Blood	Ex vivo total memory CD4+ T cells		CASSmgYEQYF	global	1.20E-08	0.048	0.13	0.67	0.095
CLUSTER_9	SM%YE_AGNQV	16975	CASSMVYEQYF	TCRBV19-01	TCRBJ02-07	PT7	2	RECGBS	Blood	Ex vivo total memory CD4+ T cells		CASSmvYEQYF	global	1.20E-08	0.048	0.13	0.67	0.095
CLUSTER_9	SM%YE_AGNQV	47646	CASSMAYEQFF	TCRBV07-02	TCRBJ02-01	PT13	1	RECGBS	Blood	Ex vivo total memory CD4+ T cells		CASSmayEQFF	global	1.20E-08	0.048	0.13	0.67	0.095
CLUSTER_10	SL%NT_GLSTV	82206	CASSLSNTEAF	TRBV11-2*01	TCRBJ01-03	PT2	1	CFSElow_AA	Blood CFSE low	P0/P2/PMP22-specific memory CD4+ T cell clone		CASSLsnteaF	global	5.30E-09	0.16	0.01	0.95	0.38
CLUSTER_10	SLS%T_DEGNR	75299	CASSLSETQYF	TCRBV05-01	TCRBJ02-05	PT2	2	AC GBS	Blood	Ex vivo total memory CD4+ T cells		CASSLsETQYF	global	2.90E-08	0.23	0.23	0.28	0.31
CLUSTER_10	SL%NT_GLSTV	27114	CASSLVNTIYF	TRBV13-1	TRBJ1-3	PT5	2	AC GBS	Blood	Ex vivo total memory CD4+ T cells		CASSLvNTIYF	global	5.30E-09	0.16	0.01	0.95	0.38
CLUSTER_10	SL%NT_GLSTV	27581	CASSLGNTIYF	TRBV12-3/12-4	TRBJ1-3	PT5	2	AC GBS	Blood	Ex vivo total memory CD4+ T cells		CASSLGNTIYF	global	5.30E-09	0.16	0.01	0.95	0.38
CLUSTER_10	SLS%T_DEGNR	19420	CASSLSGTQYF	TRBV28-1	TRBJ2-5	PT5	11	AC GBS	Blood	Ex vivo total memory CD4+ T cells		CASSLSGTQYF	global	2.90E-08	0.23	0.23	0.28	0.31
CLUSTER_10	SLS%T_DEGNR	21564	CASSLSDTQYF	TRBV19-1	TRBJ2-3	PT5	5	AC GBS	Blood	Ex vivo total memory CD4+ T cells		CASSLsDTQYF	global	2.90E-08	0.23	0.23	0.28	0.31
CLUSTER_10	SL%NT_GLSTV	17188	CASSLTNTIYF	TRBV7-9	TRBJ1-3	PT7	2	AC GBS	Blood	Ex vivo total memory CD4+ T cells		CASSLtNTIYF	global	5.30E-09	0.16	0.01	0.95	0.38
CLUSTER_10	SL%NT_GLSTV	17517	CASSLTNTIYF	TRBV5-8	TRBJ1-3	PT7	2	AC GBS	Blood	Ex vivo total memory CD4+ T cells		CASSLtNTIYF	global	5.30E-09	0.16	0.01	0.95	0.38
CLUSTER_10	SLS%T_DEGNR	7198	CASSLSETQYF	TRBV7-9	TRBJ2-5	PT7	4	AC GBS	Blood	Ex vivo total memory CD4+ T cells		CASSLsETQYF	global	2.90E-08	0.23	0.23	0.28	0.31
CLUSTER_10	SL%NT_GLSTV	72250	CASSLLNTIYF	TCRBV05-05	TCRBJ01-03	PT10	2	AC GBS	Blood	Ex vivo total memory CD4+ T cells		CASSLlNTIYF	global	5.30E-09	0.16	0.01	0.95	0.38
CLUSTER_10	SL%NT_GLSTV	51493	CASSLLNTIYF	TCRBV05-06	TCRBJ01-03	PT11	3	AC GBS	Blood	Ex vivo total memory CD4+ T cells		CASSLlNTIYF	global	5.30E-09	0.16	0.01	0.95	0.38
CLUSTER_10	SL%NT_GLSTV	52604	CASSLGNTIYF	TCRBV05-04	TCRBJ01-03	PT11	1	AC GBS	Blood	Ex vivo total memory CD4+ T cells		CASSLGNTIYF	global	5.30E-09	0.16	0.01	0.95	0.38
CLUSTER_10	SL%NT_GLSTV	68182	CASSLLNTIYF	TCRBV28-01	TCRBJ01-03	PT12	1	AC GBS	Blood	Ex vivo total memory CD4+ T cells		CASSLlNTIYF	global	5.30E-09	0.16	0.01	0.95	0.38
CLUSTER_10	SLS%T_DENR	79901	CASSLSETQYF	TCRBV05-01	TCRBJ02-05	PT2	5	RECGBS	Blood	Ex vivo total memory CD4+ T cells		CASSLsETQYF	global	2.90E-10	0.28	0.011	0.2	0.11
CLUSTER_10	SLS%T_DENR	89853	CASSLSDTQYF	TCRBV05-06	TCRBJ02-03	PT2	1	RECGBS	Blood	Ex vivo total memory CD4+ T cells		CASSLsDTQYF	global	2.90E-10	0.28	0.011	0.2	0.11
CLUSTER_10	SLS%T_DENR	7720	CASSLSETQYF	TCRBV07-09	TCRBJ02-05	PT7	5	RECGBS	Blood	Ex vivo total memory CD4+ T cells		CASSLsETQYF	global	2.90E-10	0.28	0.011	0.2	0.11
CLUSTER_10	SLS%T_DENR	73916	CASSLSRTQHF	TCRBV28-01	TCRBJ01-05	PT12	1	RECGBS	Blood	Ex vivo total memory CD4+ T cells		CASSLsrtQHF	global	2.90E-10	0.28	0.011	0.2	0.11
CLUSTER_10	SLS%T_DENR	44532	CASSLSETQYF	TCRBV05-01	TCRBJ02-05	PT13	1	RECGBS	Blood	Ex vivo total memory CD4+ T cells		CASSLsETQYF	global	2.90E-10	0.28	0.011	0.2	0.11
CLUSTER_10	SLS%T_DENR	49371	CASSLSDTQYF	TCRBV05-01	TCRBJ02-03	PT13	1	RECGBS	Blood	Ex vivo total memory CD4+ T cells		CASSLsDTQYF	global	2.90E-10	0.28	0.011	0.2	0.11
CLUSTER_10	SLS%T_DENR	50504	CASSLSNTQYF	TCRBV05-06	TCRBJ02-03	PT13	1	RECGBS	Blood	Ex vivo total memory CD4+ T cells		CASSLsnTQYF	global	2.90E-10	0.28	0.011	0.2	0.11
CLUSTER_11	S%GG_AGV	111585	CATSAGGEQF	TRBV24-1*02	TCRBJ01-03	PT16	1	CFSElow_AA	Blood CFSE low	P0/P2/PMP22-specific memory CD4+ T cell clone		CATSAGGeqF	global	8.40E-07	0.32	0.36	1	1
CLUSTER_11	S%GG_AGV	96079	CASSGGGAFF	TCRBV05-08	TCRBJ01-01	PT2	1	REC GBS	Blood	Ex vivo total memory CD4+ T cells		CASSGGgAFF	global	8.40E-07	0.32	0.36	1	1
CLUSTER_11	S%GG_AGV	97626	CASSVGGYTF	TCRBV02-01	TCRBJ01-02	PT2	1	REC GBS	Blood	Ex vivo total memory CD4+ T cells		CASSVgGYTF	global	8.40E-07	0.32	0.36	1	1
CLUSTER_11	S%GG_AGV	9054	CASSVGGRIF	TCRBV27-01	TCRBJ01-04	PT7	1	REC GBS	Blood	Ex vivo total memory CD4+ T cells		CASSVgGRiF	global	8.40E-07	0.32	0.36	1	1
CLUSTER_12	GGSP_4_22	93	CSAPAGGGSPLHF	TCRBV20	TCRBJ01-06	PT7	2	AC GBS	Blood	PMP22-specific memory CD4+ T cell clone	CD4_149	CSApAGGgSPLHF	local	3.60E-11	5.20E-36	0.001	0.97	1
CLUSTER_12	GGSP_4_22	2739	CASSFLFQGGSPLHF	TCRBV06-02/06-03	TCRBJ01-06	PT10	218	AC GBS	CSF	In vitro expanded CD4+ T cells		CASSflfQGgSPLHF	local	3.60E-11	5.20E-36	0.001	0.97	1
CLUSTER_12	GGSP_4_22	1361	CATSRDPQGGSPLHF	TCRBV15-01	TCRBJ01-06	PT11	10	AC GBS	CSF	In vitro expanded CD4+ T cells		CATSRdpQGgSPLHF	local	3.60E-11	5.20E-36	0.001	0.97	1

CLUSTER_12	GGSP_4_22	2340	CATSRDPQGGSPLHF	TCRBV15-01	TCRBJ01-06	PT11	10	AC GBS	CSF	In vitro expanded CD4+ T cells		CATSRdpQGgSPLHF	local	3.60E-11	5.20E-36	0.001	0.97	1
CLUSTER_12	GGSP_4_22	141	CASSYRGRGGSPLHF	TCRBV12-03/12-04	TCRBJ01-06	PT12	1	AC GBS	CSF	In vitro expanded CD4+ T cells		CASSYRGrggSPLHF	local	3.60E-11	5.20E-36	0.001	0.97	1
CLUSTER_12	GGSP_4_22	80	CASSGKGPGGGSPLHF	TCRBV09-01	TCRBJ01-06	PT16	346	AC GBS	Nerve biopsy	In vitro expanded CD4+ T cells		CASSgkgpGGgSPLHF	local	3.60E-11	5.20E-36	0.001	0.97	1
CLUSTER_12	GGSP_4_22	82	CARSGKGQGGGSPLHF	TCRBV09-01	TCRBJ01-06	PT16	29	AC GBS	Nerve biopsy	In vitro expanded CD4+ T cells		CArsgkGQGggSPLHF	local	3.60E-11	5.20E-36	0.001	0.97	1
CLUSTER_12	GGSP_4_22	100	CARSGTGPGGGSPLHF	TCRBV09-01	TCRBJ01-06	PT16	19	AC GBS	Nerve biopsy	In vitro expanded CD4+ T cells		CArsGTGpgggSPLHF	local	3.60E-11	5.20E-36	0.001	0.97	1
CLUSTER_12	GGSP_4_22	85	CARRGTGPGGGSPLHF	TCRBV09-01	TCRBJ01-06	PT16	18	AC GBS	Nerve biopsy	In vitro expanded CD4+ T cells		CArrGTGpgggSPLHF	local	3.60E-11	5.20E-36	0.001	0.97	1
CLUSTER_12	GGSP_4_22	86	CARRGKGQGGGSPLHF	TCRBV09-01	TCRBJ01-06	PT16	9	AC GBS	Nerve biopsy	In vitro expanded CD4+ T cells		CArrgkGQGggSPLHF	local	3.60E-11	5.20E-36	0.001	0.97	1
CLUSTER_12	GGSP_4_22	106	CASSGTGPGGGSPLHF	TCRBV09-01	TCRBJ01-06	PT16	8	AC GBS	Nerve biopsy	In vitro expanded CD4+ T cells		CASSGTGpgggSPLHF	local	3.60E-11	5.20E-36	0.001	0.97	1
CLUSTER_12	GGSP_4_22	104	CASRGKGPGGGSPLHF	TCRBV09-01	TCRBJ01-06	PT16	6	AC GBS	Nerve biopsy	In vitro expanded CD4+ T cells		CASRGkgpgggSPLHF	local	3.60E-11	5.20E-36	0.001	0.97	1
CLUSTER_12	GGSP_4_22	141	CASRGTGPGGGSPLHF	TCRBV09-01	TCRBJ01-06	PT16	6	AC GBS	Nerve biopsy	In vitro expanded CD4+ T cells		CASrGTGpgggSPLHF	local	3.60E-11	5.20E-36	0.001	0.97	1
CLUSTER_12	GGSP_4_22	123	CASRGTGQGGGSPLHF	TCRBV09-01	TCRBJ01-06	PT16	6	AC GBS	Nerve biopsy	In vitro expanded CD4+ T cells		CASrGTGqgggSPLHF	local	3.60E-11	5.20E-36	0.001	0.97	1
CLUSTER_12	GGSP_4_22	152	CARRGKGPGGGSPLHF	TCRBV09-01	TCRBJ01-06	PT16	3	AC GBS	Nerve biopsy	In vitro expanded CD4+ T cells		CArRGkgpgggSPLHF	local	3.60E-11	5.20E-36	0.001	0.97	1
CLUSTER_12	GGSP_4_22	91	CPSSGKGQGGGSPLHF	TCRBV09-01	TCRBJ01-06	PT16	3	AC GBS	Nerve biopsy	In vitro expanded CD4+ T cells		CpssgkGQGggSPLHF	local	3.60E-11	5.20E-36	0.001	0.97	1
CLUSTER_12	GGSP_4_22	120	CAPSGKGQGGGSPLHF	TCRBV09-01	TCRBJ01-06	PT16	2	AC GBS	Nerve biopsy	In vitro expanded CD4+ T cells		CApsgkGQGggSPLHF	local	3.60E-11	5.20E-36	0.001	0.97	1
CLUSTER_12	GGSP_4_22	48	CASSGTGQGGGSPLHF	TCRBV09-01	TCRBJ01-06	PT16	2	AC GBS	Nerve biopsy	In vitro expanded CD4+ T cells		CASSGTGqgggSPLHF	local	3.60E-11	5.20E-36	0.001	0.97	1
CLUSTER_12	GGSP_4_22	150	CAPRGTGPGGGSPLHF	TCRBV09-01	TCRBJ01-06	PT16	1	AC GBS	Nerve biopsy	In vitro expanded CD4+ T cells		CAprGTGpgggSPLHF	local	3.60E-11	5.20E-36	0.001	0.97	1
CLUSTER_12	GGSP_4_22	149	CAPSGKGPGGGSPLHF	TCRBV09-01	TCRBJ01-06	PT16	1	AC GBS	Nerve biopsy	In vitro expanded CD4+ T cells		CApSGkgpgggSPLHF	local	3.60E-11	5.20E-36	0.001	0.97	1
CLUSTER_12	GGSP_4_22	148	CAPSGTGPGGGSPLHF	TCRBV09-01	TCRBJ01-06	PT16	1	AC GBS	Nerve biopsy	In vitro expanded CD4+ T cells		CApsGTGpgggSPLHF	local	3.60E-11	5.20E-36	0.001	0.97	1
CLUSTER_12	GGSP_4_22	145	CASCGKGQGGGSPLHF	TCRBV09-01	TCRBJ01-06	PT16	1	AC GBS	Nerve biopsy	In vitro expanded CD4+ T cells		CAScgkGQGggSPLHF	local	3.60E-11	5.20E-36	0.001	0.97	1
CLUSTER_12	GGSP_4_22	143	CASCGTGQGGGSPLHF	TCRBV09-01	TCRBJ01-06	PT16	1	AC GBS	Nerve biopsy	In vitro expanded CD4+ T cells		CAScGTGqgggSPLHF	local	3.60E-11	5.20E-36	0.001	0.97	1
CLUSTER_12	GGSP_4_22	3736	CAWSAGTGGSPLHF	TCRBV30-01	TCRBJ01-06	PT16	1	AC GBS	Blood CFSE low	P0/P2/PMP22-specific central memory CD4+ T cells		CAWSaGTGGSPLHF	local	3.60E-11	5.20E-36	0.001	0.97	1
CLUSTER_12	GGSP_4_22	103	CPPRGTGPGGGSPLHF	TCRBV09-01	TCRBJ01-06	PT16	1	AC GBS	Nerve biopsy	In vitro expanded CD4+ T cells		CpprGTGpgggSPLHF	local	3.60E-11	5.20E-36	0.001	0.97	1
CLUSTER_12	GGSP_4_22	99	CPRRGKGQGGGSPLHF	TCRBV09-01	TCRBJ01-06	PT16	1	AC GBS	Nerve biopsy	In vitro expanded CD4+ T cells		CprrgkGQGggSPLHF	local	3.60E-11	5.20E-36	0.001	0.97	1
CLUSTER_12	GGSP_4_22	89	CPRRGTGPGGGSPLHF	TCRBV09-01	TCRBJ01-06	PT16	1	AC GBS	Nerve biopsy	In vitro expanded CD4+ T cells		CprrGTGpgggSPLHF	local	3.60E-11	5.20E-36	0.001	0.97	1
CLUSTER_12	GGSP_4_22	103	CPSRGKGPGGGSPLHF	TCRBV09-01	TCRBJ01-06	PT16	1	AC GBS	Nerve biopsy	In vitro expanded CD4+ T cells		CpsRGkgpgggSPLHF	local	3.60E-11	5.20E-36	0.001	0.97	1
CLUSTER_12	GGSP_4_22	100	CPSRGKGQGGGSPLHF	TCRBV09-01	TCRBJ01-06	PT16	1	AC GBS	Nerve biopsy	In vitro expanded CD4+ T cells		CpsrgkGQGggSPLHF	local	3.60E-11	5.20E-36	0.001	0.97	1

*Clonotype ID from Supplementary Table 1
