# Supplementary material for: Autoreactive T cells target peripheral nerves in Guillain–Barré syndrome
Source: Nature. 2024 Jan 17;626(7997):160–8. doi: 10.1038/s41586-023-06916-6 (PMC10830418; doi:10.1038/s41586-023-06916-6)
Supplement: Supplementary file 2 — Reporting Summary [file 41586_2023_6916_MOESM2_ESM.pdf]

nature portfolio

Corresponding author(s): Daniela Latorre

Last updated by author(s): Nov 14, 2023

Reporting Summary

Nature Portfolio wishes to improve the reproducibility of the work that we publish. This form provides structure for consistency and transparency in reporting. For further information on Nature Portfolio policies, see our Editorial Policies and the Editorial Policy Checklist.

Statistics

For all statistical analyses, confirm that the following items are present in the figure legend, table legend, main text, or Methods section.

n/a	Confirmed	
☐	☒	The exact sample size (n) for each experimental group/condition, given as a discrete number and unit of measurement
☐	☒	A statement on whether measurements were taken from distinct samples or whether the same sample was measured repeatedly
☐	☒	The statistical test(s) used AND whether they are one- or two-sided<br>*Only common tests should be described solely by name; describe more complex techniques in the Methods section.*
☒	☐	A description of all covariates tested
☒	☐	A description of any assumptions or corrections, such as tests of normality and adjustment for multiple comparisons
☐	☒	A full description of the statistical parameters including central tendency (e.g. means) or other basic estimates (e.g. regression coefficient) AND variation (e.g. standard deviation) or associated estimates of uncertainty (e.g. confidence intervals)
☐	☒	For null hypothesis testing, the test statistic (e.g. F, t, r) with confidence intervals, effect sizes, degrees of freedom and P value noted<br>*Give P values as exact values whenever suitable.*
☒	☐	For Bayesian analysis, information on the choice of priors and Markov chain Monte Carlo settings
☒	☐	For hierarchical and complex designs, identification of the appropriate level for tests and full reporting of outcomes
☒	☐	Estimates of effect sizes (e.g. Cohen's d, Pearson's r), indicating how they were calculated

Our web collection on statistics for biologists contains articles on many of the points above.

Software and code

Policy information about availability of computer code

Data collection	BD FACS Diva (v9.0) was used for acquisition of samples. The Microbeta2 Windows Workstation software (v2.3.0.12) was used to acquire data of T cell proliferation by [3H]-thymidine incorporation on a Beta counter 2 (Perkin Elmer).
Data analysis	IMGT (v3.5.31) was used to analyze TCR Vβ sequences obtained by Sanger method. ImmunoSEQ Analyzer V3.0 (http://www.immunoseq.com) was used for data processing of TCR Vβ CDR3 sequencing performed by Adaptive Biotechnologies. The R package immunarch 0.9.0 (https://github.com/immunomind/immunarch) was used to study antigen-specific clonotypes in each donor's repertoire according to bioidentity overlap, defined as identical identified V gene, amino acid sequence of the CDR3 region and identified J gene. The HetzDra/turboGliph 0.99.2 R package (https://github.com/HetzDra/turboGliph/) was used to group lymphocyte interaction by paratope hotspots and predict TCRβ specificity clusters. Graphpad Prism (v9) was used to analyze data and create plots. Seurat (v 4.9.9.9059) was used for the analysis of scRNAseq experiments. NetMHCIIpan 4.0 server was used for peptide-HLA affinity prediction. Data Analysis Software Suite for LEGENDplex™ (Biolegend) was used for the quantification cytokine release by autoreactive T cell clones. Flow-Jo (v10.8.1) was used for flow cytometry data analysis. Gene set enrichment analysis (GSEA) was performed using the software package "escape" (version 1.10.0, https://github.com/ncborcherding/escape). The R version used for all the computational analyis is v 4.2.1..

For manuscripts utilizing custom algorithms or software that are central to the research but not yet described in published literature, software must be made available to editors and reviewers. We strongly encourage code deposition in a community repository (e.g. GitHub). See the Nature Portfolio guidelines for submitting code & software for further information.

Data

Policy information about availability of data

All manuscripts must include a data availability statement. This statement should provide the following information, where applicable:

- Accession codes, unique identifiers, or web links for publicly available datasets
- A description of any restrictions on data availability
- For clinical datasets or third party data, please ensure that the statement adheres to our policy

Publicly available datasets included in the study are available at the following web links: https://doi.org/10.21417/JSL2021S ; https://doi.org/10.21417/AC2020EJI and https://doi.org/10.21417/B73H0P, https://vdjdb.cdr3.net/search, https://www.ncbi.nlm.nih.gov/geo/query/acc.cgi (accession numbers GSE59114;, GSE126030, GSE131935, GSE104024, GSE193442) and https://ega-archive.org/ (accession numbers EGAS00001003215, EGAD00001005290. The data presented in this manuscript are tabulated in the main paper and in the supplementary materials. TCR Vβ sequences from samples listed in Extended Data Table 2 are deposited in the immuneACCESS database (https://www.immunoseq.com/immuneaccess/, DOI: https://doi.org/10.21417/LS2023N).

Human research participants

Policy information about studies involving human research participants and Sex and Gender in Research.

Reporting on sex and gender	The study cohort was comprised of both sexes as shown in Extended Data Table 1. Patients were included in the study as samples become available and sexes were included whenever possible.
Population characteristics	Relevant information on human research participants such as age, gender, disease, HLA-typing and anti-gangliosides antibody presence are provided in Extended Data Table 1 and Supplementary Table 3. Specifically, the study included 20 GBS patients (age range 30-50, 59.5 ± 13.3 (mean ± SD)) and 5 CMT1 patients (age range 23-45, 32 ± 8.4 (mean ± SD)) recruited from University Hospital Zurich and Cantonal Hospital of Lugano (EOC), and 21 HD obtained from the Swiss Blood Donation Center of Lugano (n = 15, age range 23-51, 47.5 ± 15.1 (mean ± SD)) and from the CoV-ETH study (n = 6, age range 38-50, 41.3 ± 4.6 (mean ± SD)). We included a total of 16 patients with AIDP, both at acute phase (range 7 - 36 days from disease onset, 12.7 ± 9.9 (mean ± SD)), and/or at follow-up visits during the recovery stage (range 135-509 days from disease onset, 244.6 ± 115.7 (mean ± SD)) (Extended Data Table 1). We also included AMAN patients (n = 4, all associated with preceding gastroenteritis) at disease onset (n=3, range 4-8 days from disease onset 5.7 ± 2.1 (mean ± SD) or recovery (n=1, 1520 days after disease onset), or CMT1 patients (n= 3 CMT1A and n=2 CMT1X).
Recruitment	Recruitment of patients was biased by the study design. Given the large clinical heterogeneity of GBS disease, the study specifically aimed at characterizing autoreactive T cell responses initially in matched acute/recovery samples from a defined subset of GBS patients, specifically suffering from the AIDP subtype. The AIDP patients were included in the study based on the diagnosis when matched acute/recovery samples become available. AMAN and CMT patients were included later based on the diagnosis as they become available. Diagnosis of AIDP, AMAN or CMT was based on the criteria of the National Institute of Neurological Disorders and Stroke (NINDS). Biological samples were collected at acute phase and/or at follow-up visits during the recovery stage (Extended Data Table 1). All patient samples were recruited from University Hospital Zurich and Cantonal Hospital of Lugano (EOC). Healthy donors were recruited at the Swiss Blood Donation Center of Lugano and post-COVID-19 healthy donors were recruited from the CoV-ETH cohort study and used as control group in all experiments.
Ethics oversight	The study was approved by the Ethical committees of Zurich (NeuroMyoCyTOF study, BASEC-Nr: 2016-00929; CoV-ETH cohort, BASEC-Nr. 2020-00949) and Lugano (IGOS study, BASEC-Nr: 2018-01860). All participants provided written informed consent for participation in the study.

Note that full information on the approval of the study protocol must also be provided in the manuscript.

Field-specific reporting

Please select the one below that is the best fit for your research. If you are not sure, read the appropriate sections before making your selection.

☒ Life sciences ☐ Behavioural & social sciences ☐ Ecological, evolutionary & environmental sciences

For a reference copy of the document with all sections, see nature.com/documents/nr-reporting-summary-flat.pdf

Life sciences study design

All studies must disclose on these points even when the disclosure is negative.

Sample size	AIDP (n=16), AMAN (n=4), CMT (n=5) patients and HD (n= 21). No statistical methods were used to predetermine sample size. GBS is a rare disease and we included in the study patients enrolled in different centers in Switzerland and based on the diagnosis of AIDP, AMAN or CMT subtype. Sample size was chosen based our previous studies (i.e. Narcolepsy study, Latorre et al, Nature, 2018) as well as on the clear evidence of significant enrichment of autoreactive T cells in GBS patients compared to healthy individuals.
Data exclusions	Data from bulk TCR sequencing of CD4+ memory T cells ex vivo from the blood of GBS patients were excluded from the calculation of the

Data exclusions	cumulative frequency (Fig 4d and h) if they had less than 5500 clonotypes (identified as productive rearrangements). In scRNAseq data analysis, on the basis of QC exclusion was done by filtering of low-quality cells and cell doublets or multiples, and cells with >5% mitochondrial counts, we normalized the data and performed scaling, dimensionality reduction and clustering on the top 2000 highly variable features in the dataset (Seurat v 4.9.9.9059).
Replication	In the cases of T cell clones, their reactivity against self-antigens was mostly assessed in multiple occasions (in at least 2 separate experiments) with reproducible results.
Randomization	Randomization was not possible given the experimental design. The AIDP patients were included in the study based on the diagnosis when matched acute/recovery samples become available. AMAN and CMT patients were included later based on the diagnosis. The requisite for inclusion in the study was based on the diagnosis of AIDP, AMAN and CMT according to the criteria of the National Institute of Neurological Disorders and Stroke (NINDS).
Blinding	Investigators were not blinded to allocation during experiments and outcome assessment due to the experimental design of the study and the low likelihood of bias in the particular experiments. Given the large clinical heterogeneity of GBS disease, the study specifically aimed at characterizing autoreactive T cell responses in matched acute/recovery samples from a defined subset of GBS patients, specifically suffering from the AIDP subtype. AMAN and CMT patients and post-COVID-19 healthy donors were included later in the study. The investigators were unaware of the initial infectious trigger of the disease in the case on AIDP and AMAN patients. Healthy donors were specifically included as control groups in all experiments.

Reporting for specific materials, systems and methods

We require information from authors about some types of materials, experimental systems and methods used in many studies. Here, indicate whether each material, system or method listed is relevant to your study. If you are not sure if a list item applies to your research, read the appropriate section before selecting a response.

Materials & experimental systems

n/a	Involved in the study	
☐	☒	Antibodies
☒	☐	Eukaryotic cell lines
☒	☐	Palaeontology and archaeology
☒	☐	Animals and other organisms
☒	☐	Clinical data
☒	☐	Dual use research of concern

Methods

n/a	Involved in the study	
☒	☐	ChIP-seq
☐	☒	Flow cytometry
☒	☐	MRI-based neuroimaging

Antibodies

Antibodies used

CD3 BV785 Cat:300472; Clone:UCHT1; Biolegend
CD8 APC-Fire 750; Cat:301066; Clone:RPA-T8; Biolegend
CD8 FITC Cat: A07756 ; Clone: B9.11; Beckman Coulter
CD4 PE/Dazzle 594; Cat:300548; Clone:RPA-T4; Biolegend
CD45RA BV650 Cat:304136; Clone:HI100; Biolegend
CCR7 BV421 Cat:353208; Clone:G043H7; Biolegend
CD25 PC5 Cat:IM2646; Clone:B1.49.9; Beckman Coulter
CD56 PC5 Cat:A07789; Clone:N901; Beckman Coulter
CD14 PC5 Cat:A70204; Beckman Coulter
CD19 FITC Cat:555412; Clone:HIB19; BD Biosciences
ICOS Pacific Blue Cat:313522; Clone:H4A3; Biolegend
CD25 PE Cat:555432; Clone:M-A251; BD Biosciences
Granzyme A APC Cat:507220; Clone CB9; Biolegend
Granzyme B PE Cat:396406; Clone QA18A28; Biolegend
Perforin APC/Cyanine7 Cat:308128; Clone dG9; Biolegend
TNFα BV785 Cat:502948; Clone MAb11; Biolegend
IL-10 PE/Cyanine7 Cat:501404; Clone JES3-9D7; Biolegend
IL-17A BV605 Cat:512326; Clone BL168; Biolegend
IFNγ BUV395 Cat:563563; Clone B27; BD Biosciences
IL-4 BV711 Cat:564112; Clone MP4-25D2; BD Biosciences
IL-22 BUV737 Cat:367-7229-42; Clone 22URTI; Thermo Fisher Scientific

antiHLA-DR, clone L243, ATCC cat n HB55, produced in house from hybridoma cell line
anti-HLA-DQ clone SPVL3 (1), produced in house from hybridoma cell line
anti-HLA-DP, clone B7/21 (2), produced in house from hybridoma cell line

(1) H. Spits, G. Keizer, J. Borst, C. Terhorst, A. Hekman, J. E. de Vries, Characterization of monoclonal antibodies against cell surface molecules associated with cytotoxic activity of natural and activated killer cells and cloned CTL lines. Hybridoma 2, 423–437 (1983). doi:10.1089/hyb.1983.2.423 Medline
(2) A. J. Watson, R. DeMars, I. S. Trowbridge, F. H. Bach, Detection of a novel human class II HLA antigen. Nature 304, 358–361 (1983). doi:10.1038/304358a0 Medline

Validation

Commercial antibodies were used following the manufacturer's instructions. Anti-HLA neutralizing antibodies were validated by detecting expected blocking of proliferation on T cell clones with known HLA restriction.

CD3 BV785 Cat:300472; Clone:UCHT1; Biolegend: https://www.biolegend.com/fr-ch/products/brilliant-violet-785-anti-human-cd3-antibody-14454
CD8 APC-Fire 750; Cat:301066; Clone:RPA-T8; Biolegend: https://www.biolegend.com/fr-ch/products/apc-fire-750-anti-human-cd8a-antibody-13580
CD8 FITC Cat: A07756 ; Clone: B9.11; Beckman Coulter: https://www.beckman.de/reagents/coulter-flow-cytometry/antibodies-and-kits/single-color-antibodies/cd8/A07756
CD4 PE/Dazzle 594; Cat:300548; Clone:RPA-T4; Biolegend: https://www.biolegend.com/fr-ch/products/pe-dazzle-594-anti-human-cd4-antibody-9780
CD45RA BV650 Cat:304136; Clone:HI100; Biolegend: https://www.biolegend.com/fr-ch/products/brilliant-violet-650-anti-human-cd45ra-antibody-7662
CCR7 BV421 Cat:353208; Clone:G043H7; Biolegend: https://www.biolegend.com/fr-ch/products/brilliant-violet-421-anti-human-cd197-ccr7-antibody-7497
CD25 PC5 Cat:IM2646; Clone:B1.49.9; Beckman Coulter: https://www.beckman.de/reagents/coulter-flow-cytometry/antibodies-and-kits/single-color-antibodies/cd25/IM2646
CD56 PC5 Cat:A07789; Clone:N901; Beckman Coulter: https://www.beckman.es/reagents/coulter-flow-cytometry/antibodies-and-kits/single-color-antibodies/cd56/A07789
CD14 PC5 Cat:A70204; Beckman Coulter: https://www.beckman.com/reagents/coulter-flow-cytometry/antibodies-and-kits/single-color-antibodies/cd14/a70204
CD19 FITC Cat:555412; Clone:HIB19; BD Biosciences: https://www.bdbiosciences.com/en-ch/products/reagents/flow-cytometry-reagents/research-reagents/single-color-antibodies-ruo/fitc-mouse-anti-human-cd19.555412
ICOS Pacific Blue Cat:313522; Clone:H4A3; Biolegend: https://www.biolegend.com/fr-ch/products/pacific-blue-anti-human-mouse-rat-cd278-icos-antibody-7373
CD25 PE Cat:555432; Clone:M-A251; BD Biosciences: https://www.bdbiosciences.com/en-ch/products/reagents/flow-cytometry-reagents/research-reagents/single-color-antibodies-ruo/pe-mouse-anti-human-cd25.555432
Granzyme A APC Cat:507220; Clone CB9; Biolegend: https://www.biolegend.com/fr-ch/products/apc-anti-human-granzyme-a-antibody-15038
Granzyme B PE Cat:396406; Clone QA18A28; Biolegend: https://www.biolegend.com/fr-ch/products/pe-anti-human-mouse-granzyme-b-recombinant-antibody-17396
Perforin APC/Cyanine7 Cat:308128; Clone dG9; Biolegend: https://www.biolegend.com/fr-ch/products/apc-cyanine7-anti-human-perforin-antibody-13027
TNFα BV785 Cat:502948; Clone MAb11; Biolegend: https://www.biolegend.com/fr-ch/products/brilliant-violet-785-anti-human-tnf-alpha-antibody-12027
IL-10 PE/Cyanine7 Cat:501404; Clone JES3-9D7; Biolegend: https://www.biolegend.com/fr-ch/products/pe-anti-human-il-10-antibody-1341
IL-17A BV605 Cat:512326; Clone BL168; Biolegend: https://www.biolegend.com/fr-ch/products/brilliant-violet-605-anti-human-il-17a-antibody-7879
IFNγ BUV395 Cat:563563; Clone B27; BD Biosciences: https://www.bdbiosciences.com/en-ch/products/reagents/flow-cytometry-reagents/research-reagents/single-color-antibodies-ruo/buv395-mouse-anti-human-ifn.563563
IL-4 BV711 Cat:564112; Clone MP4-25D2; BD Biosciences: https://www.bdbiosciences.com/en-ch/products/reagents/flow-cytometry-reagents/research-reagents/single-color-antibodies-ruo/bv711-rat-anti-human-il-4.564112
IL-22 BUV737 Cat:367-7229-42; Clone 22URTI; Thermo Fisher Scientific: https://www.thermofisher.com/antibody/product/IL-22-Antibody-clone-22URTI-Monoclonal/367-7229-42

Flow Cytometry

Plots

Confirm that:

☒ The axis labels state the marker and fluorochrome used (e.g. CD4-FITC).

☒ The axis scales are clearly visible. Include numbers along axes only for bottom left plot of group (a 'group' is an analysis of identical markers).

☒ All plots are contour plots with outliers or pseudocolor plots.

☒ A numerical value for number of cells or percentage (with statistics) is provided.

Methodology

Sample preparation

PBMCs were isolated with Ficoll-Paque Plus (GE Healthcare). Monocytes were enriched by positive selection using CD14-coated microbeads (Miltenyi Biotec). From the CD14– cell fraction, memory CD4+ and CD8+ total cells were sorted to over 98% purity on a FACSAria Fusion (BD) excluding CCR7+CD45RA+, CD25bright, CD14+, CD56+ cells as well as either CD8+ cells (for memory CD4+ T cell enrichment) or CD4+ cells (for memory CD8+ T cell enrichment) according to the gating strategy shown in Extended Data Figure 1a. For intracellular cytokine staining, clones were restimulated with Phorbol-12-myristat-13-acetat (PMA) and Ionomycin in the presence of brefeldin A (all from Sigma-Aldrich), fixed and permeabilized with Cytofix/Cytoperm (BD Biosciences) according to the manufacturer's instructions.

Instrument

BD FACSAria Fusion
BD LSRFortessa

Software	BD FACS Diva (v9.0) was used for acquisition of samples and Flow-Jo (v10.8.1) for analsyis.
Cell population abundance	Purity of the relevant cell populations (CD4+ and CD8+ memory T cells) was checked after sorting at BD LSRFortessa instrument and found to be >98%.
Gating strategy	CD4+ and CD8+ total memory cells were sorted from CD14- fractions with BD FACSAria Fusion instrument excluding excluding CCR7+CD45RA+ double positive, CD25bright, CD14+ and CD56+ cells s well as either CD8+ cells (for memory CD4+ T cell enrichment) or CD4+ cells (for memory CD8+ T cell enrichment) according to the gating strategy shown in Extended Data Figure 1a. CD4+ T cells from CSF and nerve biopsy were sorted from T cell lines expanded in vitro using a BD FACSAria Fusion instrument as CD3+CD4+CD8-–CD19–CD56– .

☒ Tick this box to confirm that a figure exemplifying the gating strategy is provided in the Supplementary Information.
